# Supplementary material for: A small molecule HIF-1α stabilizer that accelerates diabetic wound healing
Source: Nat Commun. 2021 Jun 7;12:3363. doi: 10.1038/s41467-021-23448-7 (PMC8184911; doi:10.1038/s41467-021-23448-7)
Supplement: Supplementary file 1 — Reporting Summary [file 41467_2021_23448_MOESM1_ESM.pdf]

nature research

Corresponding author(s): Chung-Hang Leung

Last updated by author(s): Feb 21, 2021

Reporting Summary

Nature Research wishes to improve the reproducibility of the work that we publish. This form provides structure for consistency and transparency in reporting. For further information on Nature Research policies, see our Editorial Policies and the Editorial Policy Checklist.

Statistics

For all statistical analyses, confirm that the following items are present in the figure legend, table legend, main text, or Methods section.

n/a	Confirmed	
☐	☒	The exact sample size (n) for each experimental group/condition, given as a discrete number and unit of measurement
☐	☒	A statement on whether measurements were taken from distinct samples or whether the same sample was measured repeatedly
☐	☒	The statistical test(s) used AND whether they are one- or two-sided<br>*Only common tests should be described solely by name; describe more complex techniques in the Methods section.*
☐	☒	A description of all covariates tested
☐	☒	A description of any assumptions or corrections, such as tests of normality and adjustment for multiple comparisons
☐	☒	A full description of the statistical parameters including central tendency (e.g. means) or other basic estimates (e.g. regression coefficient) AND variation (e.g. standard deviation) or associated estimates of uncertainty (e.g. confidence intervals)
☐	☒	For null hypothesis testing, the test statistic (e.g. F, t, r) with confidence intervals, effect sizes, degrees of freedom and P value noted<br>*Give P values as exact values whenever suitable.*
☒	☐	For Bayesian analysis, information on the choice of priors and Markov chain Monte Carlo settings
☒	☐	For hierarchical and complex designs, identification of the appropriate level for tests and full reporting of outcomes
☒	☐	Estimates of effect sizes (e.g. Cohen's d, Pearson's r), indicating how they were calculated

Our web collection on statistics for biologists contains articles on many of the points above.

Software and code

Policy information about availability of computer code

Data collection

1. CD spectra were recorded on a JASCO-815 spectropolarimeter (JASCO spectroscopy & Chromatography Technology, JASCO-815)
2. Fluorescence-based protein thermal shift assay was performed using a real-time PCR setup (Thermo Fisher Scientific, QuantStudio™ 7 Flex Real-Time PCR System)
3. ITC experiments were carried in a MicroCal PEAQ-ITC Isothermal Titration Calorimeter (Malvern Panalytical Ltd, Serial: MAL1197766)
4. The binding affinities of inhibitors to recombinant VBC were measured by biolayer interferometry (Fortebio, OctetRed 96)
5. Fluorescence polarization signal was collected by using a microplate reader (Molecular Devices, LLC, SpectraMax M5)
6. Inductively coupled plasma mass spectrometry (ICP-MS) study was collected by using ICP-MS (Thermo Fisher Scientific, Thermo iCAP Qs)
7. Skin perfusion images were captured using a laser Doppler imager (Perimed AB, PeriCam PSIZR-20014)
8. Cellular thermal shift study was performed using ChemiDocTM MP Imaging System (Bio-Rad Laboratories, Inc., Serial: 734BR2942)
9. Skin perfusion detection was collected by Perimed PeriFlux System 5000 (Perimed, Stockholm, Sweden)
10. Proteasome activity detection was determined using commercial Proteasome Activity Fluorometric Assay Kit (BioVision Incorporated, CA, USA)
11. Oxygen consumption was determined using a commercial oxygen consumption rate assay kit (Cayman Chemical, MI, USA)

Data analysis

Statistical analysis was performed using GraphPad Prism 6.0 software (GraphPad company). All experimental data were presented as mean ± SD (standard deviation), and significant differences between groups were determined using a one-way analysis of variance (ANOVA) unless otherwise noted. The Setup MicroCal PEAQ-ITC Analysis Software were used for ITC data analysis (Malvern Panalytical Ltd, Serial: MAL1197766). Octet analysis software was used for the binding affinity analysis. Wound area and Western blot band intensity was quantified using ImageJ (National Institutes of Health, version 1.52a)

For manuscripts utilizing custom algorithms or software that are central to the research but not yet described in published literature, software must be made available to editors and reviewers. We strongly encourage code deposition in a community repository (e.g. GitHub). See the Nature Research guidelines for submitting code & software for further information.

Data

Policy information about availability of data

All manuscripts must include a data availability statement. This statement should provide the following information, where applicable:

- Accession codes, unique identifiers, or web links for publicly available datasets
- A list of figures that have associated raw data
- A description of any restrictions on data availability

The data that support the findings of this study are available from the corresponding author upon reasonable request. The source data underlying Figs. 1b, 1d, 2a, 2b, 2c, 2d, 2e, 2f, 3a, 3b, 3c, 5a, Supplementary Figures 5a-5b, Supplementary Figure 6b, Supplementary Figures 6e-6f, Supplementary Figures 7a-7c, Supplementary Figures 8a-8b, Supplementary Figure 14a and Supplementary Figure 15a are provided as a Source Data file.

Field-specific reporting

Please select the one below that is the best fit for your research. If you are not sure, read the appropriate sections before making your selection.

☒ Life sciences ☐ Behavioural & social sciences ☐ Ecological, evolutionary & environmental sciences

For a reference copy of the document with all sections, see nature.com/documents/nr-reporting-summary-flat.pdf

Life sciences study design

All studies must disclose on these points even when the disclosure is negative.

Sample size	Sample size was estimated based on previous experiments and publications from the group (Li et al. British Journal of Pharmacology, 2018; Yang et al. Angewandte Chemie International Edition, 2018). Variations between samples were also used to determine the suitability of the sample size. Following the principles of the 3 R's, several regions were analyzed per animal allowing comparable levels of data to be obtained with fewer animals ("reduction").
Data exclusions	No data were excluded from analyses in the experiments.
Replication	Cellular and in vitro experiments were performed at least twice as shown in the source data unless otherwise noted in the manuscript. In vivo efficacy was tested 2 or more times. Replicate experiments were successful.
Randomization	In animal studies, we randomly allocated animals into groups such that as animals were added to the experiment, the numbers of animals in each group did not significantly differ. For all remaining in vitro and in cellulo assays, plate design was chosen to minimize manipulation time. Blanks and functional controls were consistently included for each experiment involving quantitative measurements, to reduce the effect of confounders and covariates.
Blinding	We needed to investigate the difference between different groups, and such difference had not been known. Thus we did not use blinding in the study.

Reporting for specific materials, systems and methods

We require information from authors about some types of materials, experimental systems and methods used in many studies. Here, indicate whether each material, system or method listed is relevant to your study. If you are not sure if a list item applies to your research, read the appropriate section before selecting a response.

Materials & experimental systems

n/a	Involved in the study	
☐	☒	Antibodies
☐	☒	Eukaryotic cell lines
☒	☐	Palaeontology and archaeology
☐	☒	Animals and other organisms
☒	☐	Human research participants
☒	☐	Clinical data
☒	☐	Dual use research of concern

Methods

n/a	Involved in the study	
☒	☐	ChIP-seq
☒	☐	Flow cytometry
☒	☐	MRI-based neuroimaging

Antibodies

Antibodies used	VEGF antibody (Mouse, Novus Biologicals NB100-664)<br>HIF-1α antibody (Rabbit, Novus Biologicals NB100-479)<br>HIF-2α antibody (Rabbit, Novus Biologicals NB100-122)<br>EPO antibody (Rabbit, abcam ab129452)

	VHL antibody (Rabbit, GeneTex GTX101087)<br>Elongin B antibody (Mouse, Santa Cruz Biotechnology, Inc. sc-133090)<br>PHD2 antibody (Rabbit, Bethyl Laboratories, Inc. A300-322A)<br>PHD3 antibody (Rabbit, Bethyl Laboratories, Inc. A300-327A)<br>HIF-1α antibody (Rabbit, Cell Signaling Technology 36169)<br>CD31 antibody (Rabbit, ABclonal A3181)<br>α-tubulin antibody (Mouse, Santa Cruz Biotechnology sc-8035)<br>β-actin antibody (Rabbit, Cell Signaling Technology 4967L)<br>Hydroxy-HIF-1α antibody (Rabbit, Cell Signaling Technology 3434S)<br>GLUT1 antibody (Rabbit, Cell Signaling Technology 12939S)<br>COX-2 antibody (Rabbit, Cell Signaling Technology 4842S)
Validation	VEGF antibody (Mouse, Novus Biologicals NB100-664) has been validated for Western Blotting and immunofluorescence, and was used in 57 publications in various application (e.g. PMID: 32016463).<br>HIF-1α antibody (Rabbit, Novus Biologicals NB100-479) has been validated for Western Blotting in 225 publications (e.g. PMID: 32292512).<br>HIF-2α antibody (Rabbit, Novus Biologicals NB100-122) has been validated for Western Blot, Chromatin Immunoprecipitation, Immunohistochemistry-Paraffin, and was used in 655 publications in various application (e.g. PMID: 33024108).<br>EPO antibody (Rabbit, abcam ab129452) has been validated for Western Blot human Colon Cancer HCT116 Cells (PMID: 30678221).<br>VHL antibody (Rabbit, GeneTex GTX101087) has been validated for Western Blot,Chromatin Immunoprecipitation, and Immunohistochemistry-Paraffin, and was used in 6 publications in cellular lysates. (e.g. PMID: 26735018).<br>Elongin B antibody (Mouse, Santa Cruz Biotechnology, Inc. sc-133090) has been validated for Western Blot and Chromatin Immunoprecipitation in Human embryonic kidney 293 cells., and was used in 2 publications (e.g. PMID: 29386580).<br>PHD2 antibody (Rabbit, Bethyl Laboratories, Inc. A300-322A) has been validated for Western Blot and Chromatin Immunoprecipitation in Human Cervical Adenocarcinoma HeLa cells (PMID: 30801039), and was used in 18 publications.<br>PHD3 antibody (Rabbit, Bethyl Laboratories, Inc. A300-327A) has been validated for Western Blot and Chromatin Immunoprecipitation in Human cellular lysates (PMID: 29018234), and was used in 18 publications.<br>HIF-1α antibody (Rabbit, Cell Signaling Technology 36169) has been validated for Western Blot, Chromatin Immunoprecipitation, Immunohistochemistry-Paraffin, and was used in 50 publications in various application (e.g. PMID: 32908121).<br>CD31 antibody (Rabbit, ABclonal A3181) has been validated for Western blotting, Immunohistochemistry and Immunoprecipitation, and was used in 13 publications in various application (e.g. PMID: 29502978).<br>α-tubulin antibody (Mouse, Santa Cruz Biotechnology sc-8035) has been validated for Western blotting, Immunohistochemistry and Immunoprecipitation, and was used in 1226 publications in various application (e.g. PMID: 33523920).<br>β-actin antibody (Rabbit, Cell Signaling Technology 4967L) has been validated for Western blotting and was used in 1769 publications in various application (e.g. PMID: 33080033).<br>Hydroxy-HIF-1α antibody (Rabbit, Cell Signaling Technology 3434S) has been validated for Western blotting, Immunofluorescence and Immunoprecipitation, and was used in 58 publications in various application (e.g. PMID: 32908121).<br>GLUT1 antibody (Rabbit, Cell Signaling Technology 12939S) has been validated for Western blotting and Immunoprecipitation, and was used in 47 publications in various application (e.g. PMID: 33147469).<br>COX-2 antibody (Rabbit, Cell Signaling Technology 4842S) has been validated for Western blotting and Immunofluorescence, and was used in 116 publications in various application (e.g. PMID: 31860812).

Eukaryotic cell lines

Policy information about cell lines

Cell line source(s)	Human embryonic kidney HEK293 cells (CRL-1573, RRID: CVCL_0045) and Human Kidney cancer A498 cells (HTB-44, RRID: CVCL_1056) were obtained from ATCC. More information in Methods section.
Authentication	These cell lines were authenticated by cell vitality test, isozyme detection, DNA fingerprinting, and mycoplasma detection.
Mycoplasma contamination	The cell lines were detected for mycoplasma contamination and no mycoplasma was found.
Commonly misidentified lines<br>(See ICLAC register)	No commonly misidentified cell lines were used.

Animals and other organisms

Policy information about studies involving animals; ARRIVE guidelines recommended for reporting animal research

Laboratory animals	C57BL/6J WT Mice were purchased from the animal facility of Faculty of Health Sciences, University of Macau. Male db/db mice were purchased from the Model Animal Research Center of Nanjing University (Nanjing, China).<br>Male, 10–12 weeks.<br>All mice were housed in the animal facility of University of Macau, maintained at 23 ± 1 ºC (50% ± 5% relative humidity) with 12 h light/dark cycles with free access to water and regular chow diet.
Wild animals	This study did not involve wild animals.
Field-collected samples	This study did not involve field-collected samples.

Ethics oversight	All animal experiments were approved by the Animal Ethical and Welfare Committee of University of Macau (No. ICMS-AEC-2014-06). All experiments complied with all relevant ethical regulations.

Note that full information on the approval of the study protocol must also be provided in the manuscript.
