# Supplementary material for: A small molecule HIF-1α stabilizer that accelerates diabetic wound healing
Source: Nat Commun. 2021 Jun 7;12:3363. doi: 10.1038/s41467-021-23448-7 (PMC8184911; doi:10.1038/s41467-021-23448-7)
Supplement: Supplementary file 2 — Supporting Information [file 41467_2021_23448_MOESM2_ESM.pdf]

Supporting Information

A small molecule HIF-1α stabilizer that accelerates diabetic wound healing

Guodong Li,[1,#] Chung-Nga Ko,[2,#] Dan Li,[1,#] Chao Yang,[1,#] Wanhe Wang,[2] Guan-Jun Yang,[1] Carmelo Di Primo,[3,4] Vincent Kam Wai Wong, [5] Yaozu Xiang,[6] Ligen Lin,[1,*] Dik-Lung Ma,[2,*] and Chung-Hang Leung[1,*]

[1] State Key Laboratory of Quality Research in Chinese Medicine, Institute of Chinese Medical Sciences, University of Macau, Macao, China. [*]E-mail: duncanleung@um.edu.mo; ligenl@um.edu.mo

[2] Department of Chemistry, Hong Kong Baptist University, Kowloon Tong, Hong Kong, China. [*]E-mail: edmondma@hkbu.edu.hk

[3] Université de Bordeaux, Laboratoire ARNA, 146 rue Léo Saignat, Bordeaux, France.

[4] INSERM U1212, CNRS UMR 5320, IECB, 2 rue Robert Escarpit, Pessac, France.

[5] State Key Laboratory of Quality Research in Chinese Medicine, Macau University of Science and Technology, Macao, China.

[6] Shanghai East Hospital of Tongji University, School of Life Sciences and Technology, Tongji University, Shanghai, China.

[#]These authors contributed equally: Guodong Li, Chung-Nga Ko, Dan Li and Chao Yang.

Materials and cell lines. Fetal bovine serum (FBS) and Dulbecco's Modified Eagle's Medium (DMEM) were available from Gibco BRL. Antibodies against HIF-1α (1:200; NB100-479, Novus Biologicals), HIF-2α (1:500; NB100-122, Novus Biologicals), VEGF (1:500; NB100-664, Novus Biologicals), VHL (1:1000; GTX101087, GeneTex), EPO (1:200; ab129452, abcam), Elongin B (1:1000; sc-133090, Santa Cruz Biotechnology), PHD2 (1:1000; A300-322A, Bethyl Laboratories), PHD3 (1:1000; A300-32&A, Bethyl Laboratories), HIF-1α (1:1000; 36169, Cell Signaling Technology), α-tubulin (1:1000; sc-8035, Santa Cruz Biotechnology), β-actin (1:1000; 4967L, Cell Signaling Technology), Hydroxy-HIF-1α (1:1000; 3434S, Cell Signaling Technology), GLUT1 (1:1000; 12939S, Cell Signaling Technology), COX-2 (1:1000; 4842S, Cell Signaling Technology), VHL antibody (1:200; NB100-479, Novus Biologicals), CD31 antibody (1:1000; A3181, ABclonal). Fluorescent secondary antibody, DAPI, nuclear and cytoplasmic protein extraction kit were purchased from Thermo Fisher Scientific. Luciferase reporter assay system was purchased from Promega. VEGF ELISA kit was purchased from NeoBioscience. VH298 in FTS assay was purchased from Abcam (Catalog No. ab230370). All other reagents and chemicals were obtained from commercial sources and used as received. Perimed PeriFlux System 5000 (Perimed, Stockholm, Sweden). Human embryonic kidney HEK293 cells (CRL-1573, RRID: CVCL_0045) and Human Kidney cancer A498 cells (HTB-44, RRID: CVCL_1056) were obtained from ATCC and maintained in Dulbecco's modified Eagle's medium (DMEM, Gibco, USA) containing 10% fetal bovine serum (FBS) at 37 °C and in the presence of 5% CO_2.

VBC protein expression and purification. Fragment of VHL coded sequence (corresponding to the 54th-213th amino acids of VHL protein) was first amplified from a VHL-pGEX-2TK plasmid, a gift from Dr. William Kaelin (Addgene plasmid # 20790), with primers with recognized sites of restriction endonucleases *EcoR*I and *Xho*I (Forward, 5'-CCG**GAATTC**ATGGAGGCCGGGCGGCCGCG-3'; reverse, 5'-CCG**CTCGAG**ATCTCCCATCCGTTGATGTGCAAT-3') and inserted into T-Vector pMD™19 (Simple). The recombinant plasmid and pET28a vector were digested,

extracted, and ligated to give rise to a pET28a_VHL plasmid with a six Histidine-tag. pACYC-1, an Elongin B and Elongin C co-expression plasmid, was a gift from Dr. Nicola Burgess-Brown (Addgene plasmid # 110274). Plasmids pET28a_VHL and pACYC-1 were cotransformed into the expression strain *E. coli* (BL21) (DE3), and verified by using PCR (VHL_{54-213} primers, Forward, 5'-CCGGAATTCATGGAGGCCGGGCGGCCGCG-3'; reverse, 5'-CCGCTCGAGATCTCCCATCCGTTGATGTGCAAT-3'; pACYC-1 primers: Forward, 5'-ATGATGTATGTCAAATTGATATCATCT-3'; reverse, 5'-CTAACAATCTAAGAAGTTCGCAGCCATC-3'). To obtain the VBC complex, a preculture from one colony of BL21(DE3)(pET28a_VHL + pACYC_1) was grown overnight at 37°C in lysogeny broth (LB) medium supplemented with 50 μg/ml kanamycin and chloramphenicol (Kan^+/Chl^+), respectively. The human recombinant VBC protein were expressed and purified as described previously[1], with minor modifications. Brief, the *E. coli* BL21(DE3) containing plasmids pET28a_VHL and pACYC_1 were cultured in LB medium with Kan^+/Chl^+ (50 μg/ml), and grew at 37°C until the OD_{600} of 0.6-0.8. Then the bacterial solutions were induced with 1 mM isopropyl-β-D-thiogalactopyranoside (IPTG) at 37°C for 4 h. The recombinant *E. coli* cells were harvested by centrifugation at 5000 g for 20 min and then homogenized by sonication in buffer (20 mM Tris-HCl, 400 mM NaCl, 5 mM imidazole, PH 7.4) and precleared lysates were applied to His GraviTrap columns (GE Healthcare, Catalog No. 11-0033-99), following kit protocols for purifications. The protein was assayed by using pull down assay and circular dichroism measurement.

Pull down assay[2-5]. Briefly, 120 μL Ni-NTA agarose beads were transferred to a gravity flow column, immobilize 50 μg His-tag VHL protein with 400 μL equilibrium buffer and load onto collect column. 200 μL bacterial extract (before and after induction of BL21(DE3)/Elongin BC) with 200 μL equilibrium buffer was loaded onto the gravity flow column and then incubated 1 h at 4 °C under agitation and then 10 min on ice without agitation buffer. The column was washed with 400 μL equilibrium buffer and

centrifugation for 1 min at 1000 × g at 4 °C, the gravity flow column was added 400 μL equilibrium buffer containing 50 mM imidazole to wash column three times. Elute by loading 80 μL elution buffer contained 500 mM imidazole to column and incubate 10 min at 4 °C, followed by centrifugation for 1 min at 1000 × g at 4 °C. The fractions were analyzed by Western blotting using VHL antibody anti-VHL antibody (GeneTex, GTX101087, 1:1,000 dilution) and Elongin B Antibody (Santa Cruz Biotechnology, Inc., sc-133090, 1:1,000 dilution).

Circular dichroism measurement. Protein concentration was 5 μM in CD buffer (20 mM Tris, 200 mM NaCl, 1 mM DTT, pH 7.4). CD spectra were recorded on a JASCO-815 spectropolarimeter using 1 cm path length quartz cuvettes at 20 °C. Spectra were collected between 200 nm and 320 nm, using a data pitch length of 0.5 nm, bandwidth of 2 nm, averaging time of 5 s for each measurement, and an accumulation cycle of 3 runs per measurement. The smoothed curves were plotted using GraphPad Prism software. The data were baseline corrected using CD spectra of buffer alone. The buffer used for the experiment were filtered with 0.22 μm nylon membrane filter and degassed.

Fluorescence-based protein thermal shift assay. The protein thermal shift assay with purified protein was performed by using the GloMelt™ Thermal Shift Protein Stability Kit (Biotium, No. 33021−1). Briefly, purified human recombinant VBC protein was appropriately diluted. All assay experiments used 2.5 μL 8× GloMelt™ Dye and 100 μM of VH298 or **1a** up to a total volume of 20 μL with VBC protein. The PCR plates were sealed with an optical seal, shaken, and centrifuged after protein and compounds were added. Thermal scanning (25 to 95 °C at 0.2 °C/min) was performed using a real-time PCR setup (QuantStudio™ 7 Flex Real-Time PCR System).

Fluorescence polarization assay[7, 8]. The fluorescent ligand FAM-DEALA-Hyp-YIPD was firstly diluted with VBC buffer (100 mM Tris, 100 mM NaCl, 1 mM DTT, pH 7.0). For K_d determination of fluorescent peptides, the fluorescence polarization of a 2-fold serial dilution of 10 μM VBC in 125 nM of FAM-DEALA-Hyp-YIPD was prepared in

VBC buffer. For the dose response curves for complex **1a** or unlabelled peptide ligand, wells of a 384-well plate (Corning #3575) were added 9 μL of 1 μM VBC (450 nM final), 9 μL of 278 nM FAM-DEALAHyp-YIPD (125 nM final), and 2 μL of complex **1a** or peptide ligand (2-fold serial dilutions starting from 400 μM, 1% DMSO final). The reference FP response of **1a** was subtracted from the sample response with immobilized VBC protein. Control wells contained VBC and fluorescent peptide in the absence of compound (maximum signal), and fluorescent peptide in the absence of protein (background signal). Before reading the fluorescence polarization signal on a SpectraMax M5 microplate reader (Molecular Devices, excitation 485 nm, emission 520 nm), the plate was shaken for 1 minute, then centrifuged for 1 minute. K_d values were then back-calculated from the measured IC_{50} values as previously described[8].

Measurement of proteasome activity. The effect of complex **1a** on the proteasome activity was determined using commercial Proteasome Activity Fluorometric Assay Kit (BioVision Incorporated, CA, USA).

Measurement of oxygen consumption. The effect of complex **1a** on oxygen consumption in HEK293 living cells was determined using commercial oxygen consumption rate assay kit (Cayman Chemical, MI, USA).

Inductively coupled plasma mass spectrometry (ICP-MS) study. 10 μL of the skin samples harvested after 8 days from control and treatment groups were completely digested in 2 mL of a 68% HNO_3: H_2O_2 (v/v=4:1) solution, while the remainder of the lysate was quantified for protein by a bicinchoninic assay (BCA). The iridium content in skin samples was determined by ICP-MS with an iridium standard solution (from Sigma-Aldrich) and calculated as pg[iridium]/μg[protein].

Assessment of skin perfusion. A laser Doppler imager (PeriCam PSI System, Perimed AB, Stockholm, Sweden) was used to measure skin perfusion at the wound area of mice.

Quantitative RT-PCR. Total RNA of wound tissue was isolated using TRIzol Reagent (Invitrogen, Carlsbad, CA, USA), following the manufacturer's instructions. The cDNA was synthesized from 1 μg RNA using the SuperScript III First-Strand Synthesis System for qRT-PCR (Invitrogen). The qPCR experiments were conducted on Step-One plus real-time PCR System (Applied Biosystems, Thermo Fisher Scientific, Woolston Warrington, UK) using SYBR green PCR Master Mix with gene specific primers (Supplementary Table S2). *18S* RNA was used as an internal control.

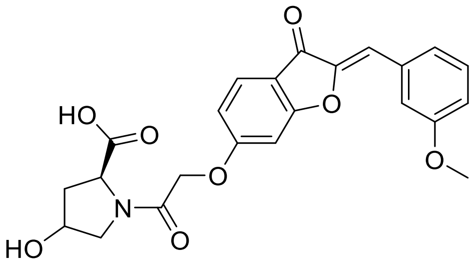

P1

Supplementary Figure 1. Chemical structure of **P1**.

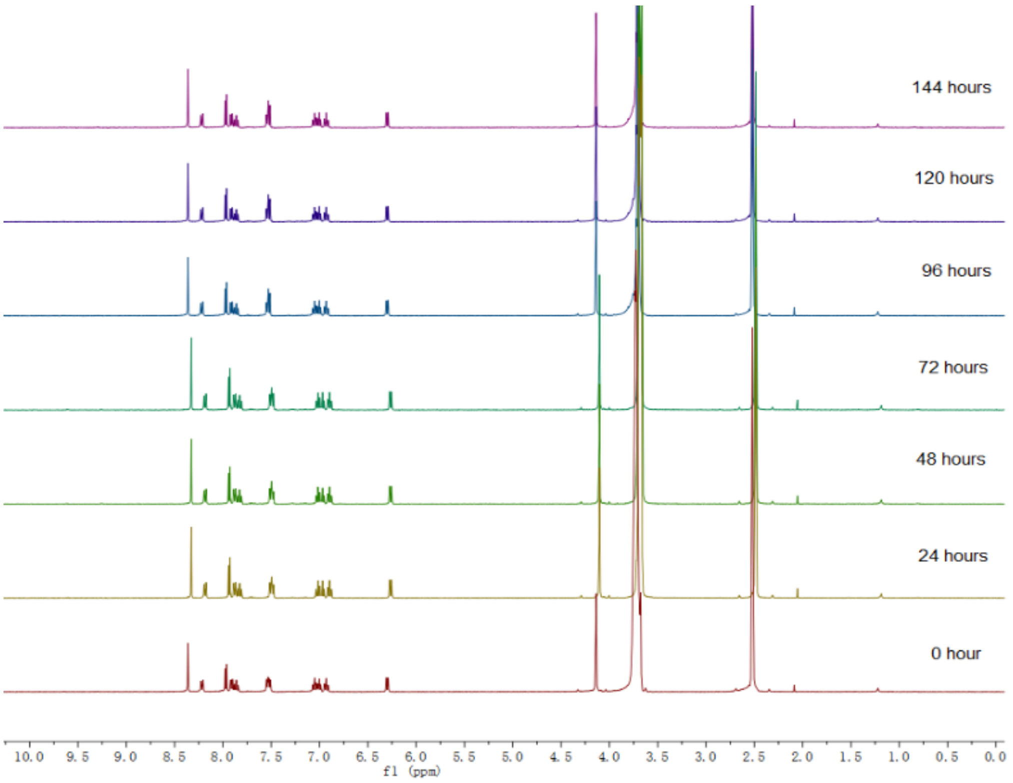

Supplementary Figure 2. ^{1}H NMR spectra of complex **1a** at a concentration of 5 mM in 90%[d_6]DMSO/10% D_2O at 298 K over 7 days.

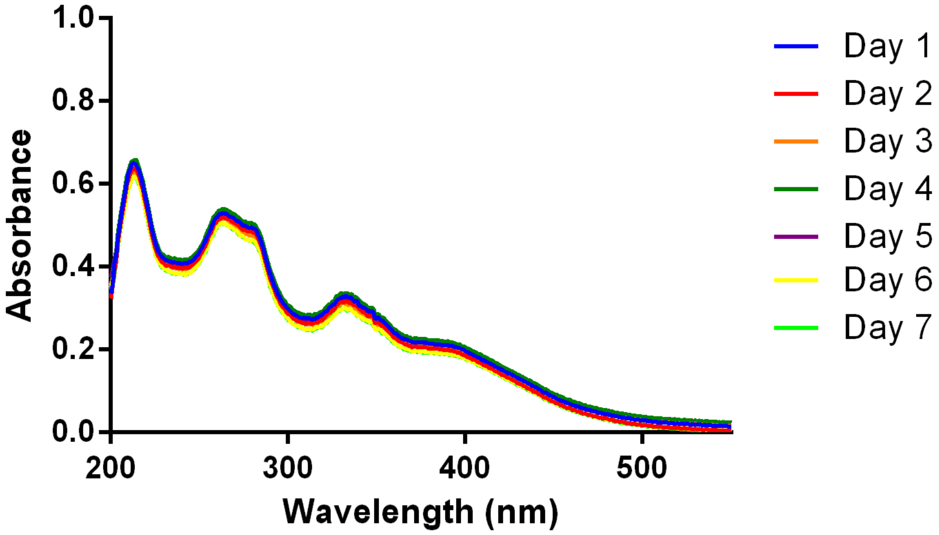

Supplementary Figure 3. UV/Vis absorption of complex **1a** at a concentration of 20 μM in 80% acetonitrile/20% Tris-HCl buffer (20 mM containing 20 mM NaCl, pH 7.5) at 298 K over 7 days.

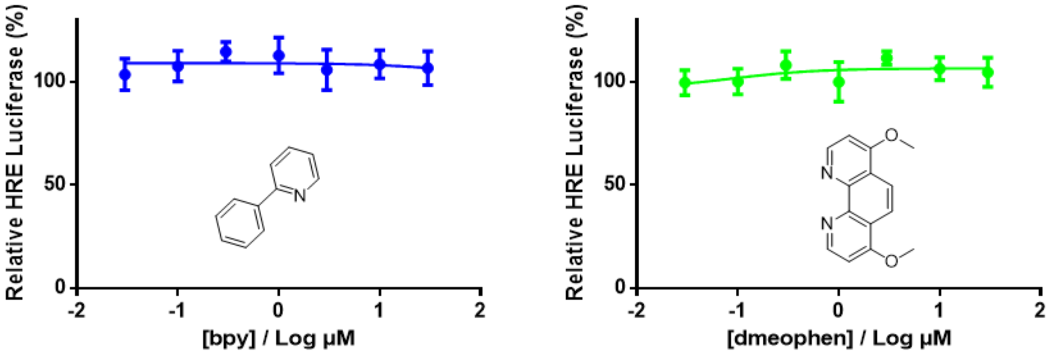

Supplementary Figure 4. Dose-dependent effect of ligands (a) 2-phenylpyridine (bpy) and (b) 4,7-dimethoxy-1,10-phenanthroline (dmeophen) on the VHL–HIF-1α interaction as determined *via* a dual luciferase reporter assay. HEK293 cells were treated with the indicated concentrations of ligands bpy or dmeophen for 8 h. Error bars represent the standard deviations of the results obtained from three independent experiments.

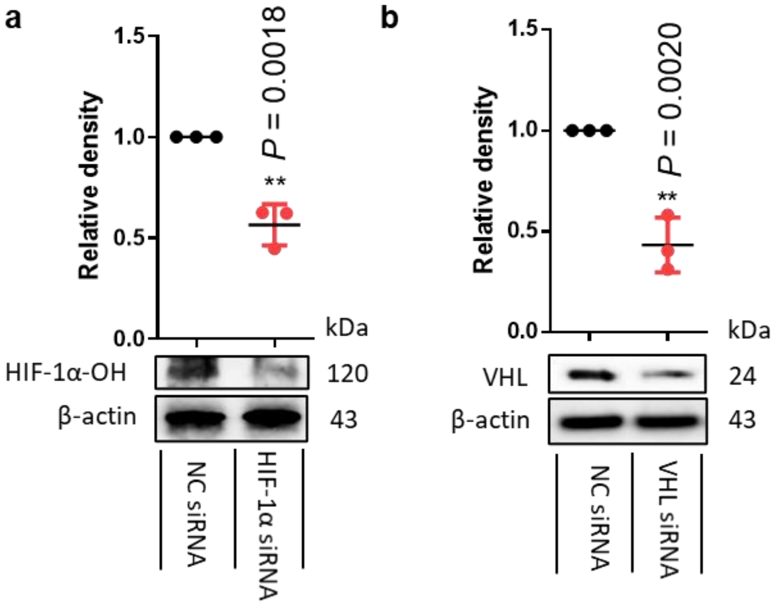

Supplementary Figure 5. Knockdown assay to verify the specificity of HIF-1α-OH and VHL antibodies. (a) The expression levels of HIF-1α-OH in HEK293 cells with or without knockdown HIF-1α. (b) The expression levels of VHL in HEK293 cells with or without knockdown VHL. HIF-1α siRNA (sense, 5'-CUGAUGACCAGCAACUUGA-3', antisense, 5'-UCAAGUUGCUGGUCAUCAG-3'), VHL siRNA (5'-ACACAGGAGCGCAUUGCACAU-3', antisense, 5'-AUGUGCAAUGCGCUCCUGUGU-3') [9, 10]and NC siRNA (Negative control (NC) siRNA (sense, 5'-UAGCGACUAAACACAUCAA-3', antisense, 5'-UUGAUGUGUUUAGUCGCUA- 3'). Data are expressed as means ± SD (n = 3 independent experiments). P values were calculated using a two-sided t-test. $^{**}P < 0.01$ vs. NC siRNA group. Source data are provided as a Source Data file.

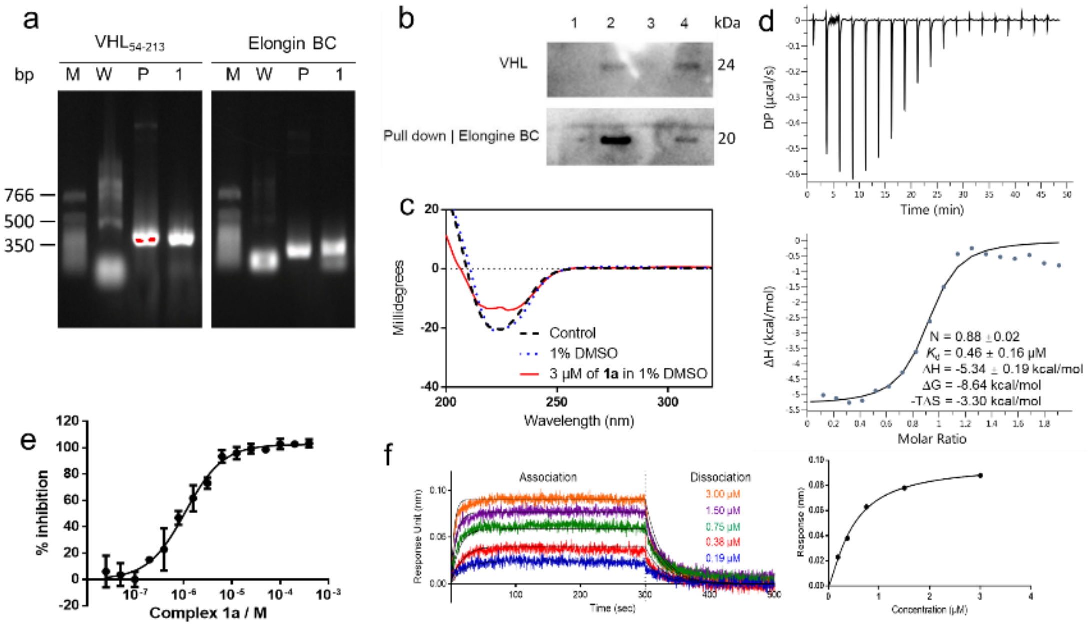

Supplementary Figure 6. (a) Agarose gel electrophoresis of DNA fragments from the colonies of BL21(DE3)(pET28a_VHL + pACYC_1) grown overnight at 37°C in lysogeny broth (LB) medium supplemented with 50 µg/ml kanamycin and chloramphenicol (Kan^+/Chl^+). Plasmids pET28a_VHL and pACYC-1 were co-transformed into the expression strain *E. coli* (BL21) (DE3), and verified by using PCR ($VHL_{54\text{-}213}$ primers, Forward, 5'-CCGGAATTCATGGAGGCCGGGCGGCCGCG-3'; reverse, 5'-CCGCTCGAGATCTCCCATCCGTTGATGTGCAAT-3'; Elongin BC primers: Forward, 5'-ATGATGTATGTCAAATTGATATCATCT-3'; reverse, 5'-CTAACAATCTAAGAAGTTCGCAGCCATC-3'). Lane M: low molecular weight DNA ladder; Lane W: PCR products of water; Lane P: PCR products of the partial sequences from plasmids pET28a_VHL (left, 498 bp) and pACYC-1 (right, 294 bp); Lane 1: the colony of BL21(DE3)(pET28a_VHL + pACYC_1). The results were successfully replicated. (b) The pull-down assay was performed by using a gravity flow column with Ni-NTA agarose beads. 50 µg His-tag VHL protein with 400 µL equilibrium buffer was immobilized in the column, and 200 µL bacterial extract with or without IPTG induction with 200 µL equilibrium buffer was loaded onto the gravity flow column. The gravity flow column was then washed, and the fractions eluted by elution buffer containing 500 mM imidazole were analyzed by Western blotting using VHL antibody (GeneTex, GTX101087, 1:1,000 dilution) and Elongin B Antibody (Santa Cruz Biotechnology, Inc., sc-133090, 1:1,000 dilution). Lane 1: Bacterial extract (BL21(DE3)/Elongin BC) without induction with IPTG; Lane 2: Bacterial extract (BL21(DE3)/Elongin BC) with 4h induction with IPTG; Lanes 3: 5 X diluted bacterial extract without induction with IPTG; Lanes 4: 5 X diluted bacterial extract with 4h induction with IPTG. The results were successfully replicated. (c) Circular dichroism of VBC complex with or without **1a** and 1% DMSO. CD spectroscopy measurements were carried in JASCO-815 spectropolarimeter at room temperature to evaluate the folding and stability of VBC complex. VBC was at a final concentration of 5 µM in CD buffer (pH 7.4). (d) ITC titration of hydroxylated HIF peptide DEALA-Hyp-YIPD (300

μM) into recombinant VBC complex (30 μM). ITC experiments were carried in a MicroCal PEAQ-ITC Isothermal Titration Calorimeter (Malvern Panalytical) and analyzed by using composite model with three control titrations. (e) A competitive fluorescence polarization binding assay was performed to evaluate the displacement of a fluorescent peptide (FAM-DEALAHyp-YIPD) from VBC (K_d = 421.50 ± 65.23 nM) by unlabelled HIF peptide DEALAHyp-YIPD. The IC_{50} of DEALAHyp-YIPD (K_d = 773.86 nM) was determined to be 1.09 μM in the presence of 125 nM of the fluorescent peptide and 450 nM of VBC using a four-parameter logistic equation. Data are expressed as means ± SD (n = 3 samples). (f) BLI kinetic analysis of the interaction between HIF-1α peptide, DEALAHyp-YIPD, and VBC. VBC was surface-immobilized to Ni-NTA biosensors. BLI sensorgrams showing the binding of HIF-1α peptide to surface-immobilized VBC. The K_d values for a 1:1 interaction were calculated from the kinetic fit (K_d = 0.66 ± 0.01 μM) and steady state fit (K_d = 0.72 ± 0.06 μM), respectively. The Ni-NTA biosensor tips coated with His-tagged VBC were dipped in increasing concentrations of peptide (0.19, 0.38, 0.75, 1.50, and 3.00 μM) to measure binding affinity of **1a** to VBC (K_{on} = 5.78 $\times 10^4$ $M^{-1}s^{-1}$) and subsequently moved to wells containing buffer to measure dissociation rates (K_{off} = 3.84 $\times 10^{-2}$ s^{-1}). Source data are provided as a Source Data file.

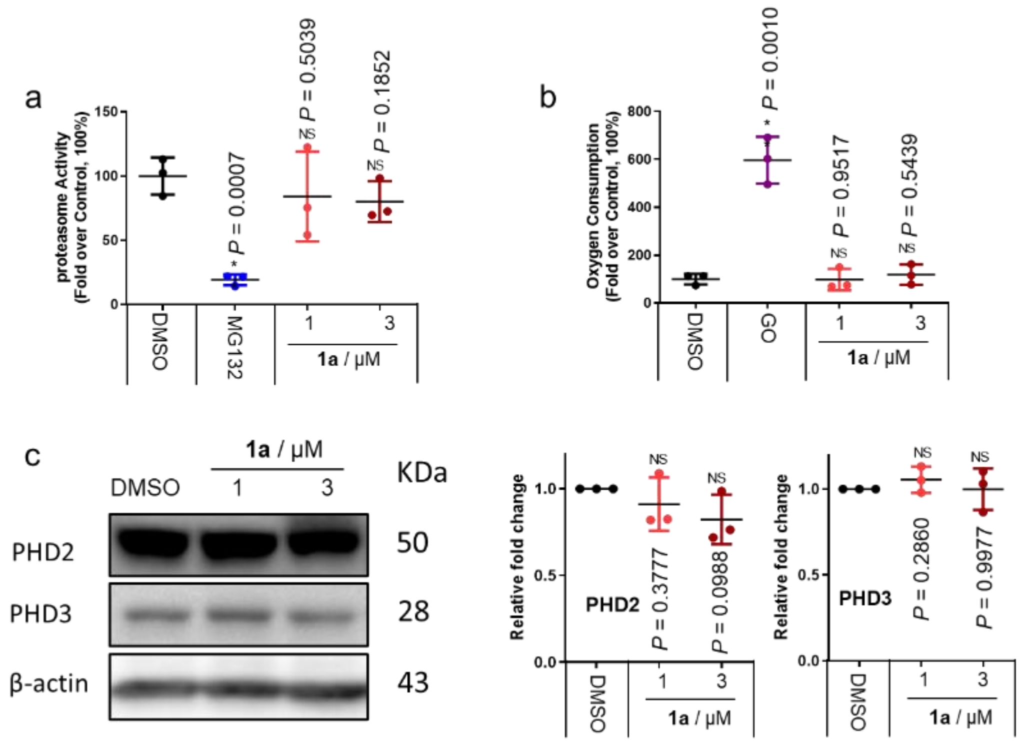

Supplementary Figure 7. Complex **1a** does not interfere with proteasome activity, oxygen consumption or PHD2/PHD3 levels. (a) A commercial fluorometric assay kit was used to monitor the effect of **1a** on proteasome activity. Homogenized HEK293 lysis samples were prepared by using 0.5 % NP-40 lysis buffer in PBS. 50 μL of cell extract was added to 50 μL of assay buffer. 1 μL of complex **1a** (0.1 and 0.3 mM) or proteasome inhibitor MG132 (1 mM) was added to one of the paired wells. The fluorescence intensity of the cells was measured at Ex/Em = 350/440 nm in a microplate reader after incubation at 37° C for 60 min in the dark. (b) Complex **1a** has no effect on the oxygen consumption in living HEK293 cells. HEK293 cells were seeded in a black bottom 96-well tissue culture plate and incubated overnight. After treatment with complex **1a** for 2 h, MitoXpress Xtra solution was added. 100 μL of HS Mineral Oil was added and the fluorescence intensity was monitored immediately at Ex/Em = 380/650 nm in a microplate reader. Glucose oxidase (GO) was used as a reference for oxygen depletion. (c) Immunoblotting assay to monitor the effect of **1a** on PHD2 and PHD3 levels. HEK293 cells were incubated with the indicated concentration of complex **1a** (1 and 3 μM) for 2 h. The protein samples were collected and detected by Western blotting using PHD2 or PHD3 antibodies. Data are expressed as means ± SD (*n* = 3 independent experiments). *P* values were calculated using a two-sided t-test. $^{**}P < 0.01$ vs. DMSO group. NS (not significant, $P > 0.05$) vs. DMSO group. Source data are provided as a Source Data file.

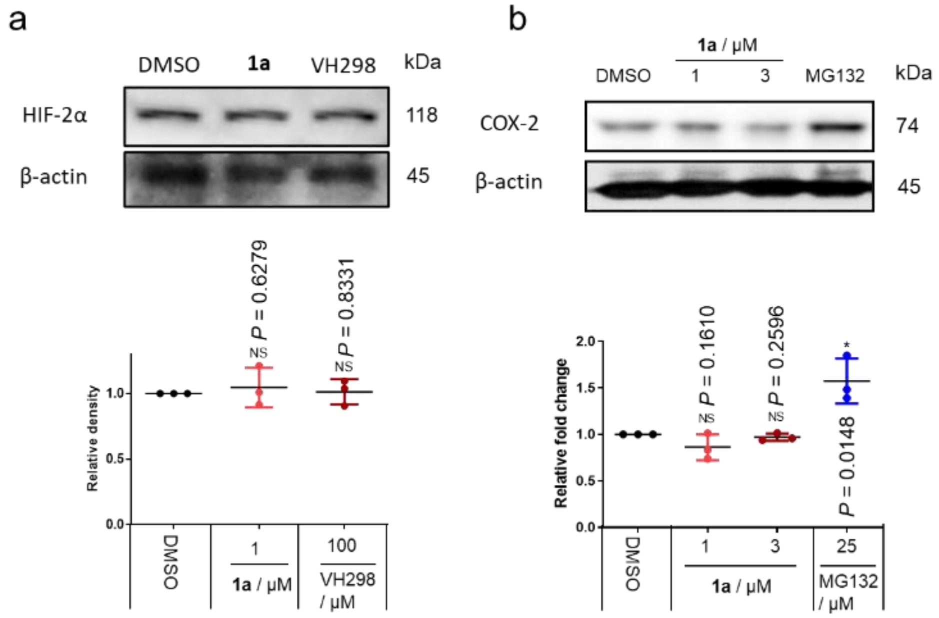

Supplementary Figure 8. Effect of complex 1a on specific proteins mediated by HIF-2α. Effect of complex **1a** on the levels of (a) HIF-2α and (b) COX-2 in HEK293 cells. HEK293 cells were treated with **1a** for 2 h. Cell lysates were collected and analyzed by the Western blotting. Data are expressed as means ± SD (n = 3 independent experiments). P values were calculated using a two-sided t-test. $^{*}P < 0.05$ vs. DMSO group. NS (not significant, $P > 0.05$) vs. DMSO group. Source data are provided as a Source Data file.

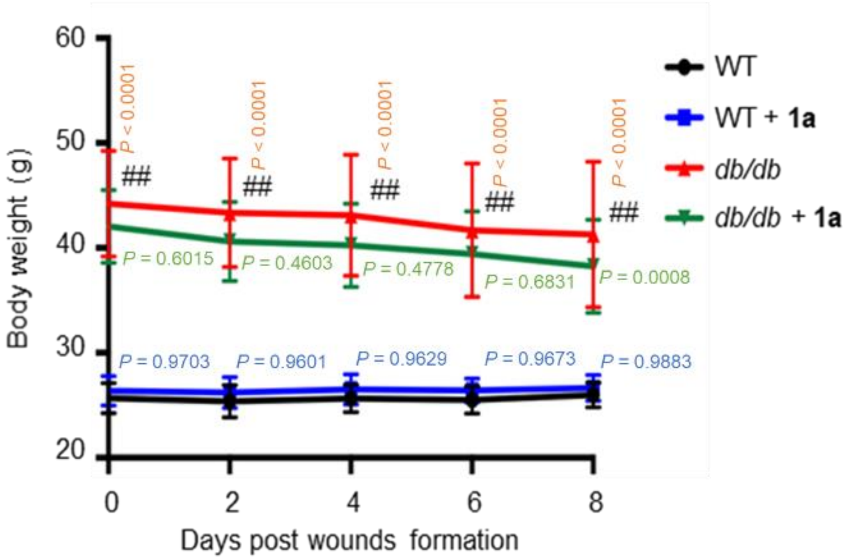

Supplementary Figure 9. Effects of complex **1a** on body weight of WT and *db/db* mice. Data are expressed as means ± SD (n = 5 mice). P values were calculated using a one-way ANOVA with Tukey's multiple comparison test. $^{\#\#}P < 0.01$ WT vs. *db/db* mice.

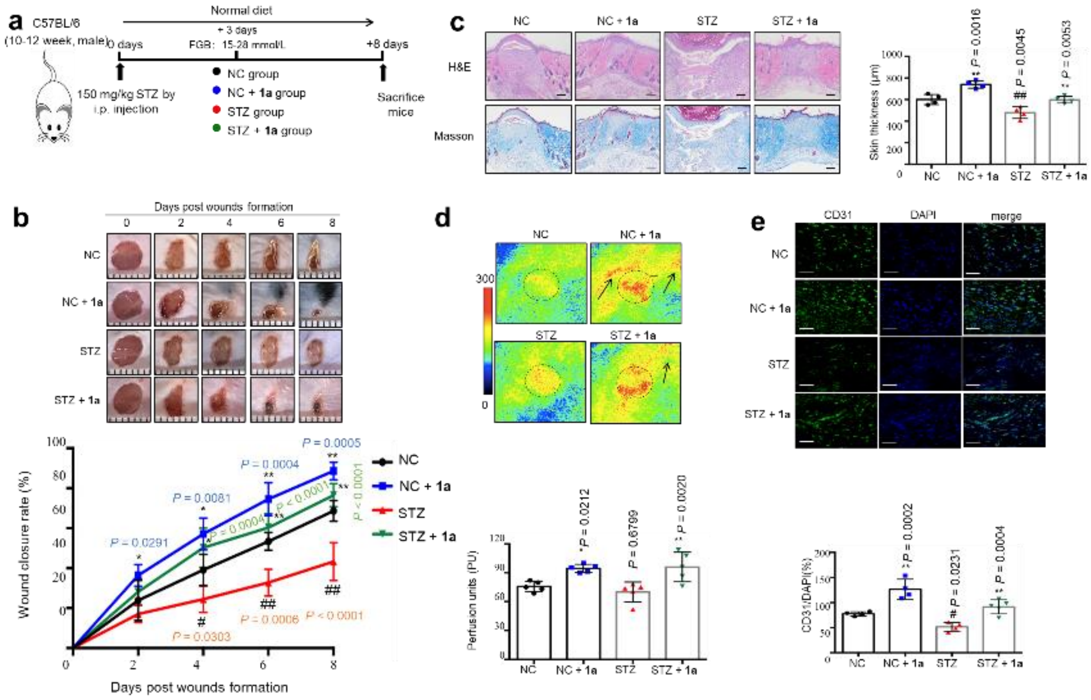

Supplementary Figure 10. Complex **1a** (1.25 mg/kg) accelerates wound closure in STZ-induced diabetic mice. (a) Timeline for *in vivo* experiments. (b) Image of representative wound (left) and wound closure rate (right) (n = 5 mice). (c) H&E and Masson's trichrome staining of dorsal skin section and skin thickness from the top of the epidermis to the bottom of the dermis in mice after 8 days post-injury (n = 4 mice). Scale bar = 100 μm. (d) Laser doppler imager in dorsal skin: representative images were shown for each group (left) and baseline perfusion on back skin of mice (right) after 2 days post-injury (dotted line circle represents the wound bed in mice of each group and arrow represents perfusion intensity (n = 5 mice). (e) CD31 and DAPI double staining in wound bed of mice after 8 days post-injury (n = 4 mice). Scale bar = 50 μm. Data are expressed as means ± SD. P values were calculated using a one-way ANOVA with Tukey's multiple comparison test. $^{\#}P < 0.05$, $^{\#\#}P < 0.01$ NC vs. STZ, $^{*}P < 0.05$, $^{**}P < 0.01$ **1a** vs. vehicle.

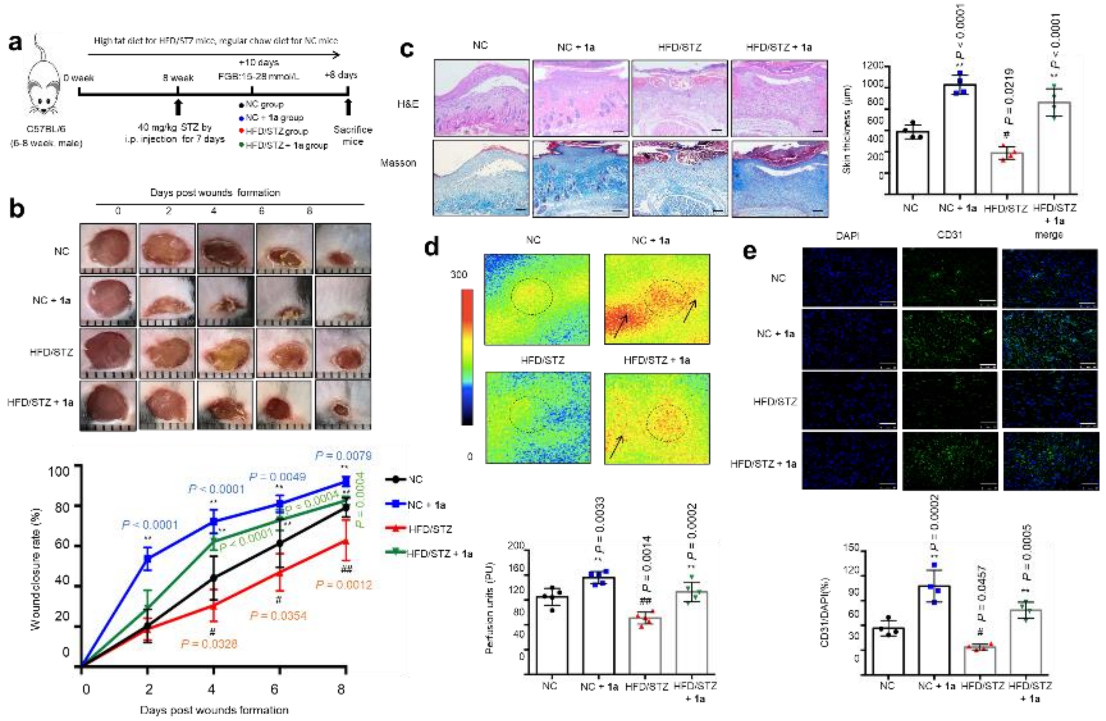

Supplementary Figure 11. Complex **1a** (1.25 mg/kg) accelerates wound closure in HFD/STZ induced diabetic mice. (a) Timeline for *in vivo* experiments. (b) Image of representative wound (left) and wound closure rate (right) (*n* = 5 mice). (c) H&E and Masson's trichrome staining of dorsal skin section and skin thickness from the top of the epidermis to the bottom of the dermis in mice after 8 days post-injury (*n* = 4 mice). Scale bar = 200 μm. (d) Laser doppler imager in dorsal skin: representative images were shown for each group (left) and baseline perfusion on back skin of mice (right) after 2 days post-injury (dotted line circle represents the wound bed in mice of each group and arrow represents perfusion intensity) (*n* = 5 mice). (e) CD31 and DAPI double staining in the wound bed of mice after 8 days post-injury (*n* = 4 mice). Scale bar = 50 μm. Data are expressed as means ± SD. *P* values were calculated using a one-way ANOVA with Tukey's multiple comparison test. $^{\#}P < 0.05$, $^{\#\#}P < 0.01$ HFD/STZ vs. NC, $^{**}P < 0.01$ **1a** vs. vehicle.

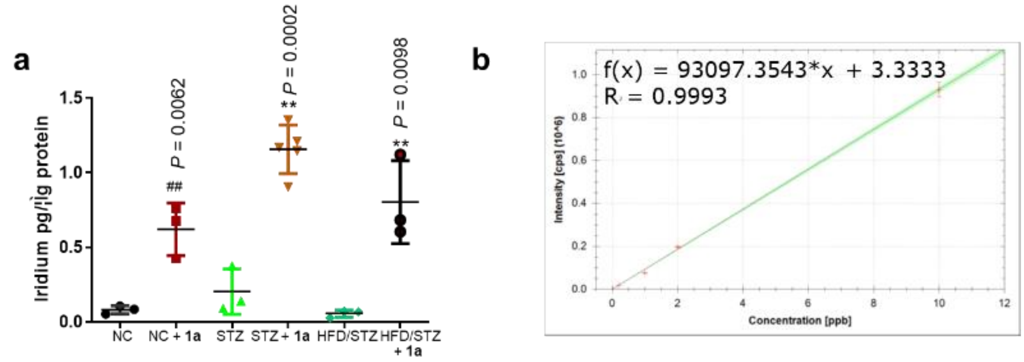

Supplementary Figure 12. (a) ICP-MS assay for iridium content in skin samples of dosed mice from both models. The vehicle and complex **1a** groups were intraperitoneally injected with vehicle (PEG 400: distilled water = 6:4, v/v) or 1.25 mg/kg complex **1a**, respectively every other day for 8 days, and skin samples were harvested. (b) Standard curve of iridium. Data are expressed as means ± SD ($n = 3$ mice for NC, NC + **1a**, STZ, HFD/STZ and HFD/STZ + **1a**, and 5 for STZ + **1a**). P values were calculated using a one-way ANOVA with Tukey's multiple comparison test. $^{\#\#}P < 0.01$ NC + **1a** vs. NC, $^{**}P < 0.01$ **1a** vs. vehicle, respectively.

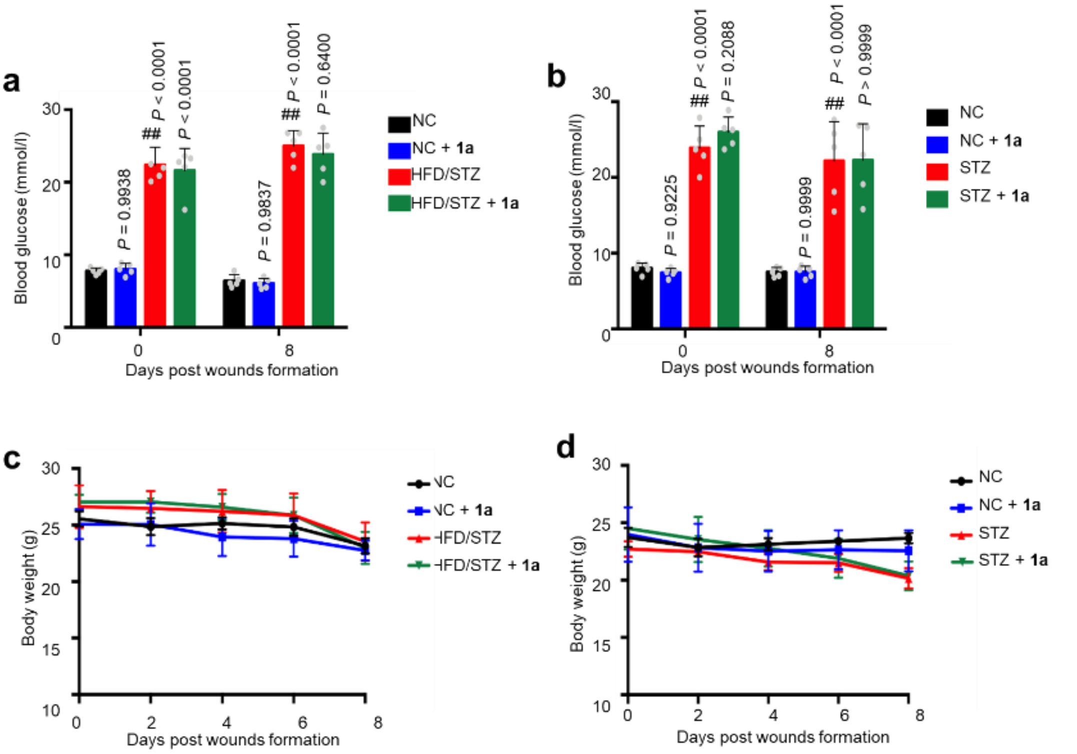

Supplementary Figure 13. Effects of complex **1a** on body weight and blood glucose. (a) Blood glucose level of NC and HFD/STZ induced diabetic mice. (b) blood glucose level of NC and STZ-induced diabetic mice. (c) body weight of NC and HFD/STZ induced diabetic mice. (d) body weight of NC and STZ-induced diabetic mice. Data are expressed as means ± SD ($n = 5$ mice). P values were calculated using a one-way ANOVA with Tukey's multiple comparison test. $^{\#\#}P < 0.01$ NC vs. diabetic mice.

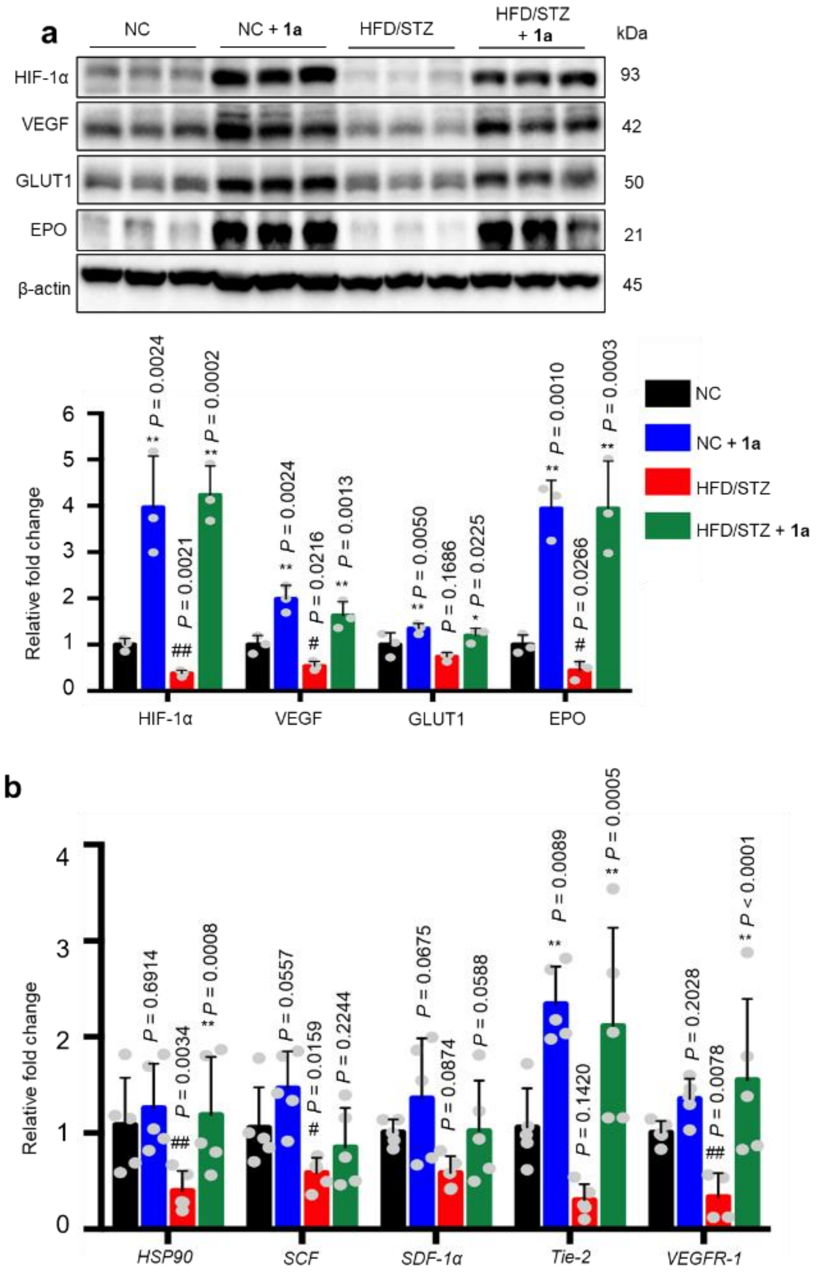

Supplementary Figure 14. Complex **1a** (1.25 mg/kg) activates the genes expression regulated by HIF-1α in HFD/STZ induced diabetic mice after 8 days post-injury. (a) Western blot analyses and quantitation of HIF-1α, VEGF, GLUT1, and EPO in wound tissue (n = 3 mice). All proteins were normalized by β-actin. (b) The mRNA levels of HIF-1α target genes involved in wound healing were analysed by qRT-PCR in wound tissues (n = 5 mice). Data are expressed as means ± SD. P values were calculated using a one-way ANOVA with Tukey's multiple comparison test. $^{\#}P < 0.05$, $^{\#\#}P < 0.01$ NC vs. HFD/STZ, $^{*}P < 0.05$, $^{**}P < 0.01$ **1a** vs. vehicle. Source data are provided as a Source Data file.

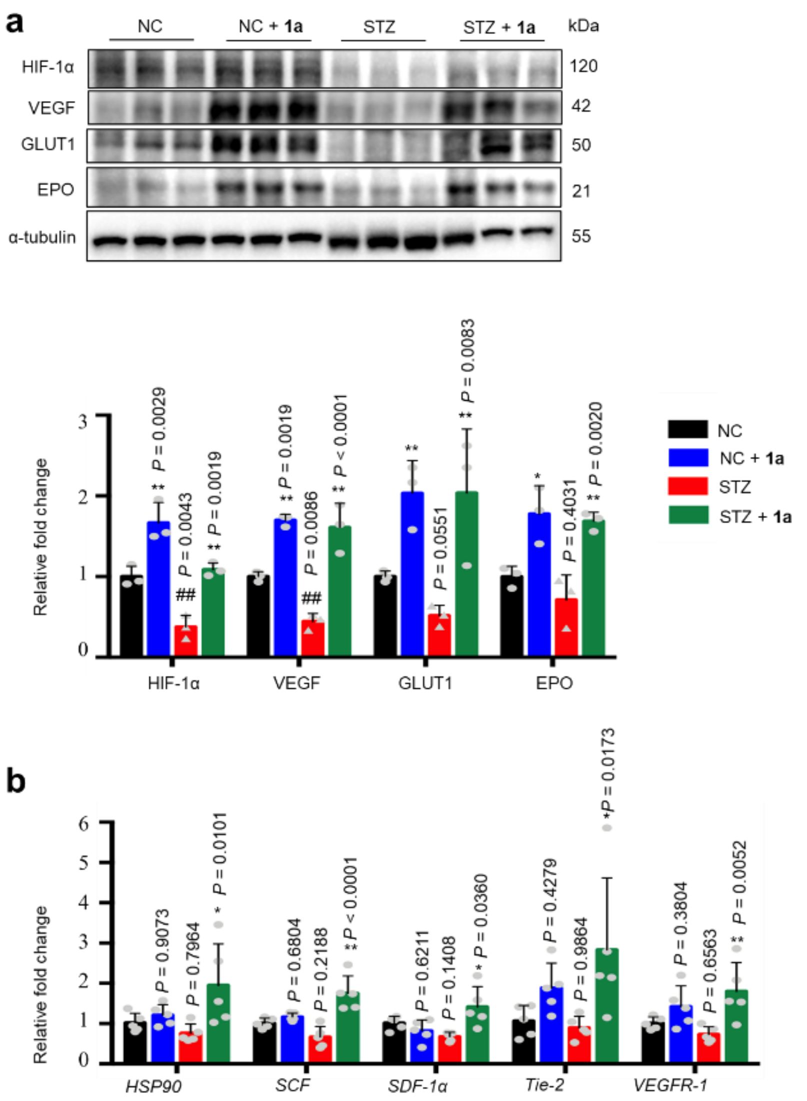

Supplementary Figure 15. Complex **1a** (1.25 mg/kg) activates the genes expression regulated by HIF-1α in STZ-induced diabetic mice after 8 days post-injury. (a) Western blot analyses and quantitation of HIF-1α, VEGF, GLUT1, and EPO in wound tissue (*n* = 3 mice). All proteins were normalized by α-tubulin. (b) The mRNA levels of HIF-1α target genes involved in wound healing were analysed by qRT-PCR in wound tissues (*n* = 5 mice). Data are expressed as means ± SD. *P* values were calculated using a one-way ANOVA with Tukey's multiple comparison test. $^{\#}P < 0.05$, $^{\#\#}P < 0.01$ NC vs. STZ, $^{*}P < 0.05$, $^{**}P < 0.01$ **1a** vs. vehicle. Source data are provided as a Source Data file.

Table S1. Antibodies used for Western blot or co-IP.

Antibody	Source	Vendor	Catalog No.
VEGF	Mouse	Novus Biologicals	NB100-664
HIF-1α	Rabbit	Novus Biologicals	NB100-479
HIF-2α	Rabbit	Novus Biologicals	NB100-122
EPO	Rabbit	abcam	ab129452
VHL	Rabbit	GeneTex	GTX101087
Elongin B	Mouse	Santa Cruz Biotechnology, Inc.	sc-133090
PHD2	Rabbit	Bethyl Laboratories, Inc.	A300-322A
PHD3	Rabbit	Bethyl Laboratories, Inc.	A300-327A
HIF-1α	Rabbit	Cell Signaling Technology	36169
CD31	Rabbit	ABclonal	A3181
α-tubulin	Mouse	Santa Cruz Biotechnology	sc-8035
β-actin	Rabbit	Cell Signaling Technology	4967L
Hydroxy-HIF-1α	Rabbit	Cell Signaling Technology	3434S
GLUT1	Rabbit	Cell Signaling Technology	12939S
COX-2	Rabbit	Cell Signaling Technology	4842S

Table S2. Real-time PCR primer sequences[11].

Gene	Forward	Reverse
18S	AGCCTGCGGCTTAATTTGAC	CAACTAAGAACGGCCATGCA
HSP-90α	GGACCAGGTTGCTAACTCCG	GGTCTTGCCCTCAAATTCCTT
VEGFR1	TGGACCCAGATGAAGTTCCC	GCGATTTGCCTAGTTTCAGTCT
SDF1α	GAGAGCCACATCGCCAGAG	TTTCGGGTCAATGCACACTTG
SCF	CCTTAGGAATGACAGCAGTAGC	AGCCAATTACAAGCGAAATGAG
Tie-2	GTGTAGTGGACCAGAAGG	CTTGAGAGCAGAGGCATC

^{1}H NMR, ^{13}C NMR and HRMS spectra of complexes **1–14**, **1a–1n**.

Complex **1**. Yield: 72%. ^{1}H NMR (400 MHz, Acetone-d_6) δ 8.43 (s, 2H), 8.35 – 8.27 (m, 4H), 8.04 (t, J = 8.2 Hz, 2H), 7.96 (d, J = 8.4 Hz, 2H), 7.73 (d, J = 5.8 Hz, 2H), 7.52 (d, J = 6.1 Hz, 2H), 7.35 (d, J = 8.3 Hz, 2H), 7.13 (t, J = 7.4 Hz, 2H), 6.51 (d, J = 2.0 Hz, 2H), 4.26 (s, 6H). ^{13}C NMR (101 MHz, Acetone) δ 170.87, 170.54, 165.11, 164.86, 153.06, 150.30, 146.81, 144.09, 139.95, 135.75, 127.51, 127.20, 125.27, 125.24, 125.01, 124.02, 121.64, 121.40, 121.38, 107.64, 57.86. MALDI-TOF-HRMS: Calcd. for $C_{36}H_{26}Br_2N_4O_2Rh$ $[M–PF_6]^+$: 808.9457 Found: 808.9435. Anal.: ($C_{36}H_{26}Br_2N_4O_2RhPF_6$) C, H, N: calcd. 45.31, 2.75, 5.87; found 45.13, 2.72, 5.76.

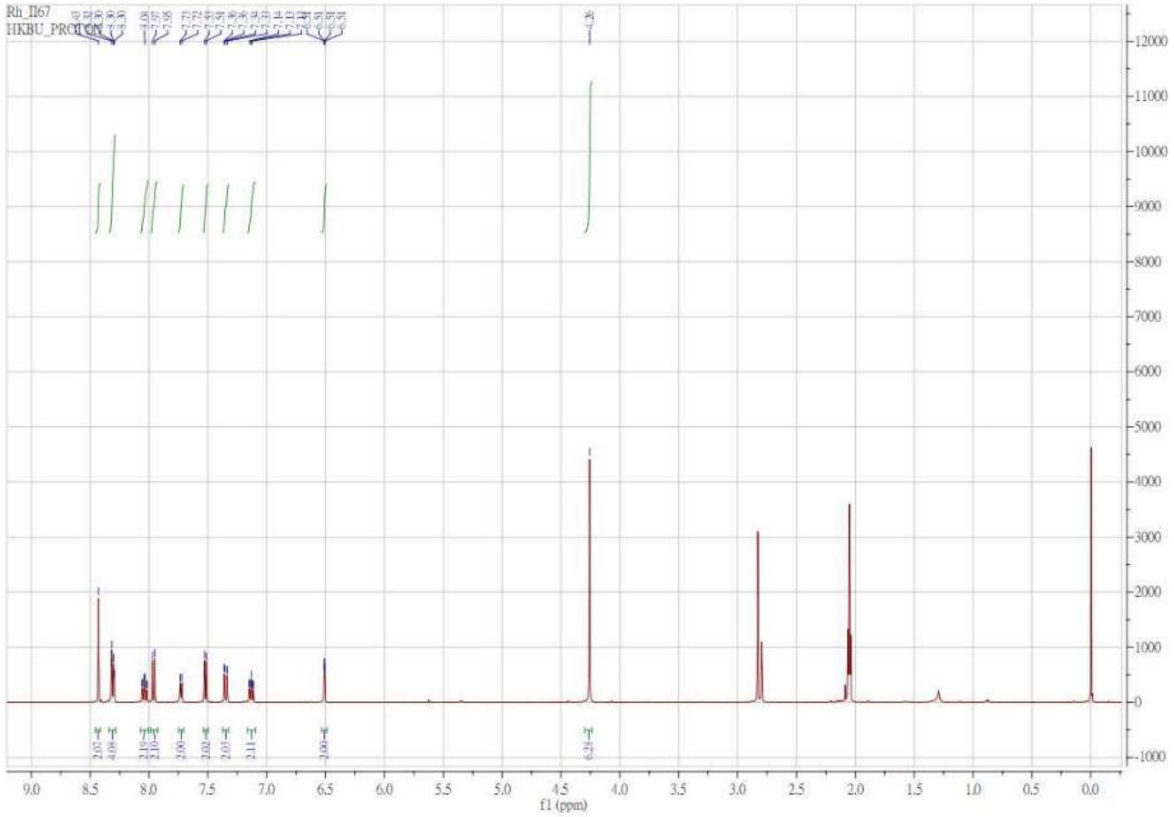

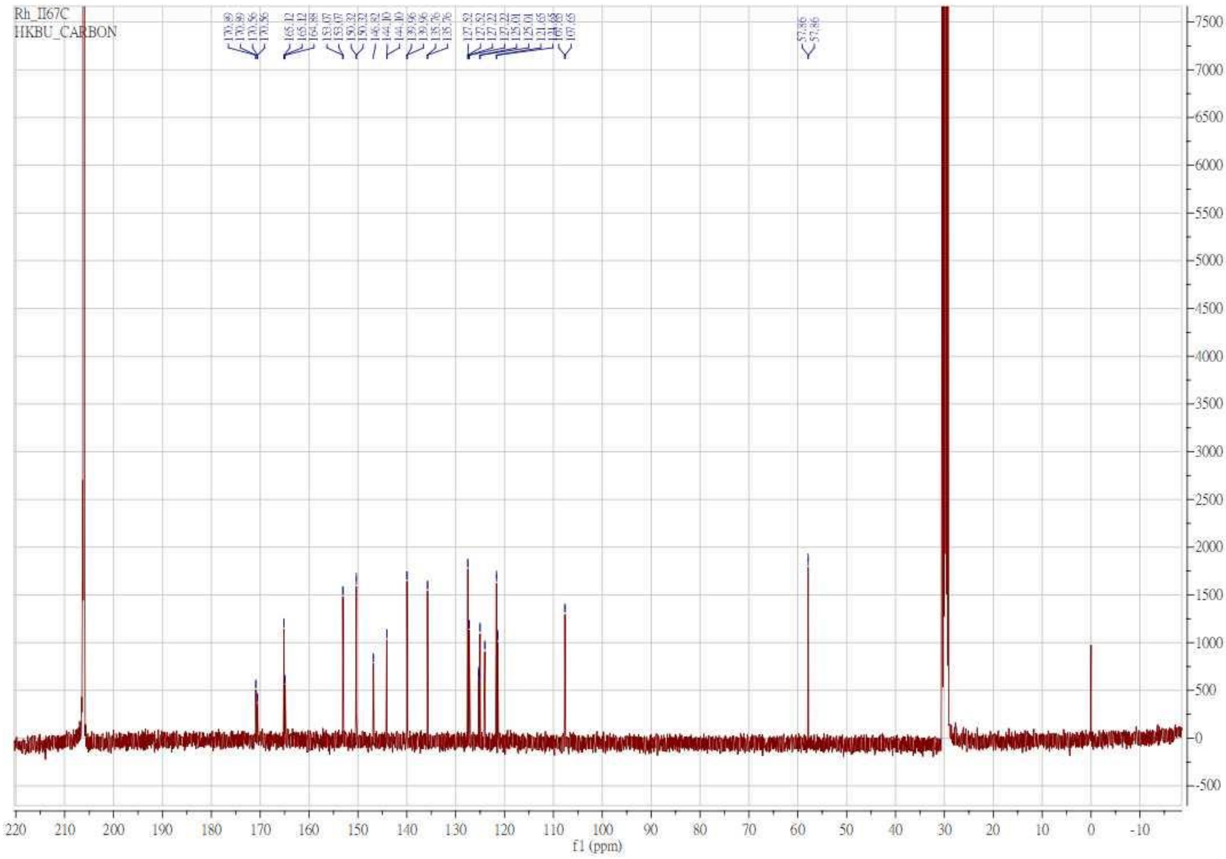

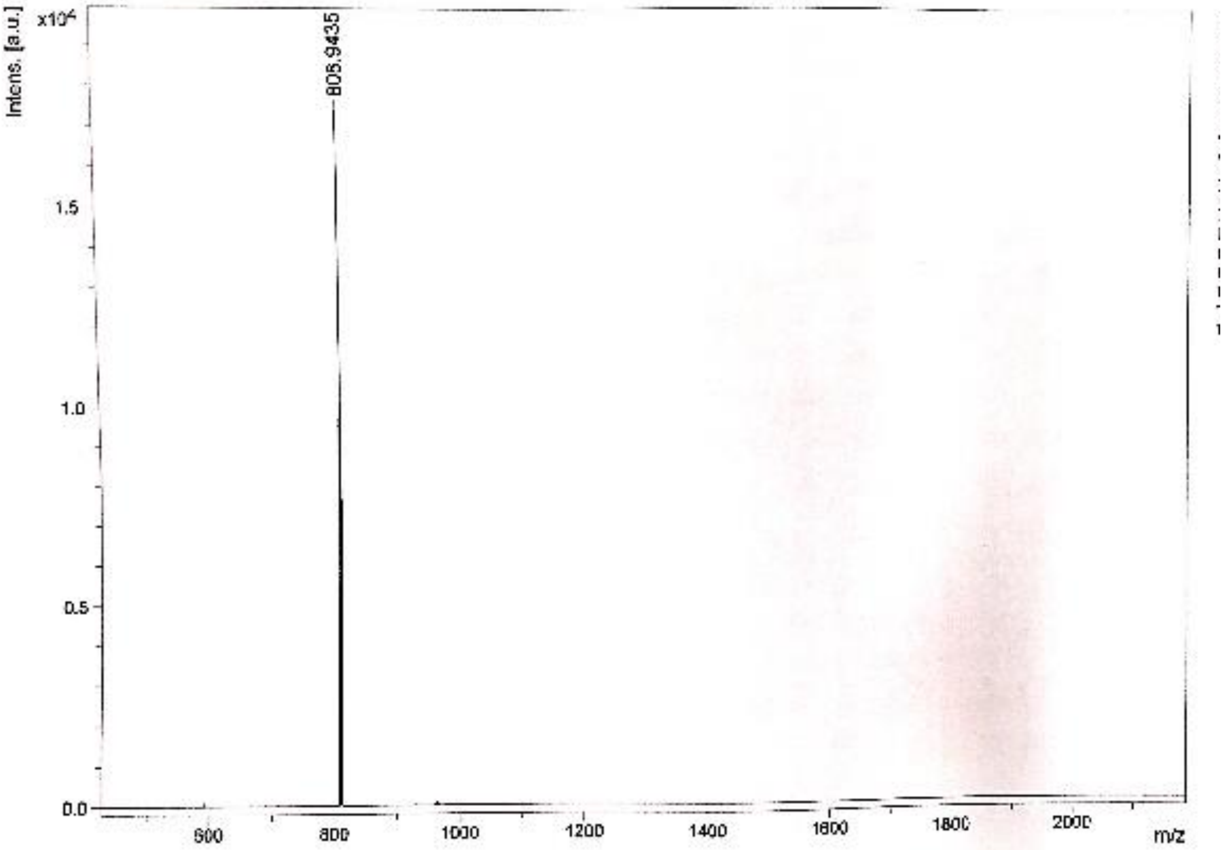

Complex **2**. Yield: 81%. ^{1}H NMR (400 MHz, Acetone-d_6) δ 9.16 (d, J = 13.8 Hz, 2H),

8.65 (dd, J = 8.1, 1.4 Hz, 2H), 8.34 – 8.24 (m, 4H), 8.01 (d, J = 8.8 Hz, 2H), 7.96 – 7.85 (m, 4H), 7.66 – 7.58 (m, 4H), 7.24 (t, J = 7.6 Hz, 2H), 6.36 (d, J = 7.2 Hz, 2H), 4.30 – 4.06 (m, 8H), 1.33 – 1.27 (m, 12H). ^{13}C NMR (101 MHz, $CDCl_3$) δ 164.55, 164.22, 155.88, 155.75, 155.12, 152.10, 151.97, 149.96, 143.85, 142.01, 140.50, 138.32, 134.96, 131.04, 130.49, 130.43, 130.34, 128.37, 127.17, 127.06, 124.88, 123.64, 122.60, 63.95, 63.93, 63.9016.66, 16.64, 16.61, 16.58. MALDI-TOF-HRMS: Calcd. for $C_{44}H_{42}N_4O_6P_2Rh$ $[M–PF_6]^+$: 887.1635 Found: 886.1672. Anal.: ($C_{44}H_{42}N_4O_6P_2RhPF_6$) C, H, N: calcd. , 51.18, 4.10, 5.43; found 50.88, 4.20, 5.45.

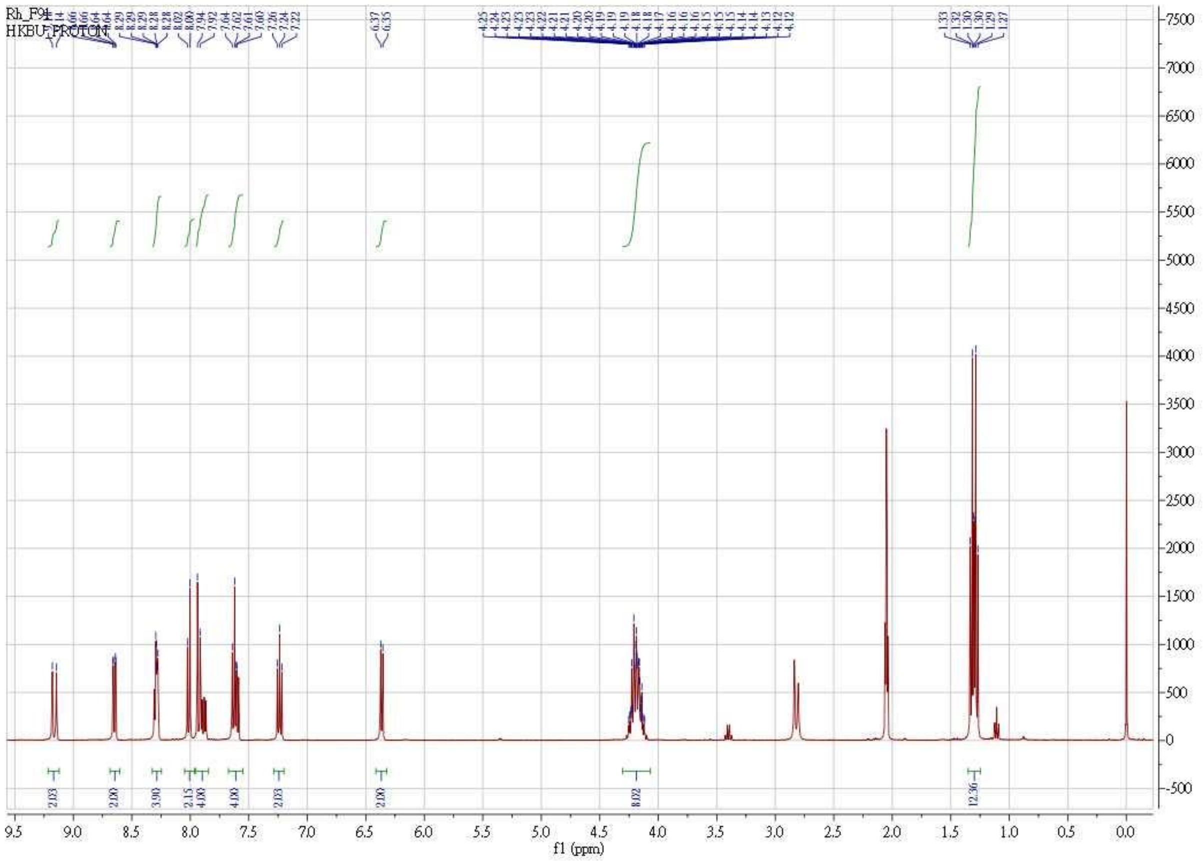

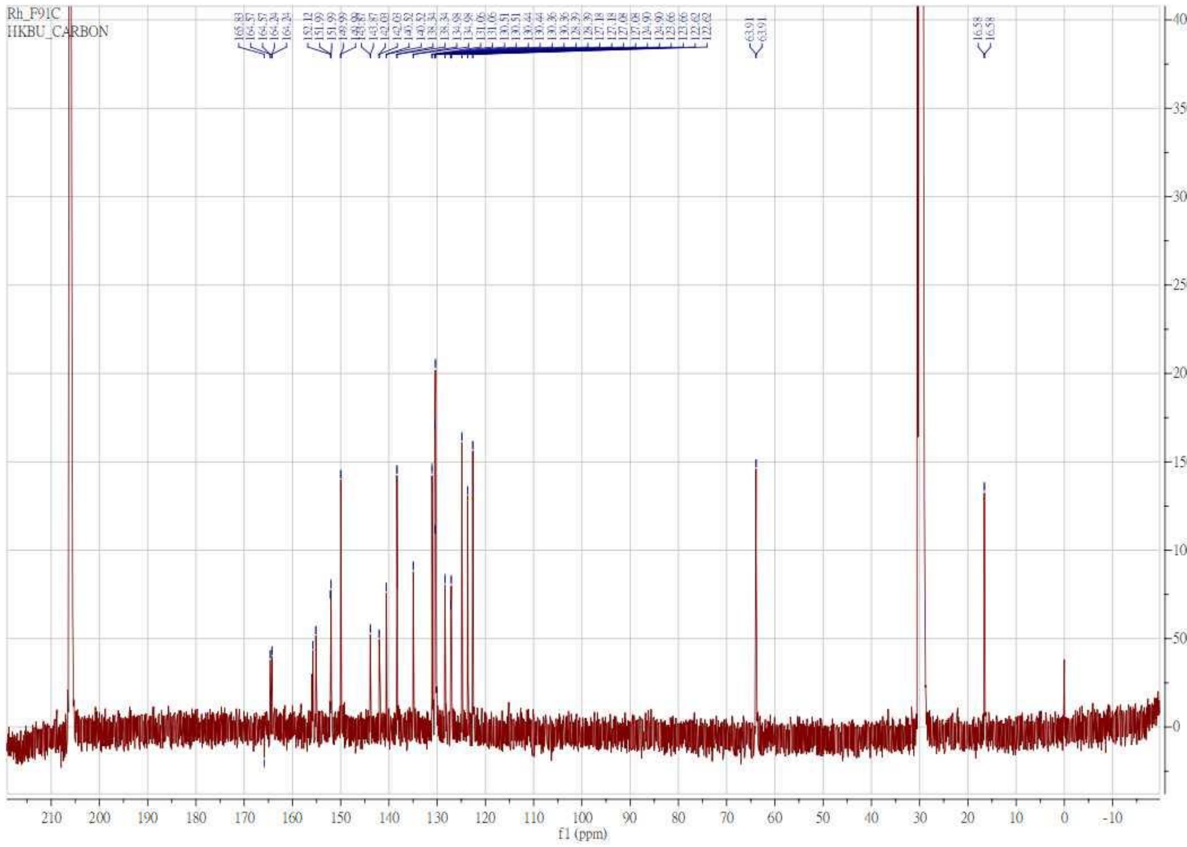

Rh_F91C
HKBU_CARBON
63.91
63.91
16.58
16.58
f1 (ppm)

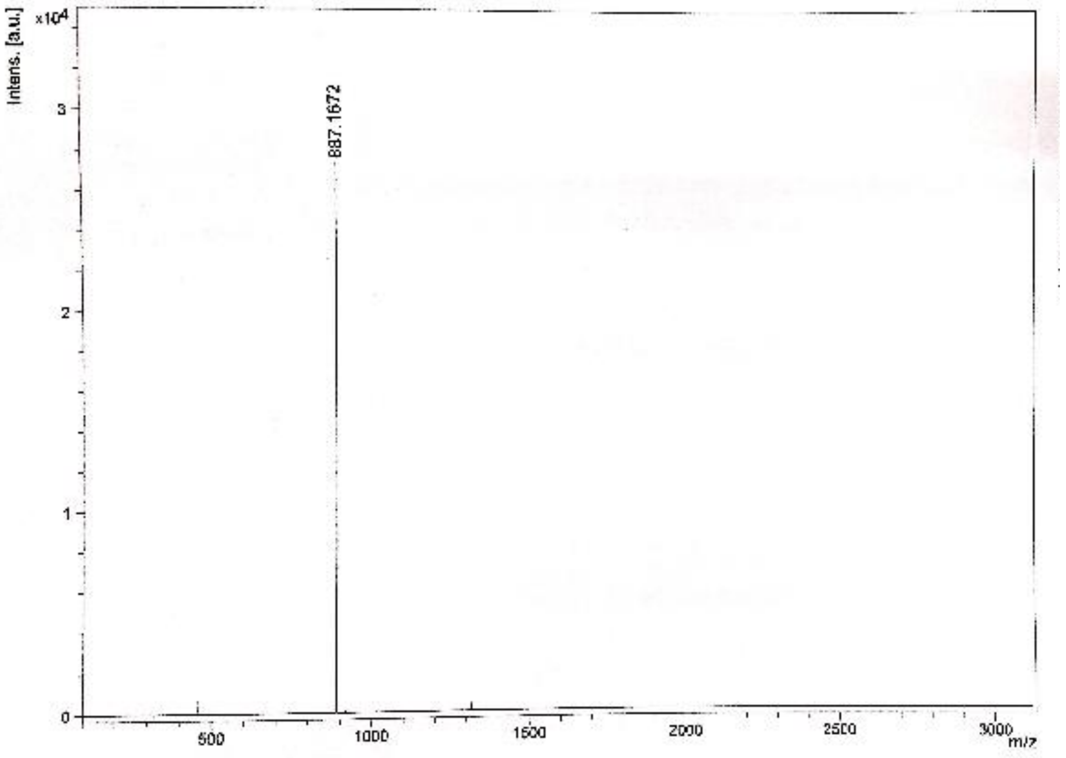

Intens. [a.u.]
x10^4
887.1672
m/z

Complex **3**. Yield: 68%. ^{1}H NMR (400 MHz, Acetone-d_6) δ 8.82 (d, J = 8.2 Hz, 2H), 8.32 (t, J = 7.9 Hz, 2H), 8.22 – 8.17 (m, 2H), 8.11 (t, J = 8.2 Hz, 2H), 7.99 – 7.93 (m, 2H), 7.91 (d, J = 8.6 Hz, 2H), 7.74 – 7.70 (m, 4H), 7.10 (t, J = 7.3 Hz, 2H), 6.70 (dd, J = 8.6, 2.5 Hz, 2H), 5.86 (dd, J = 2.5, 1.1 Hz, 2H), 3.59 (s, 6H). ^{13}C NMR (101 MHz, Acetone) δ 170.59, 170.27, 165.83, 165.82, 161.58, 161.56, 155.57, 151.16, 149.78, 140.78, 139.39, 137.54, 128.86, 126.97, 125.01, 123.19, 120.18, 120.17, 118.94, 109.39, 55.07. MALDI-TOF-HRMS: Calcd. for $C_{34}H_{28}N_4O_2Rh$ $[M–PF_6]^+$: 627.1267 Found: 627.1266. Anal.: ($C_{34}H_{28}N_4O_2RhPF_6$) C, H, N: calcd. 52.86, 3.65, 7.25; found 52.62, 3.66, 7.36.

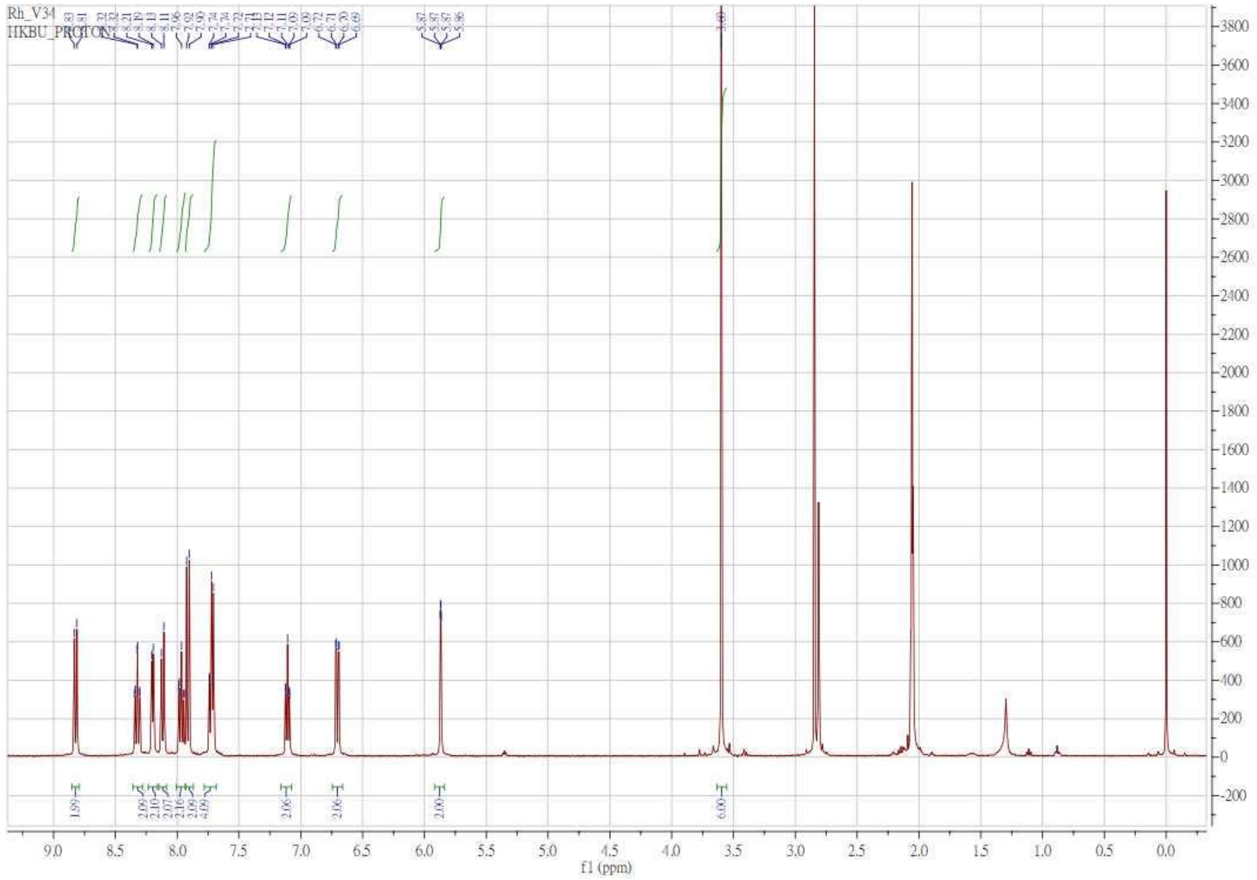

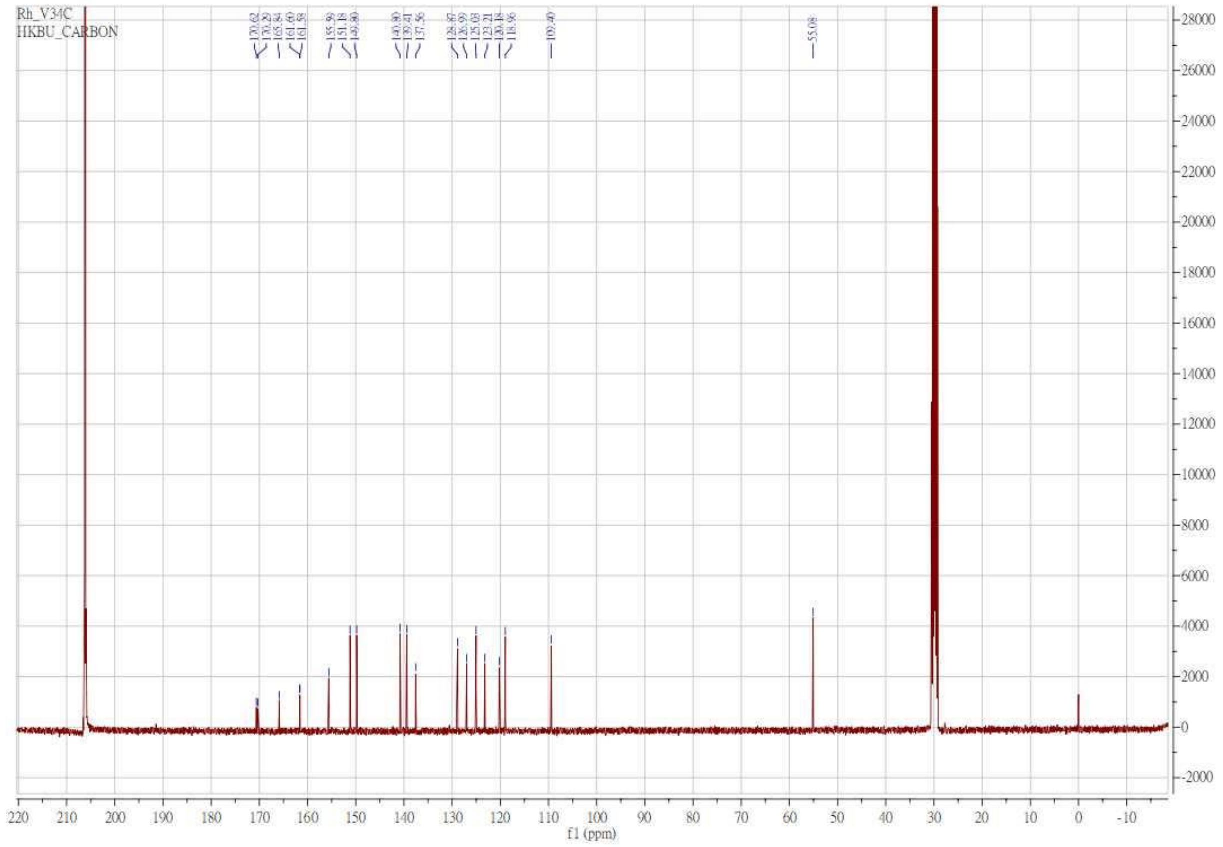
Rh_V34C
HKBU_CARBON
55.08
f1 (ppm)

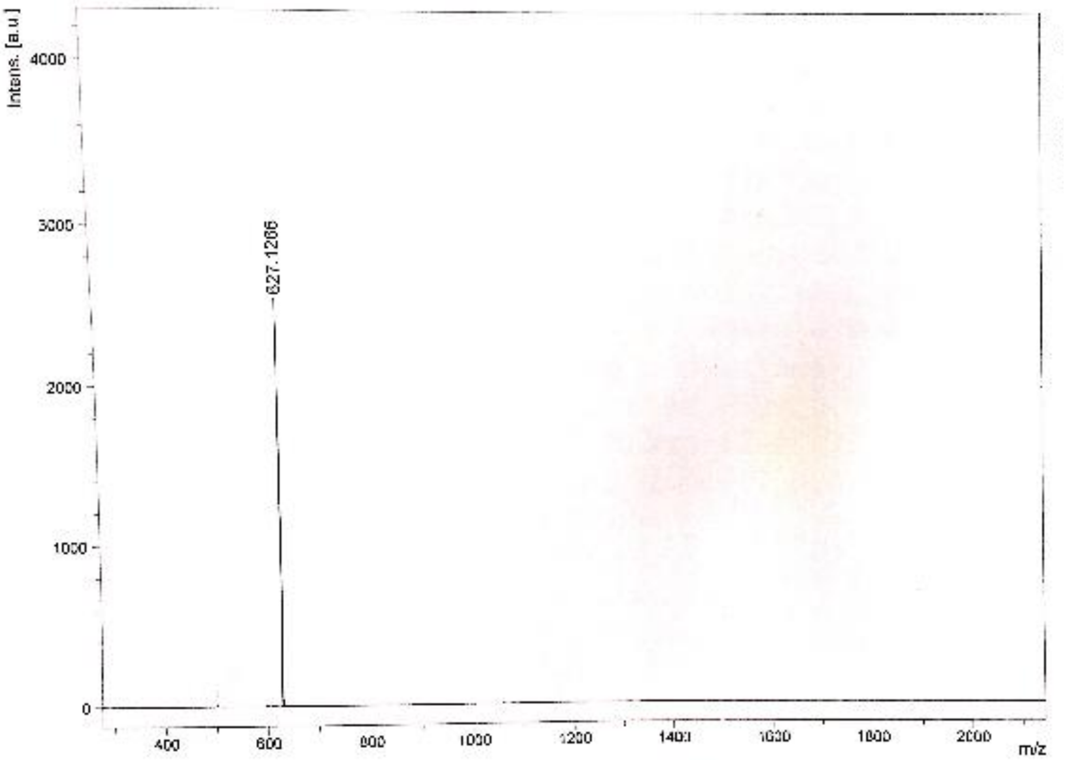
Intens. [a.u.]
627.1266
m/z

Complex **4**. Yield: 60%. ^{1}H NMR (400 MHz, Acetone-d_6) δ 8.53 (s, 2H), 8.27 (d, J = 5.3 Hz, 2H), 8.23 (dt, J = 8.3, 1.3 Hz, 2H), 7.95–7.85 (m, 6H), 7.67 (ddd, J = 5.8, 1.6, 0.8 Hz, 2H), 7.06 (td, J = 7.5, 1.2 Hz, 2H), 7.01 – 6.91 (m, 4H), 6.53 – 6.38 (m, 2H), 3.01 (d, J = 0.9 Hz, 6H). ^{13}C NMR (101 MHz, Acetone) δ168.99, 151.76, 151.60, 150.23, 148.57, 147.41, 142.83, 139.32, 136.75, 131.89, 130.74, 130.64, 130.34, 130.01, 128.08, 127.14, 125.87, 123.75, 123.26, 120.39, 15.52. MALDI-TOF-HRMS: Calcd. for $C_{36}H_{28}N_4Ir$ $[M–PF_6]^+$: 709.1943 Found: 709.9267. Anal.: ($C_{36}H_{28}F_6N_4PIr+0.5H_2O$) C, H, N: calcd. 50.11, 3.39, 6.49; found 56.02, 3.29, 6.62.

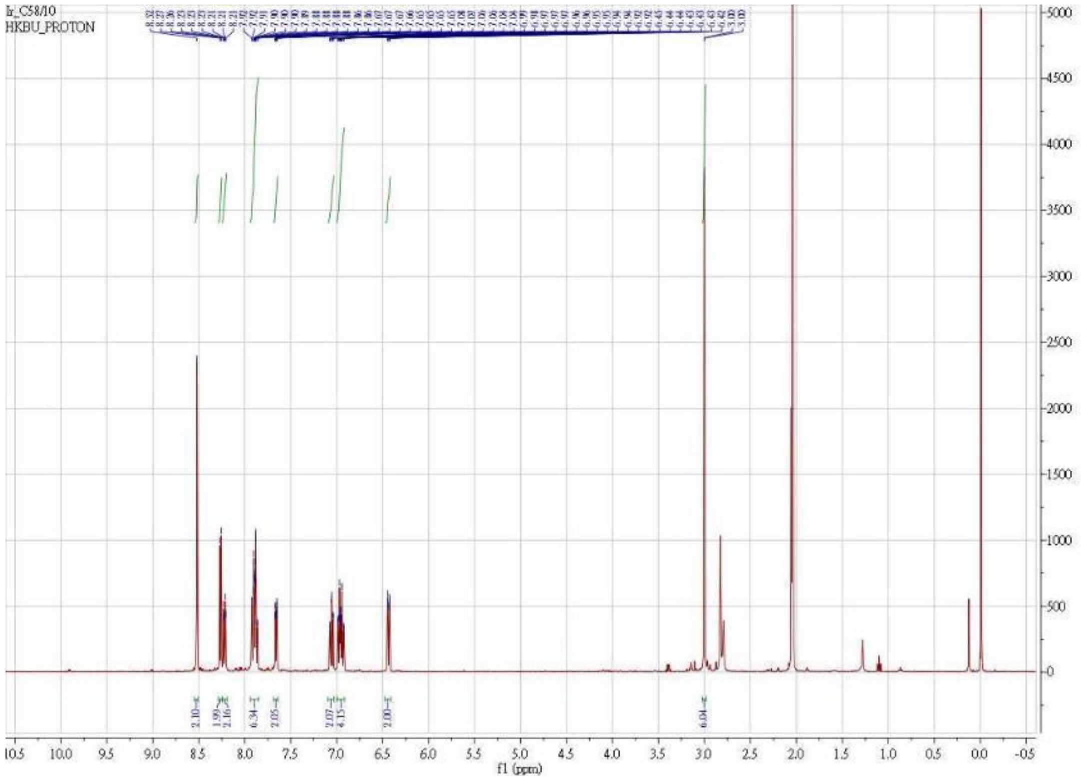

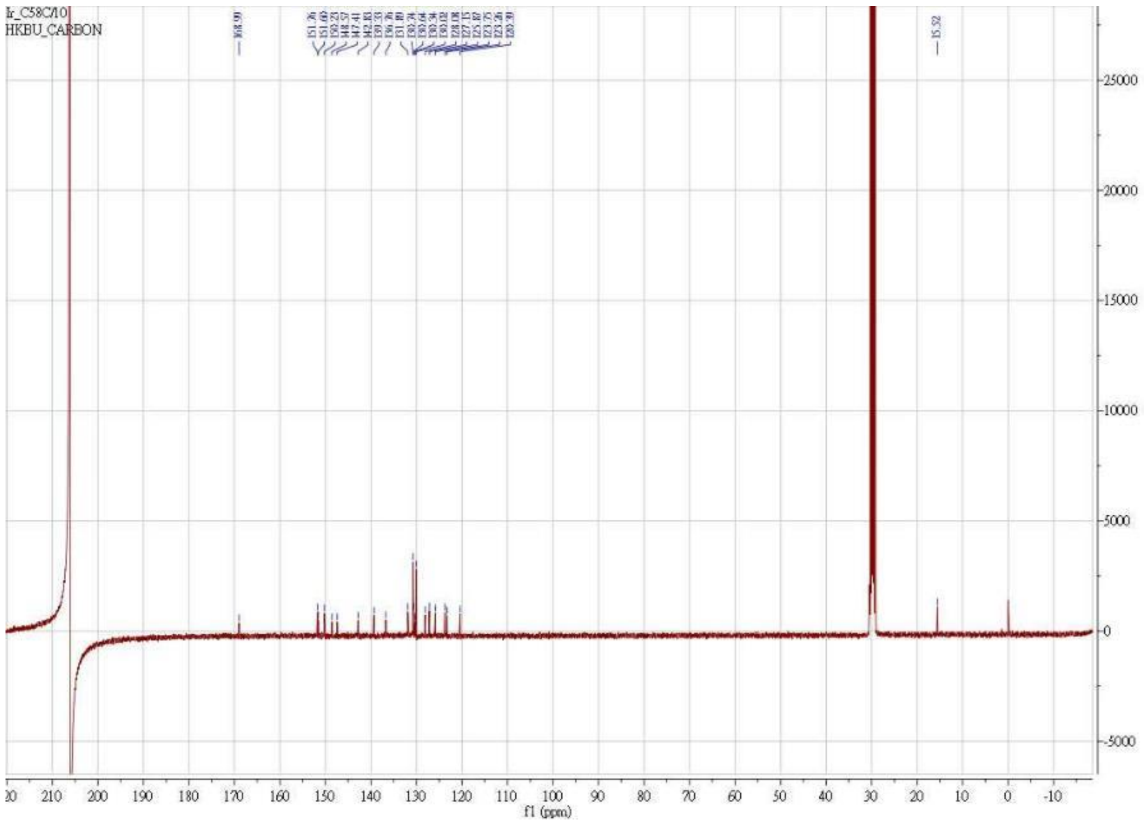

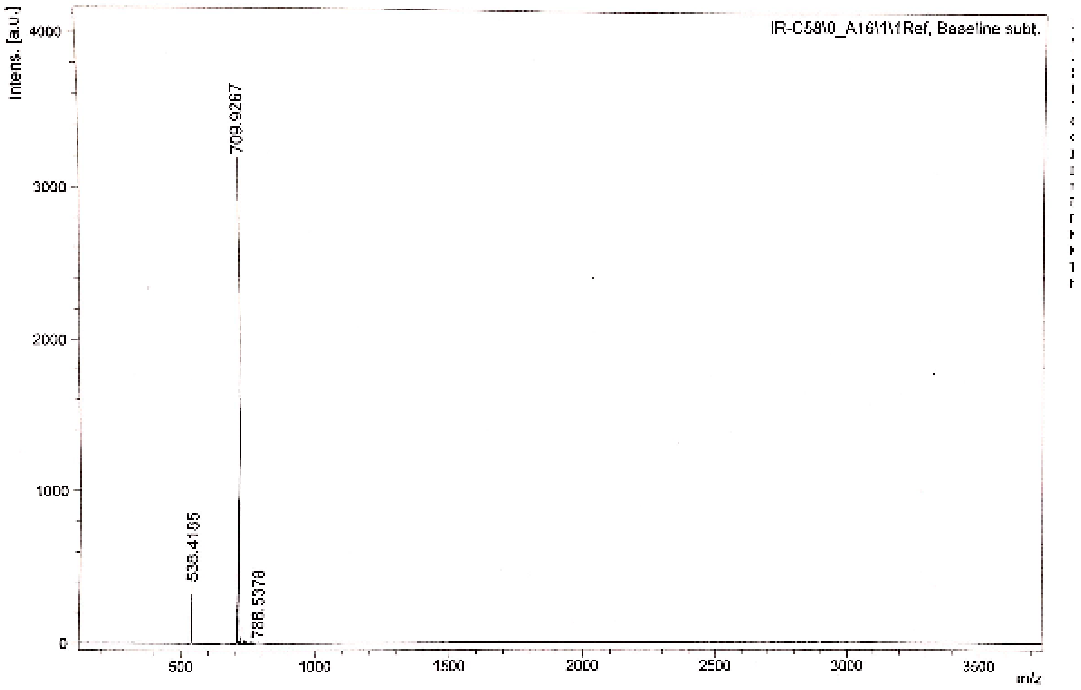

Complex **5**. Yield: 68%. ^{1}H NMR (400 MHz, Acetone-d_6) δ 8.55 (d, J = 5.2 Hz, 2H),

8.27 (s, 2H), 8.23 (d, *J* = 8.3 Hz, 2H), 8.03 – 7.95 (m, 4H), 7.92 (d, *J* = 8.0 Hz, 2H), 7.76 (d, *J* = 5.8 Hz, 2H), 7.70 – 7.61 (m, 10H), 7.07 – 6.98 (m, 4H), 6.35 (s, 2H), 2.44 (q, *J* = 7.6 Hz, 4H), 1.05 (t, *J* = 7.6 Hz, 6H). ^{13}C NMR (101 MHz, Acetone) δ 168.92, 168.60, 166.20, 166.19, 151.74, 151.10, 150.22, 147.15, 147.11, 142.66, 139.42, 136.92, 132.86, 130.63, 130.51, 129.95, 129.67, 127.66, 126.73, 125.53, 124.13, 123.80, 120.55, 15.43. MALDI-TOF-HRMS: Calcd. for $C_{50}H_{40}N_4Rh$ $[M–PF_6]^+$: 799.2308 Found: 799.2307. Anal.: ($C_{50}H_{40}N_4RhPF_6$) C, H, N: calcd. 63.57, 4.27, 5.93; found 63.54, 4.34, 6.02.

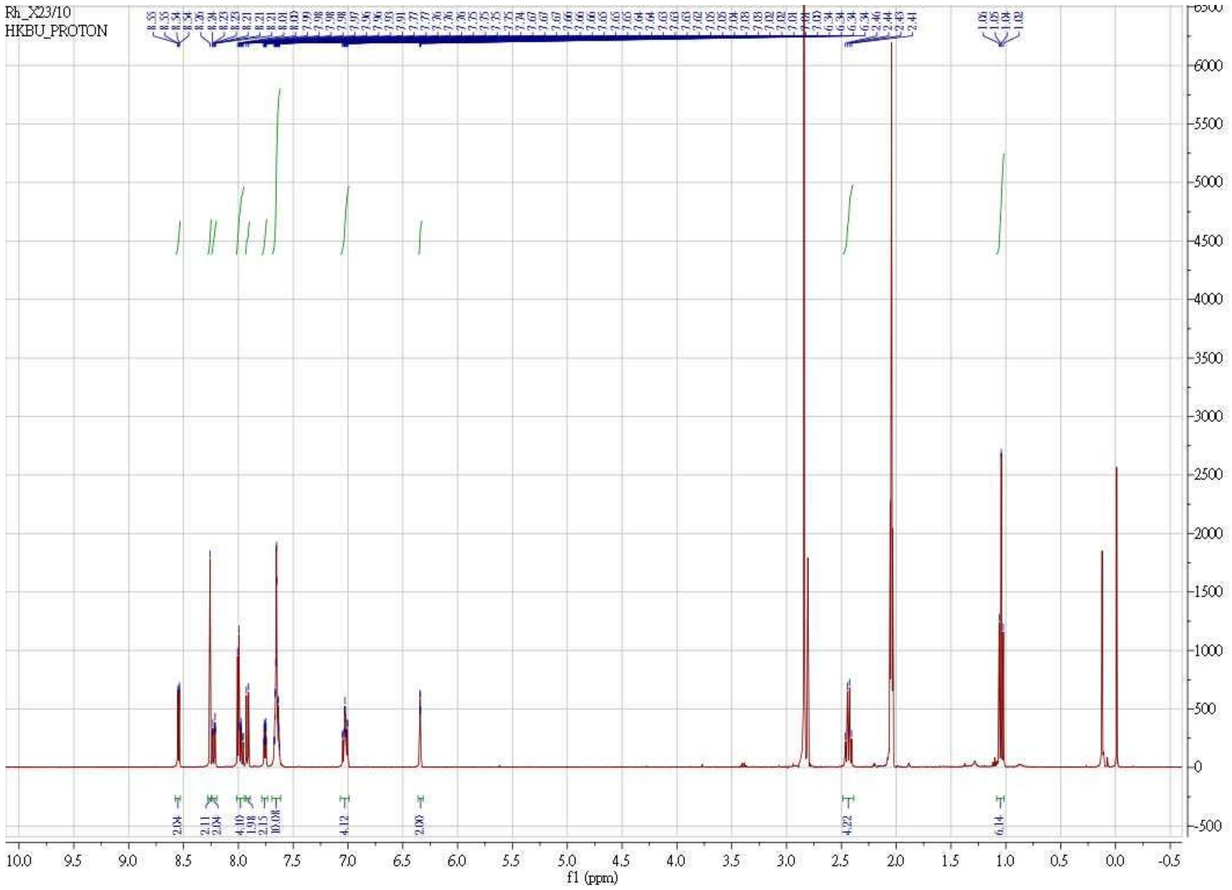

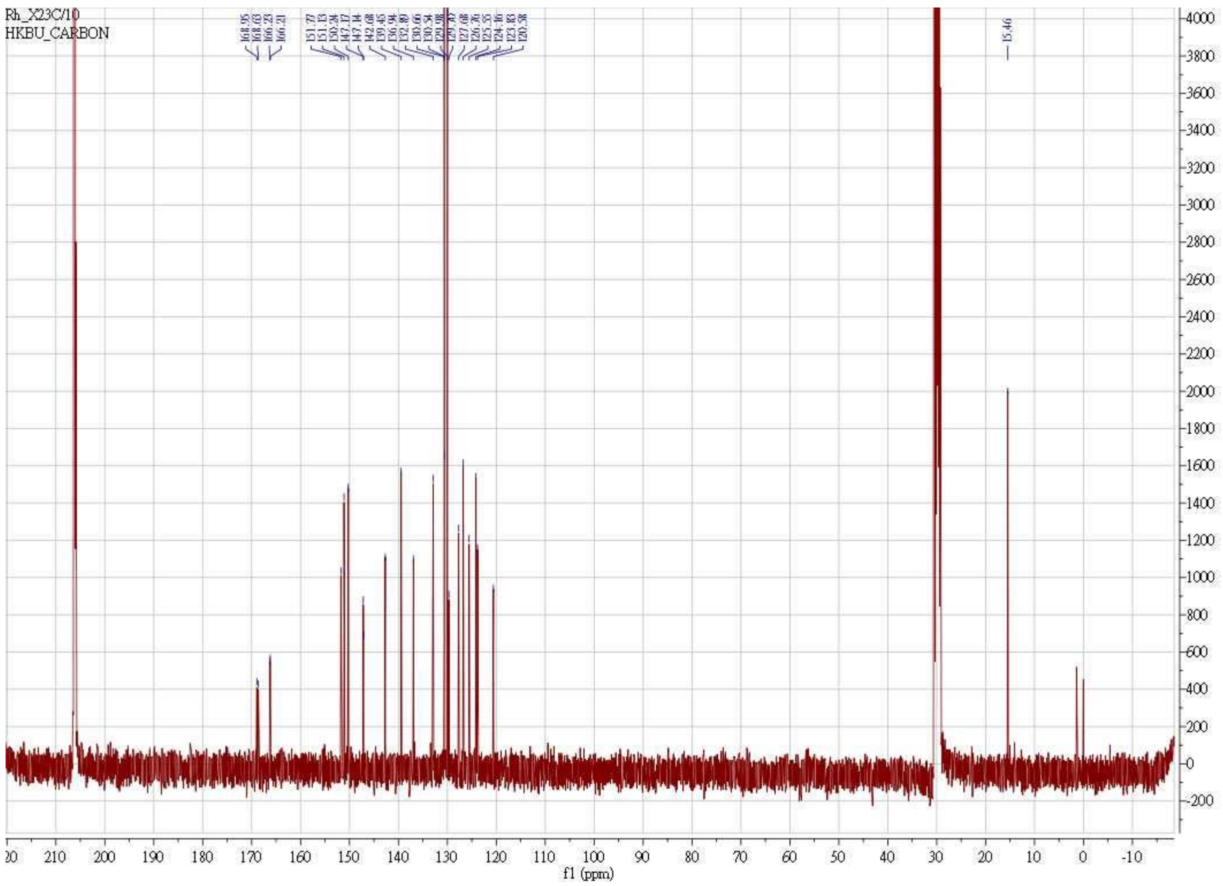

Rh_X23C/10
HKBU_CARBON
168.95
168.63
166.23
166.21
151.77
151.13
150.24
147.17
147.14
142.08
139.45
136.94
132.89
130.66
130.54
127.68
126.76
125.55
124.16
123.83
120.38
15.46
f1 (ppm)

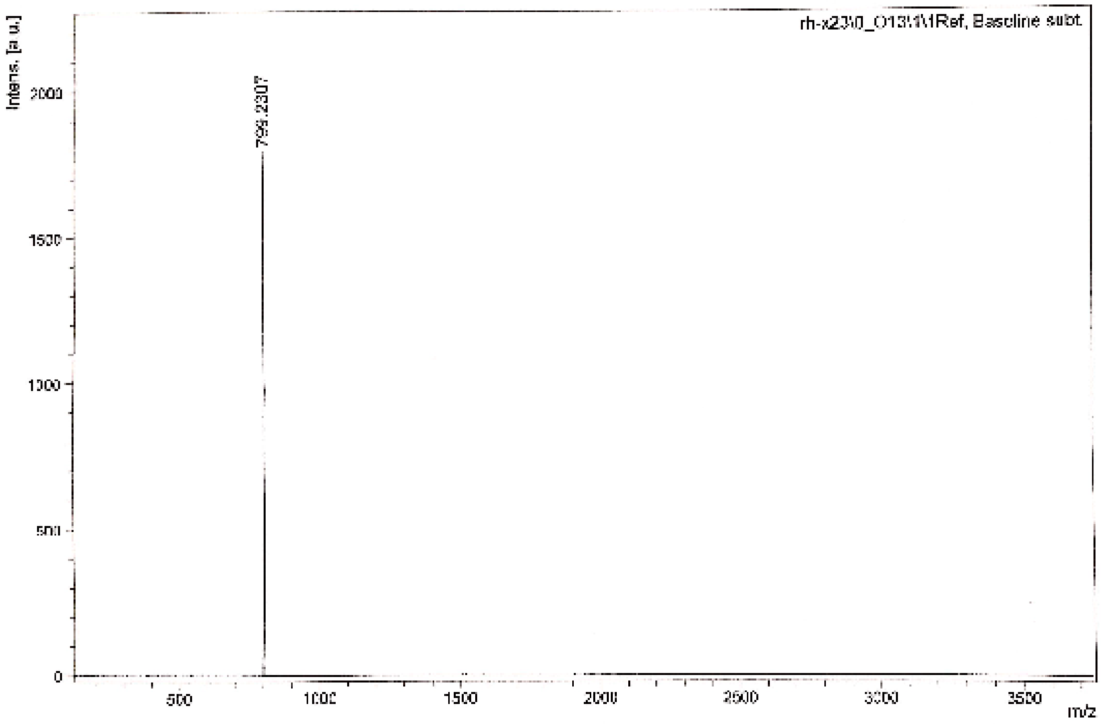

rh-x23\0_O13\1\1Ref, Baseline subt.
Intens. [a.u.]
799.2307
m/z

Complex **6**. Yield: 31%. ^{1}H NMR (400 MHz, Acetone-d_6) δ 8.56 (d, J = 8.7 Hz, 2H), 8.46 (d, J = 8.9 Hz, 2H), 8.20 (d, J = 8.0 Hz, 2H), 8.14 (d, J = 6.3 Hz, 2H), 7.93 (d, J = 8.1 Hz, 2H), 7.86 (d, J = 2.6 Hz, 2H), 7.62 (d, J = 8.9 Hz, 2H), 7.44 (t, J = 8.0 Hz, 2H), 7.24 – 7.15 (m, 4H), 7.09 (d, J = 8.0 Hz, 2H), 6.44 (d, J = 1.3 Hz, 2H), 3.94 (s, 6H), 2.25 (q, J = 7.6 Hz, 4H), 0.80 (t, J = 7.6 Hz, 6H). ^{13}C NMR (101 MHz, Acetone) δ169.94, 168.87, 167.25, 156.87, 149.93, 147.81, 147.17, 144.17, 140.37, 135.44, 131.14, 129.83, 128.89, 127.77, 127.35, 126.04, 124.47, 118.83, 114.30, 110.69, 56.96, 15.27. MALDI-TOF-HRMS: Calcd. for $C_{46}H_{40}N_4O_2Rh$ [M–PF_6]$^+$: 783.2209 Found: 783.2269. Anal.: ($C_{46}H_{40}N_4O_2RhPF_6$ + 3H2O) C, H, N: calcd. 56.22, 4.72, 5.70; found 55.95, 4.29, 5.61.

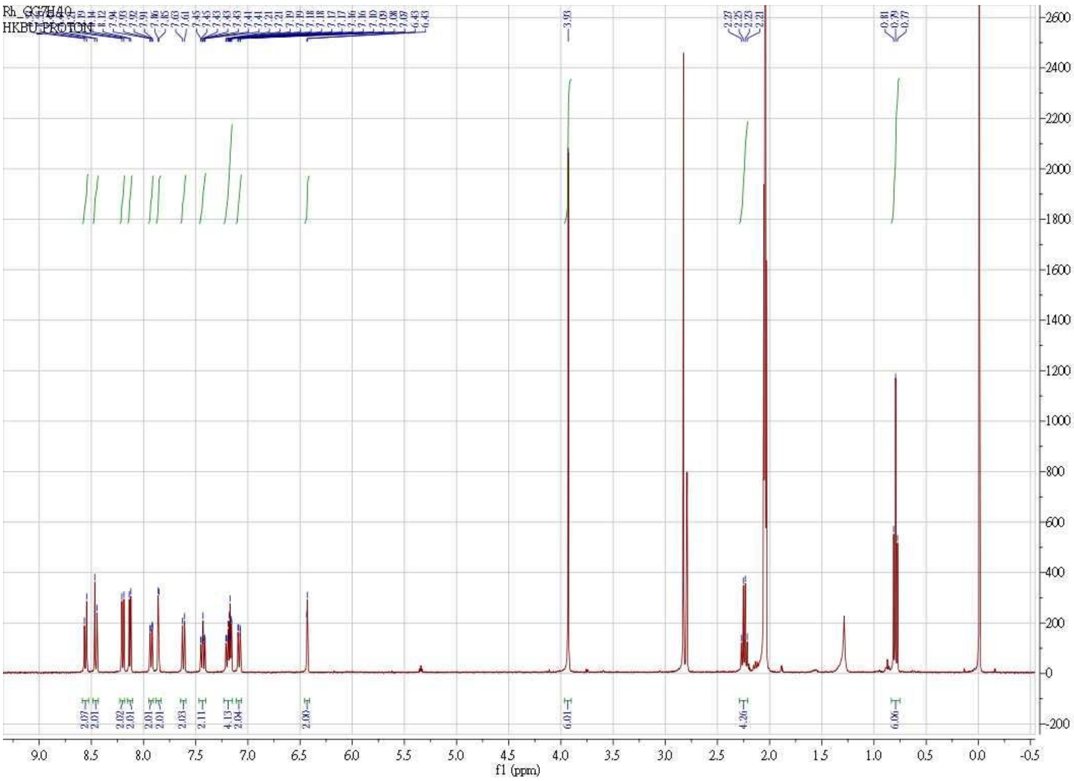

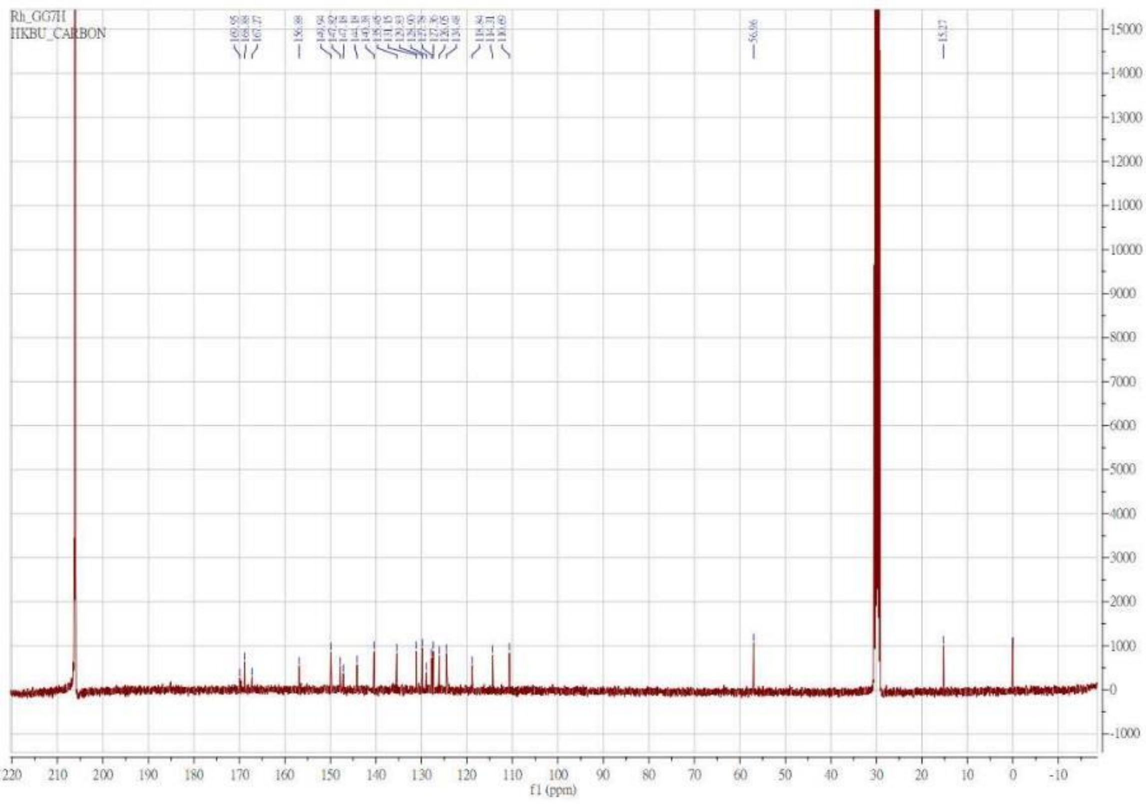
Rh_GG7H
HKBU_CARBON
56.06
15.27
f1 (ppm)

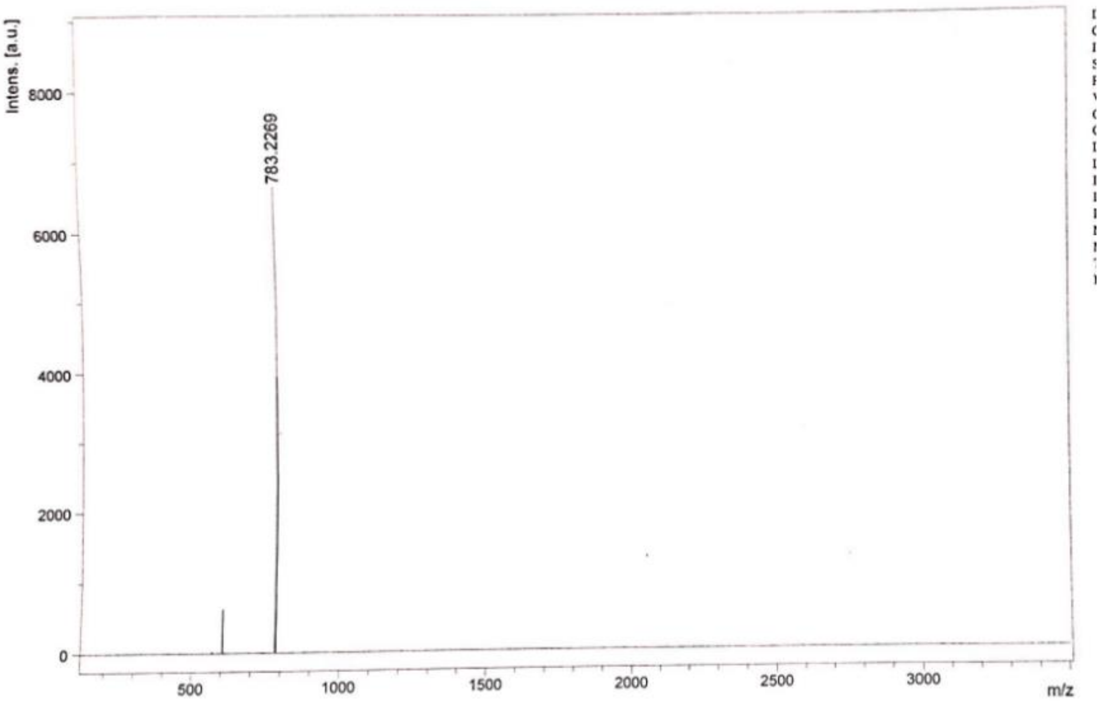
Intens. [a.u.]
783.2269
m/z

Complex **7**. Yield: 35%. ^{1}H NMR (400 MHz, Acetone-*d*$_6$) δ 8.86 (d, *J* = 1.7 Hz, 2H), 8.68 (t, *J* = 5.5 Hz, 2H), 8.65 – 8.51 (m, 4H), 8.34 (d, *J* = 7.9 Hz, 2H), 8.10 (d, *J* = 5.5 Hz, 2H), 7.93 (d, *J* = 8.1 Hz, 2H), 7.52 (q, *J* = 8.9 Hz, 2H), 7.43 (dd, *J* = 8.0, 6.9 Hz, 2H), 7.34 – 7.23 (m, 2H), 7.18 (dd, *J* = 8.7, 6.9 Hz, 2H), 6.94 (dd, *J* = 7.8, 7.2 Hz, 2H), 6.60 (t, *J* = 7.9 Hz, 2H), 3.93 (s, 6H). ^{13}C NMR (101 MHz, Acetone) δ 168.10, 167.80, 167.25, 164.40, 155.69, 150.41, 147.50, 146.50, 141.47, 141.04, 136.00, 131.83, 131.31, 131.29, 130.11, 129.13, 128.24, 127.88, 127.83, 125.70, 125.08, 124.12, 119.30, 53.68. MALDI-TOF-HRMS: Calcd. for $C_{44}H_{32}N_4O_4Rh$ $[M–PF_6]^+$: 783.1479 Found: 783.1495. Anal.: ($C_{44}H_{32}N_4O_4RhPF_6 + 2H_2O$) C, H, N: calcd. , 54.78, 3.76, 5.81; found 54.65, 3.48, 5.71.

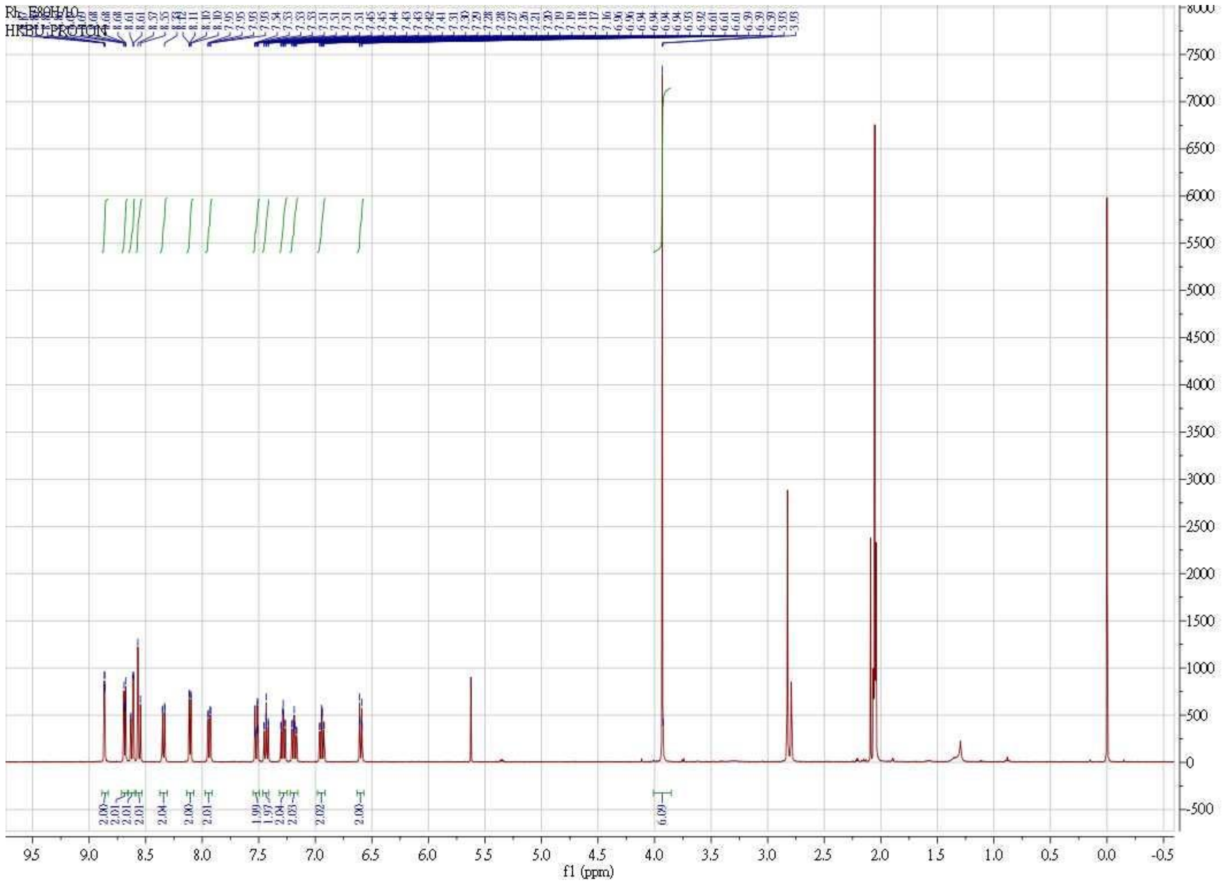

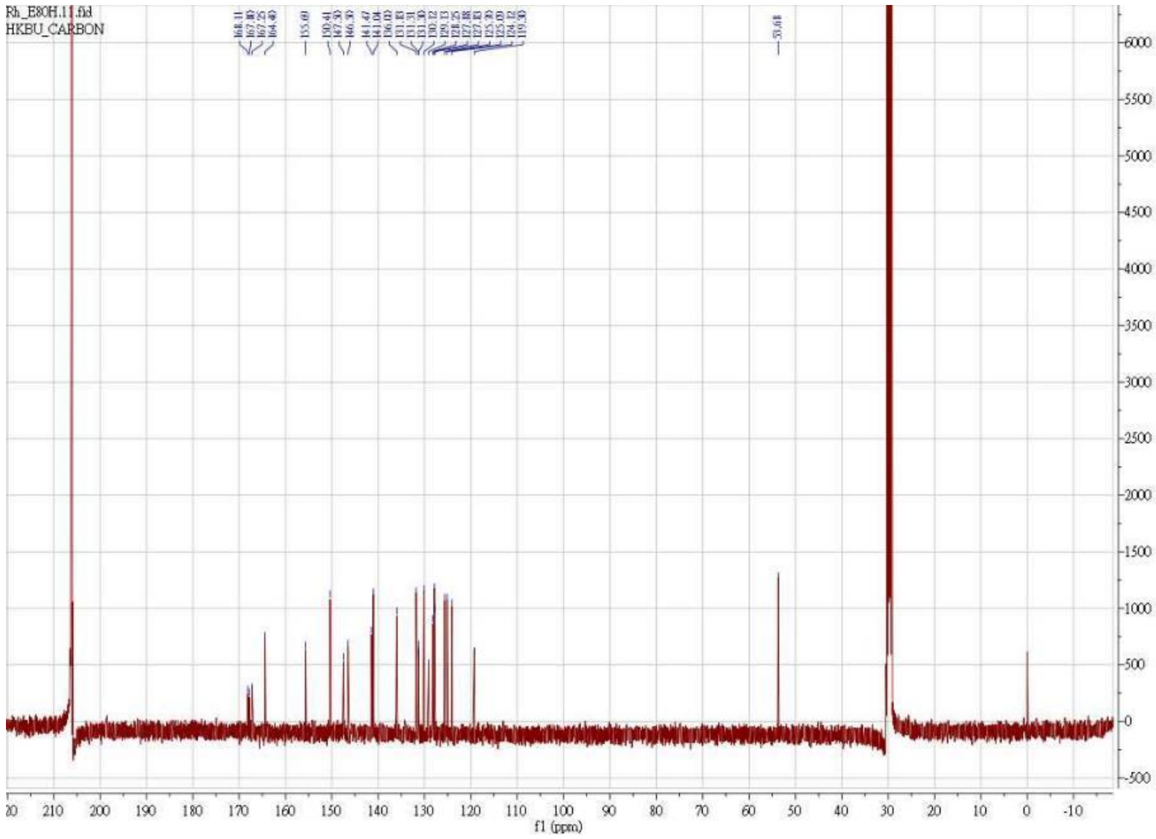
Rh_E8OH.11.fid
HKBU_CARBON
f1 (ppm)

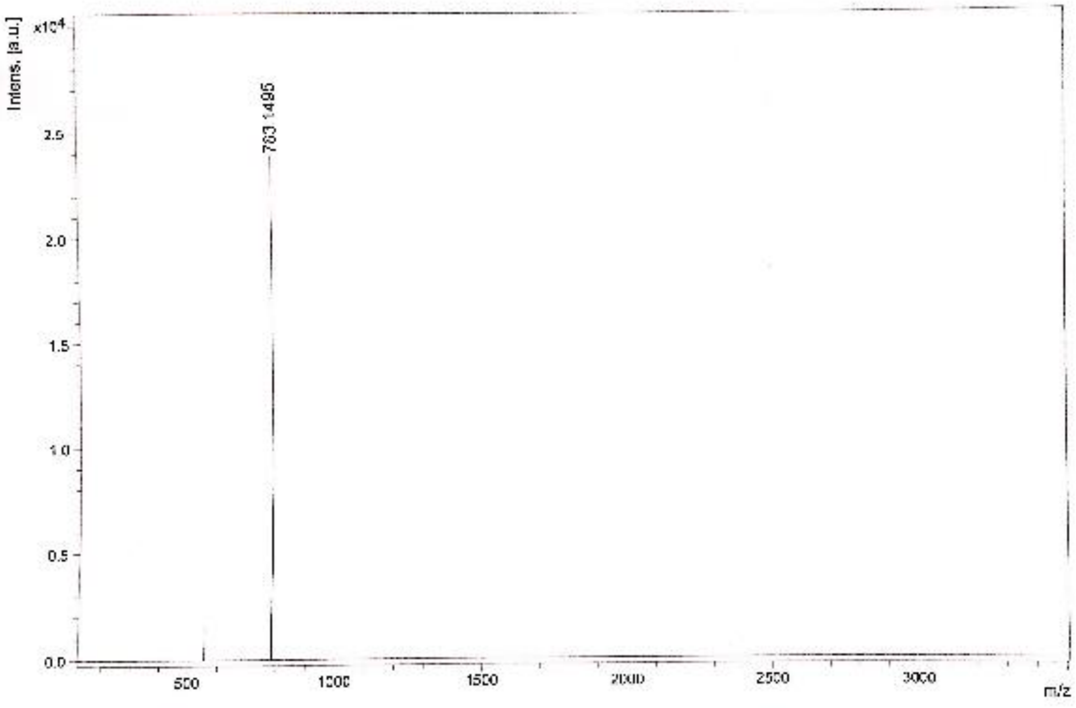
Intens. [a.u.]
x10^4
783 1495
m/z

Complex **8**. Yield: 71%. ^{1}H NMR (400 MHz, Acetone-d_6) δ 8.51 (d, J = 8.0 Hz, 2H), 8.13 (d, J = 7.2 Hz, 2H), 8.09 (d, J = 7.9 Hz, 2H), 8.00 (d, J = 7.8 Hz, 2H), 7.84 (d, J = 7.6 Hz, 2H), 7.47 (d, J = 7.6 Hz, 2H), 7.10 (dd, J = 7.7, 5.8 Hz, 2H), 6.95 (t, J = 7.2 Hz, 2H), 6.72 (t, J = 7.5 Hz, 2H), 6.19 (dd, J = 7.8, 1.4 Hz, 2H), 2.87 (s, 6H), 1.87 (s, 6H). ^{13}C NMR (101 MHz, Acetone) δ 164.99, 163.07, 157.88, 155.18, 150.57, 148.82, 145.18, 142.84, 140.18, 137.80, 132.78, 131.41, 128.77, 123.82, 123.12, 122.02, 117.96, 25.85, 24.73. MALDI-TOF-HRMS: Calcd. for $C_{36}H_{32}IrN_4$ $[M–PF_6]^+$: 713.2256 Found: 713.2238. Anal.: ($C_{36}H_{32}IrN_4PF_6$) C, H, N: calcd. 48.37, 4.06, 6.27; found 48.17, 3.64, 6.29.

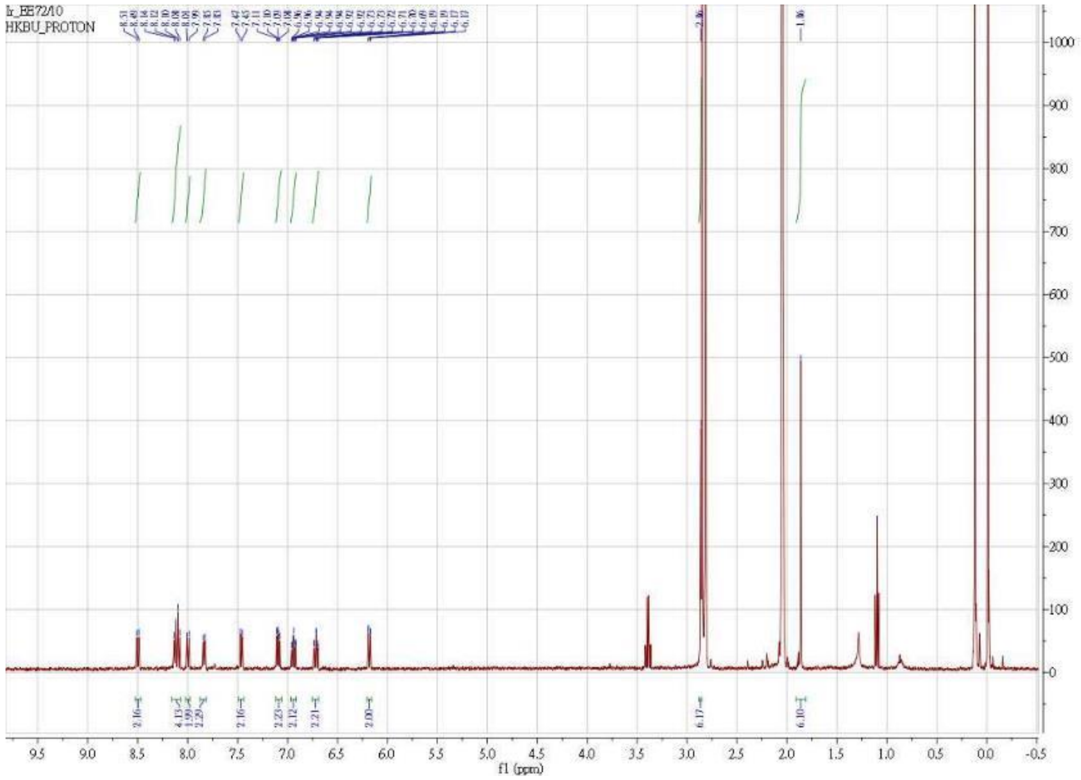

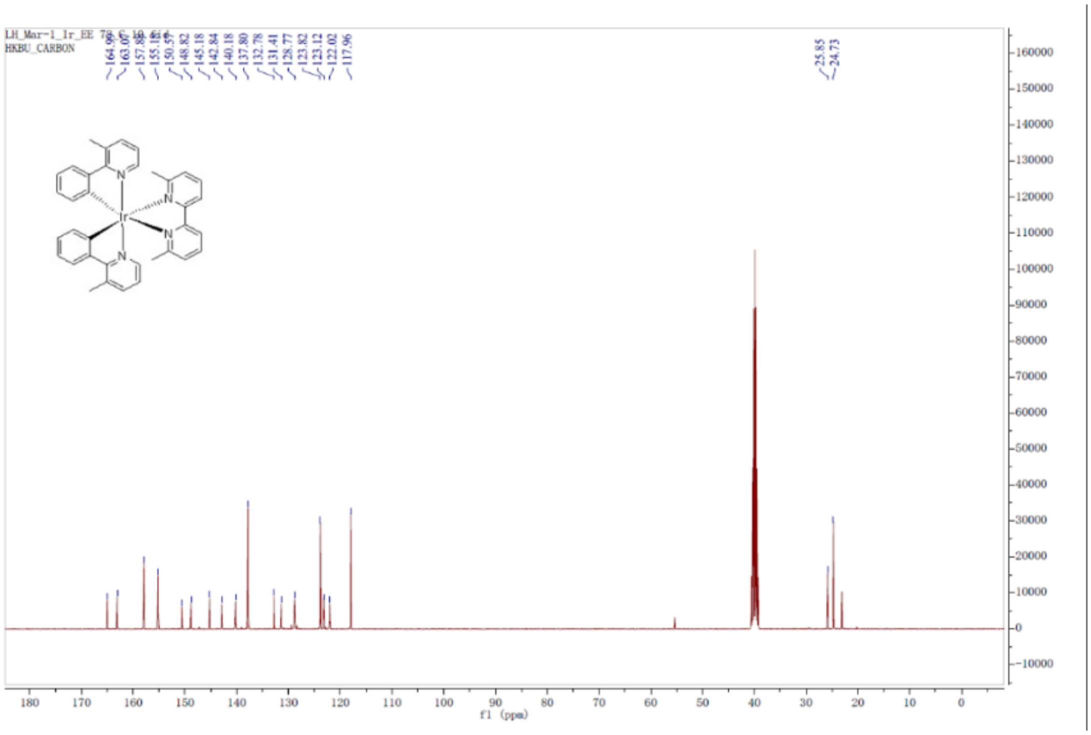
HKBU_CARBON
164.99
163.07
157.88
155.18
150.57
148.82
145.18
142.84
140.18
137.80
132.78
131.41
128.77
123.82
123.12
122.02
117.96
25.85
24.73
Ir
N
f1 (ppm)

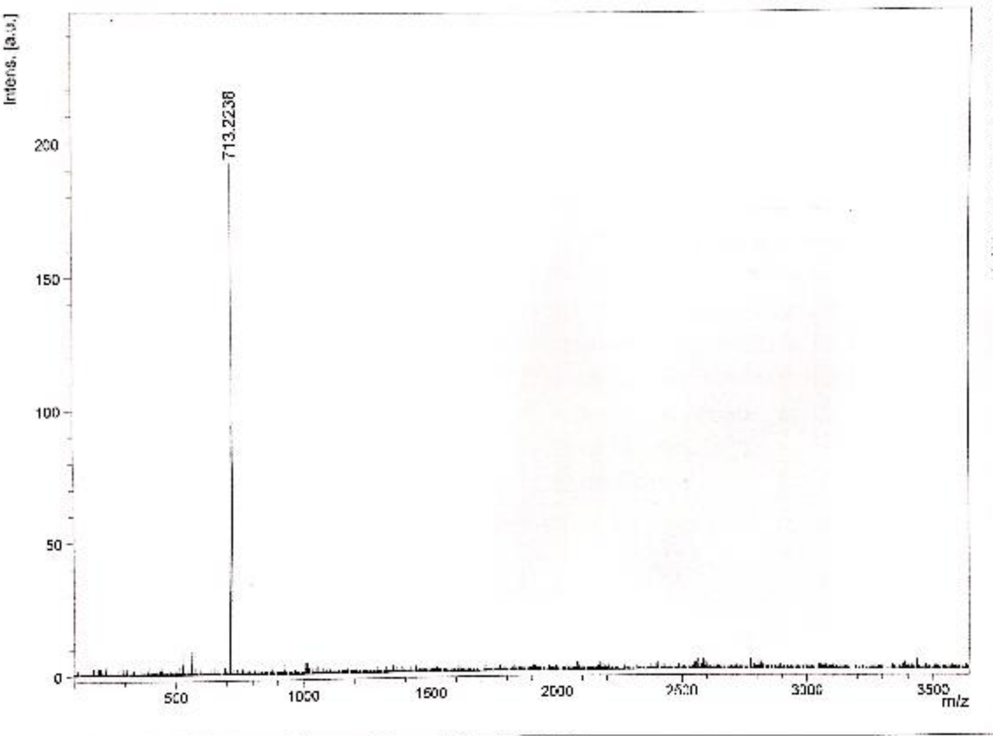
Intens. [a.u.]
713.2238
m/z

Complex **9**. Yield: 55%. ^{1}H NMR (400 MHz, Acetone-d_6) δ 9.42 (s, 1H), 9.34 (d, J = 8.8 Hz, 1H), 9.23 (d, J = 8.3, 1H), 8.78 (dd, J = 15.8, 5.1 Hz, 2H), 8.45 – 8.33 (m, 2H), 8.27 (ddd, J = 10.3, 8.5, 5.1 Hz, 2H), 8.07 – 7.94 (m, 2H), 7.82 (t, J = 5.6 Hz, 2H), 7.05 (dd, J = 7.4, 5.8 Hz, 2H), 6.82 (dd, J = 12.1, 9.3 Hz, 2H), 5.88 (d, J = 8.6 Hz, 2H). ^{13}C NMR (101 MHz, Acetone) δ 154.68, 153.19, 150.21, 148.28, 147.29, 141.23, 139.83, 135.74, 129.14, 128.55, 127.24, 124.61, 124.02, 123.73, 123.53, 114.01, 113.84. MALDI-TOF-HRMS: Calcd. for $C_{34}H_{19}F_4IrN_5O_2$ $[M–PF_6]^+$: 798.1104 Found: 798.0186. Anal.: ($C_{34}H_{19}F_4IrN_5O_2PF_6$) C, H, N: calcd. 43.32, 2.03, 7.43; found 43.46, 1.87, 7.42.

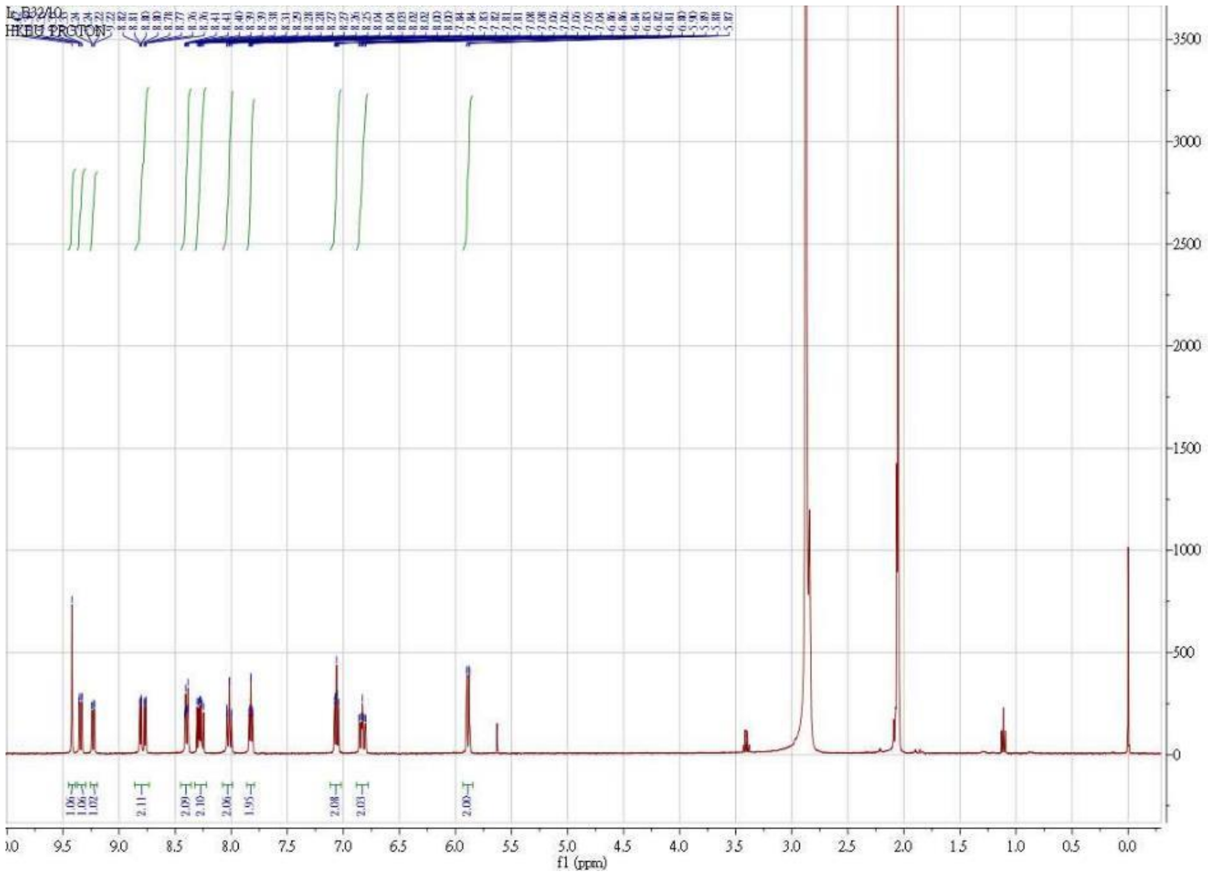

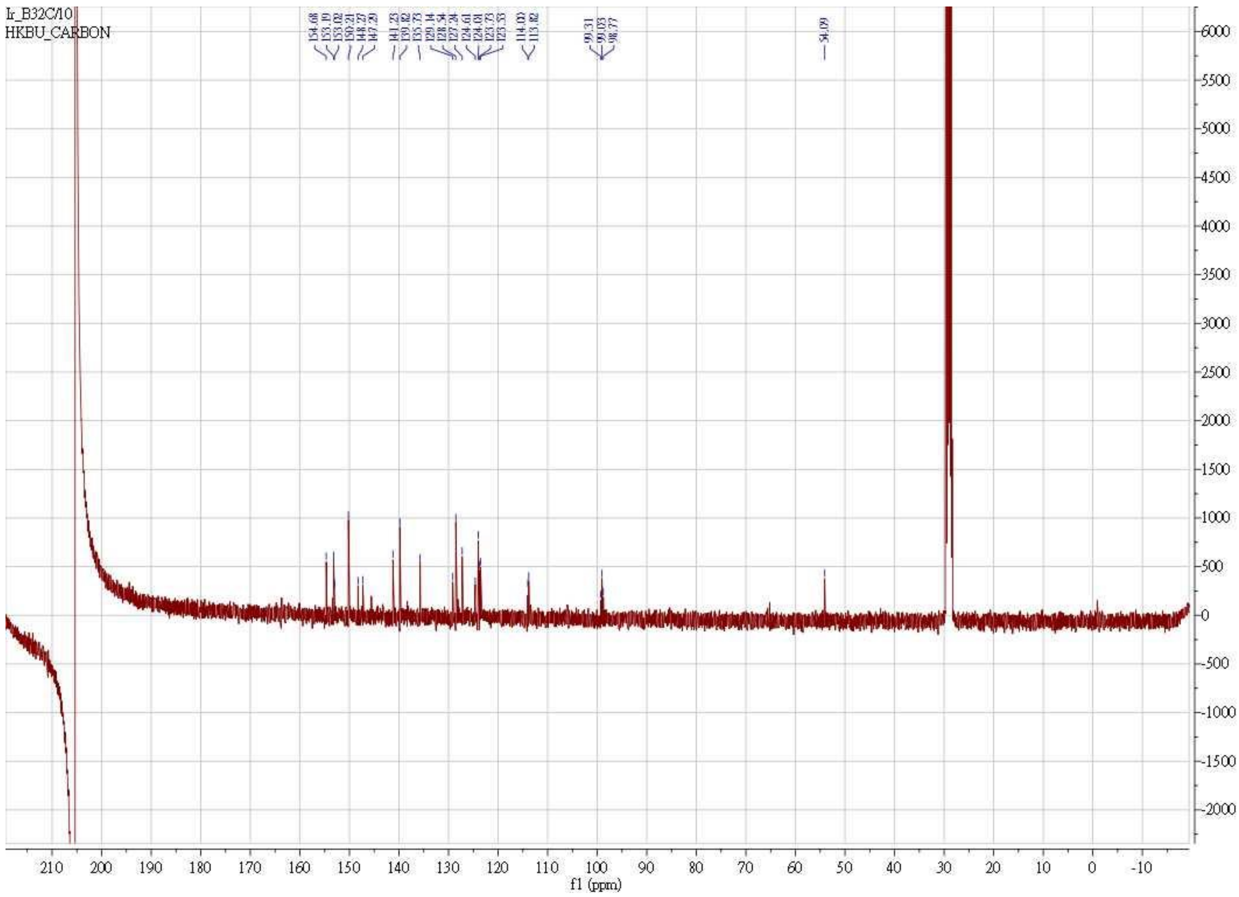
Ir_B32C/10
HKBU_CARBON
154.68
153.19
153.02
150.21
148.27
147.29
141.23
139.82
135.73
129.14
128.54
127.34
124.61
124.01
123.73
123.33
114.03
113.82
99.31
99.03
98.77
54.09
f1 (ppm)

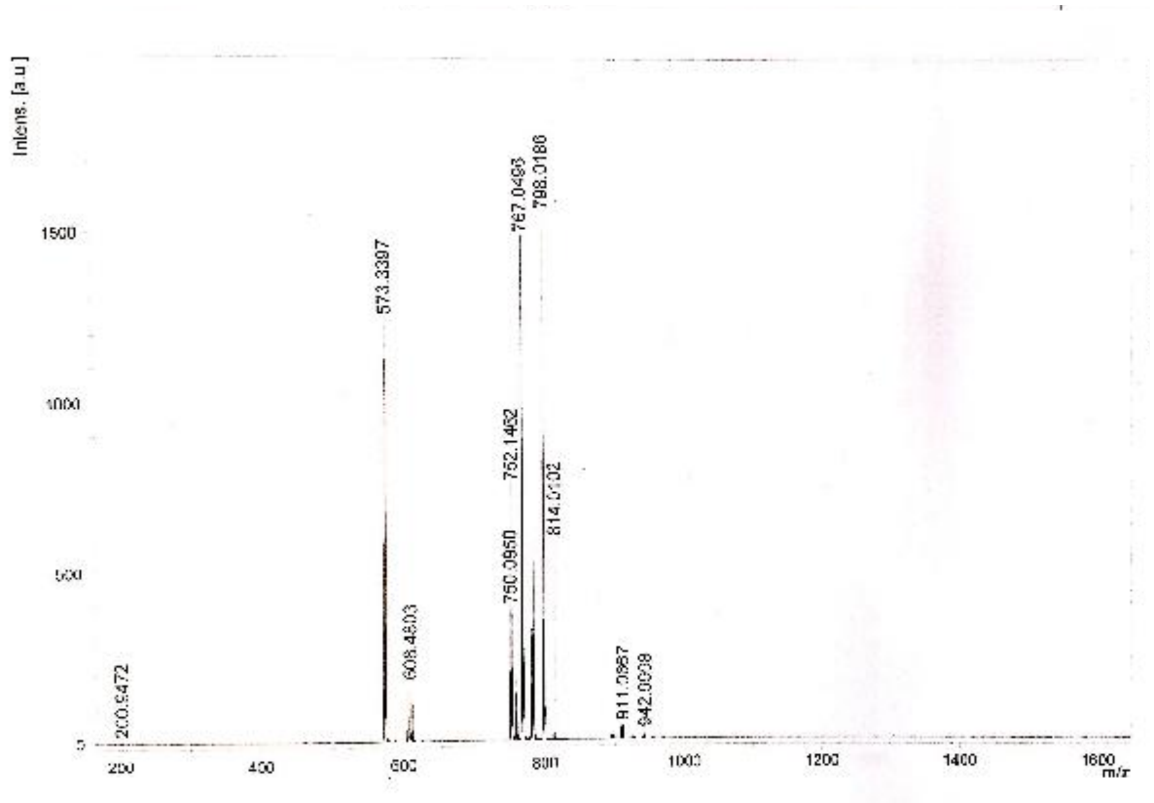
Intens. [a.u.]
573.3397
767.0493
798.0186
752.1452
814.0102
750.0950
608.4803
911.0987
942.0938
200.9472
m/z

Complex **10**. Yield: 78%. ^{1}H NMR (400 MHz, Acetone-d_6) δ 9.70 (dd, *J* = 8.3, 1.5 Hz, 2H), 8.72 (dd, *J* = 2.9, 0.7 Hz, 2H), 8.64 (dd, *J* = 5.1, 1.5 Hz, 2H), 8.18 (dd, *J* = 8.3, 5.1 Hz, 2H), 7.68 (dd, *J* = 8.0, 1.2 Hz, 2H), 7.23 (dd, *J* = 2.4, 0.6 Hz, 2H), 7.18 – 7.07 (m, 2H), 6.93 (td, *J* = 7.4, 1.2 Hz, 2H), 6.61 (dd, *J* = 2.9, 2.3 Hz, 2H), 6.46 (dd, *J* = 7.4, 1.3 Hz, 2H), 3.35 – 3.19 (m, 4H), 2.16 – 2.12 (h, *J* = 3.4 Hz, 4H). ^{13}C NMR (101 MHz, Acetone) δ 157.20, 153.04, 149.68, 144.31, 139.86, 137.79, 135.66, 134.06, 132.47, 131.18, 128.87, 128.27, 127.42, 124.21, 112.83, 109.05, 33.63, 23.12. MALDI-TOF-HRMS: Calcd. for $C_{38}H_{30}IrN_6$ $[M–PF_6]^+$: 763.2161 Found: 765.3537. Anal.: ($C_{38}H_{30}IrN_6PF_6$) C, H, N: calcd. 47.52, 3.10, 12.32; found 47.66, 3.19, 12.45.

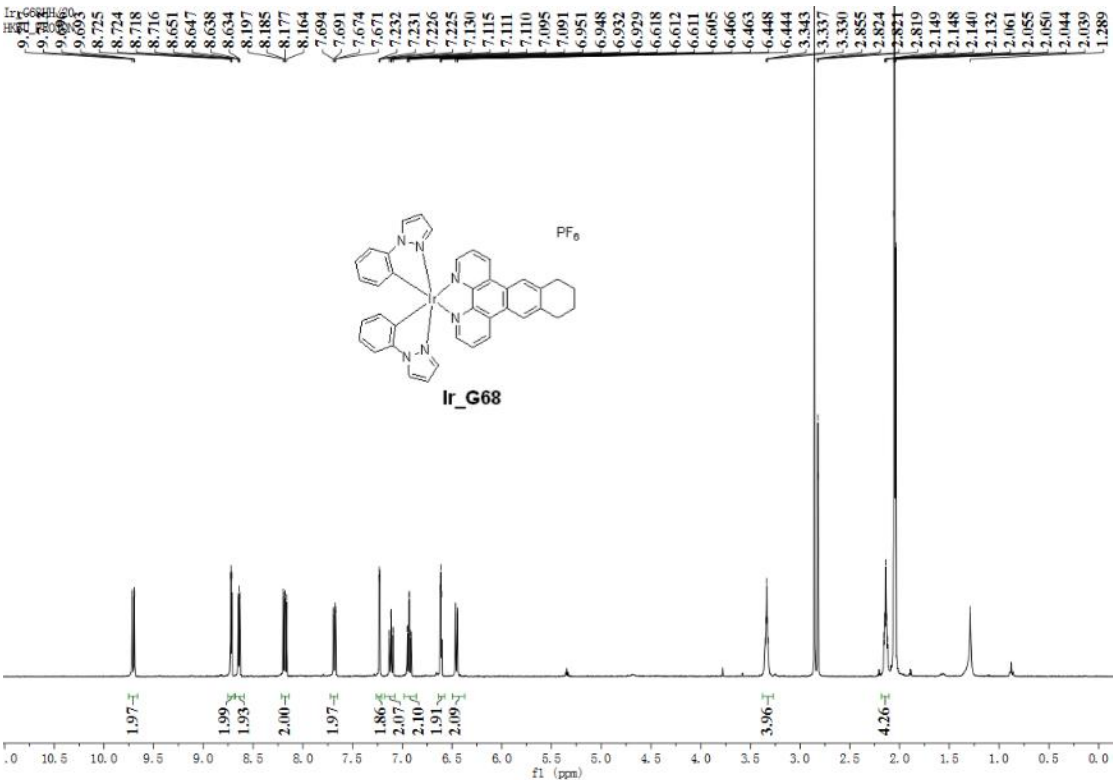

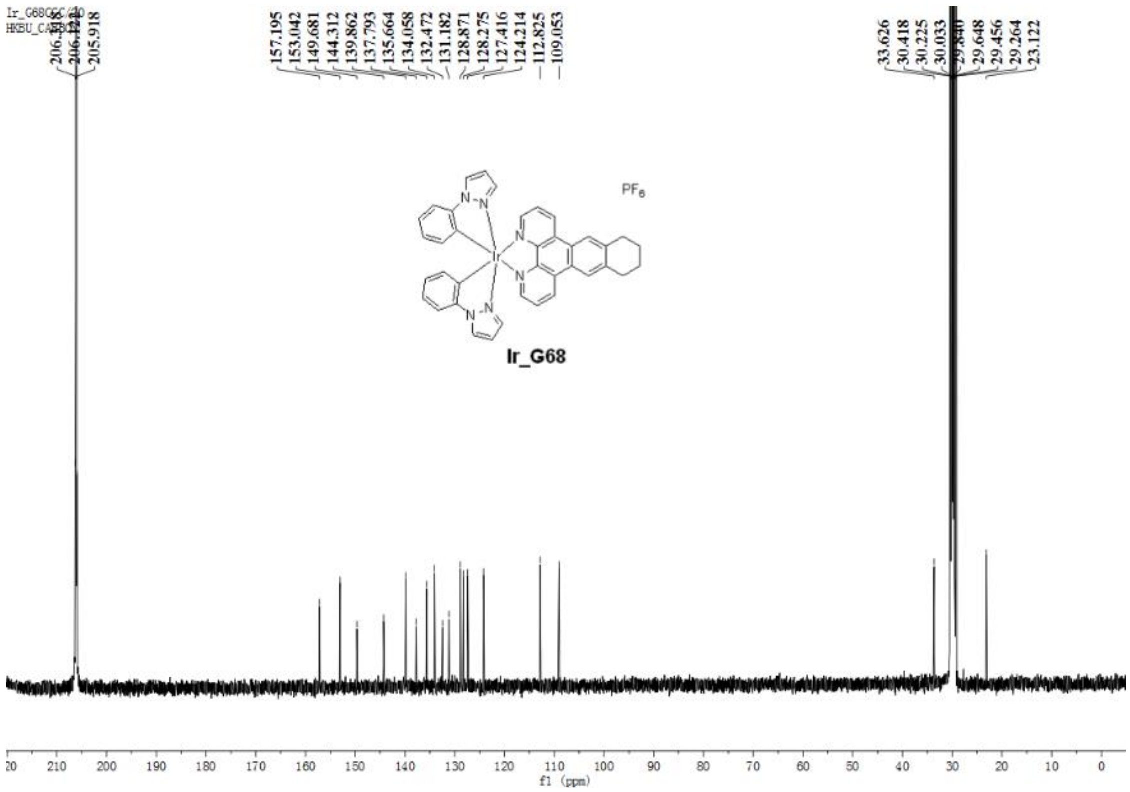
Ir_G68
PF6
f1 (ppm)

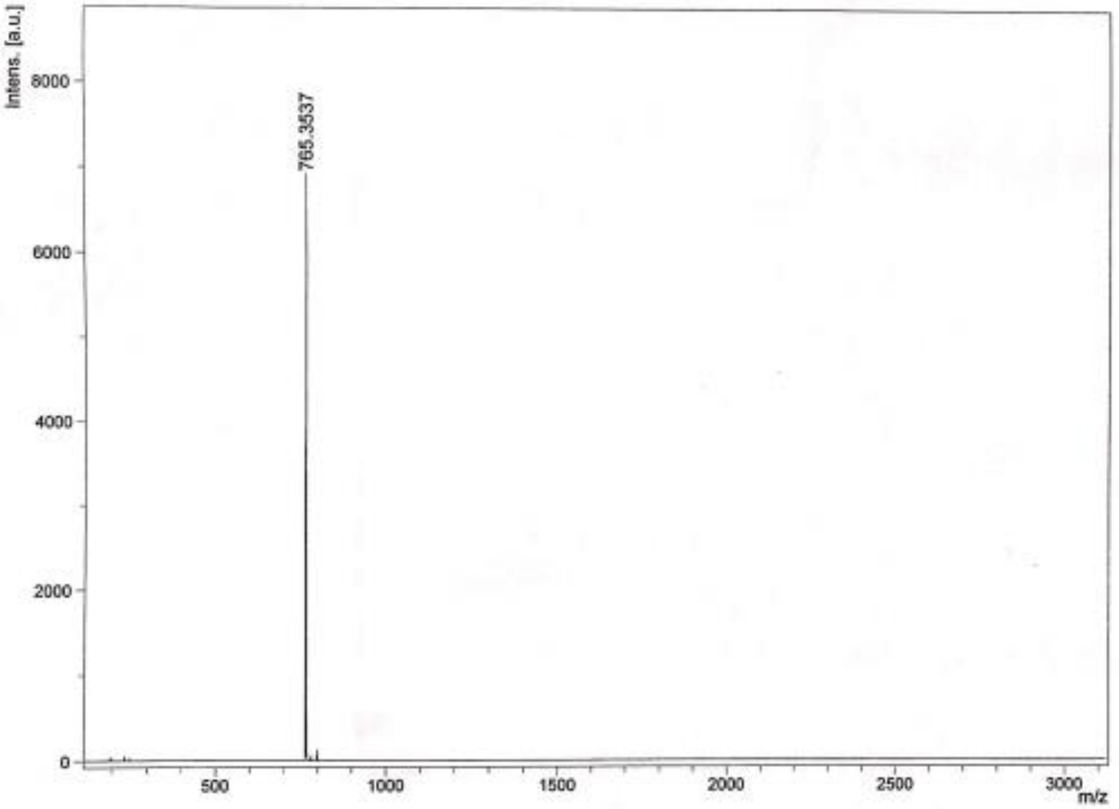
Intens. [a.u.]
765.3537
m/z

Complex **11**. Yield: 72%. ^{1}H NMR (400 MHz, DMSO-d_6) δ 9.63 (d, J = 8.3 Hz, 2H), 9.27 (s, 1H), 8.33 – 8.25 (m, 4H), 8.17 (dd, J = 8.3, 5.2 Hz, 2H), 7.96 (d, J = 8.0 Hz, 2H), 7.89 (t, J = 8.2 Hz, 2H), 7.61 – 7.54 (m, 2H), 7.07 (t, J = 7.6 Hz, 2H), 7.03 – 6.93 (m, 4H), 6.29 (d, J = 7.7 Hz, 2H), 2.92 (s, 3H). ^{13}C NMR (101 MHz, DMSO) δ 166.76, 156.31, 151.61, 151.32, 149.70, 149.49, 149.45, 148.08, 147.59, 147.20, 144.02, 138.75, 138.24, 136.81, 134.80, 134.56, 131.17, 130.27, 130.02, 129.86, 128.20, 128.10, 125.08, 123.81, 122.45, 119.96, 22.23. MALDI-TOF-HRMS: Calcd. for $C_{37}H_{26}IrN_6$ [M–PF_6]$^+$: 747.1848 Found: 747.1835. Anal.: ($C_{37}H_{26}IrN_6PF_6$ + $2H_2O$) C, H, N: calcd. , 47.90, 3.26, 9.06; found 47.76, 3.13, 9.22.

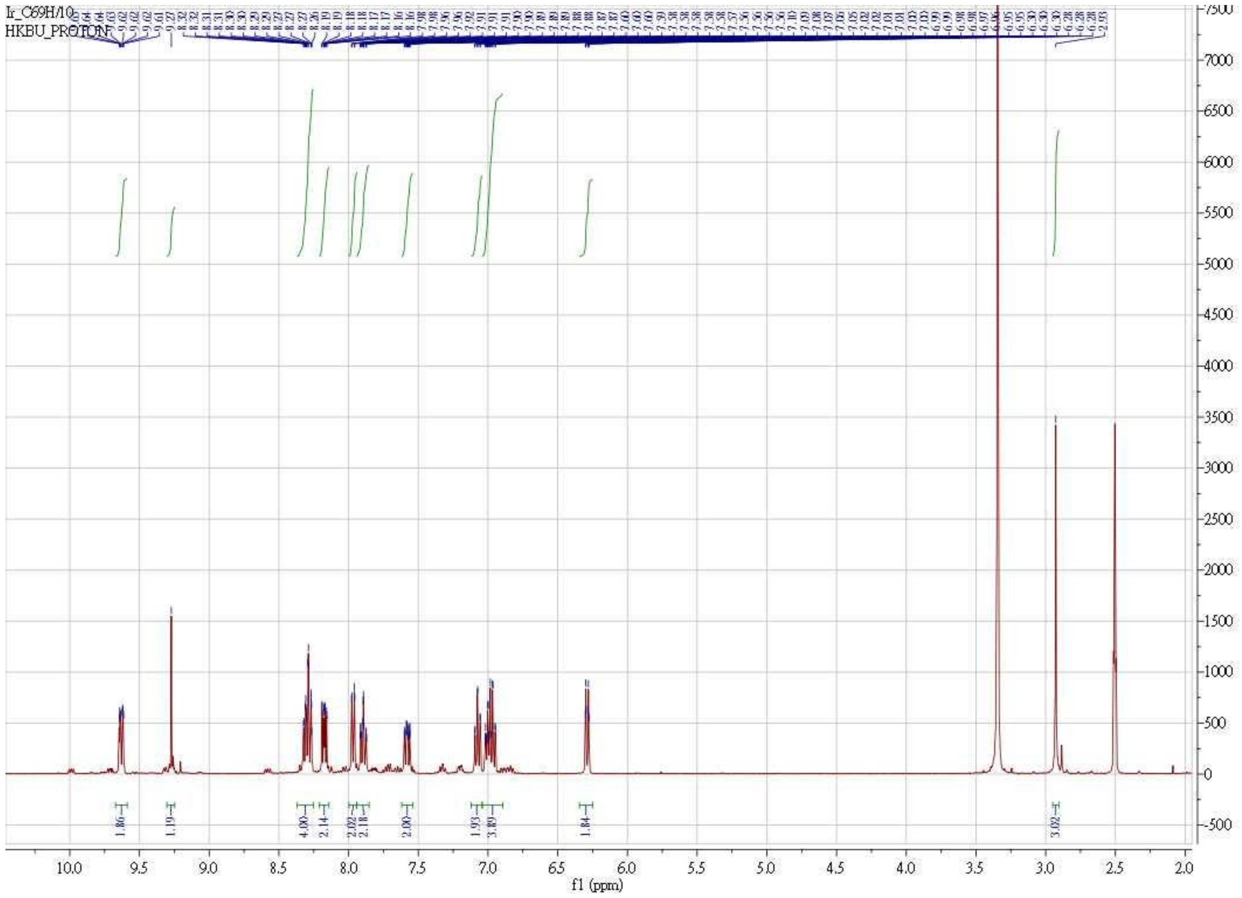

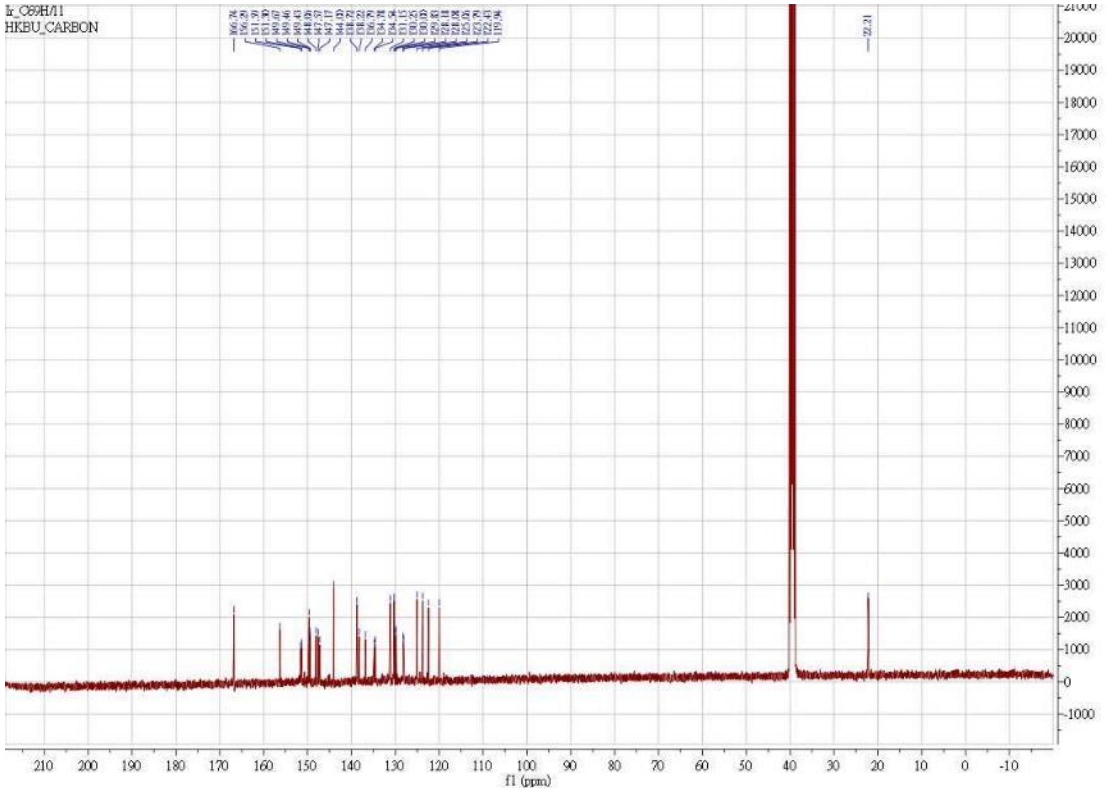
HKBU_CARBON
22.21
f1 (ppm)

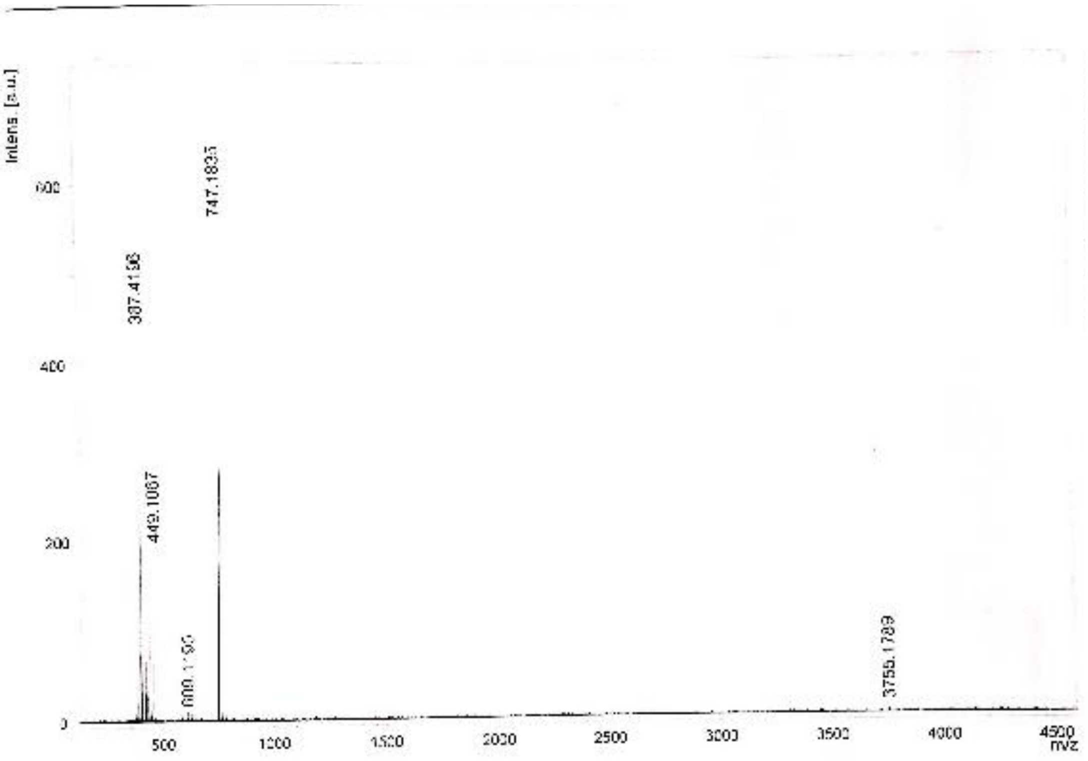
Intens. [a.u.]
747.1835
449.1067
3755.1789
m/z

Complex **12**. Yield: 60%. ^{1}H NMR (400 MHz, Acetone-d_6) δ 8.68 (d, J = 1.7 Hz, 2H), 8.13 (dd, J = 8.2, 1.4 Hz, 2H), 7.97 (d, J = 5.7 Hz, 2H), 7.94 (dd, J = 8.0, 1.3 Hz, 2H), 7.83 (t, J = 7.8 Hz, 2H), 7.49 (dd, J = 5.7, 1.7 Hz, 2H), 7.14 – 7.02 (m, 4H), 6.87 (t, J = 7.4, 1.3 Hz, 2H), 6.45 (d, J = 7.6 Hz, 2H), 2.86 – 2.79 (m, 4H), 1.89 (s, 6H), 1.75 – 1.63 (m, 4H), 1.33 – 1.23 (m, 24H), 0.89 – 0.84 (m, 6H). ^{13}C NMR (101 MHz, Acetone) δ 169.60, 162.51, 156.94, 156.84, 149.59, 149.13, 146.52, 139.94, 133.85, 130.47, 128.86, 126.26, 125.31, 124.74, 123.56, 118.47, 35.78, 32.57, 30.85, 30.17, 30.09, 29.99, 29.95, 29.90, 29.81, 26.36, 23.31, 14.35. MALDI-TOF-HRMS: Calcd. for $C_{52}H_{64}IrN_4$ $[M–PF_6]^+$: 937.4760 Found: 937.5958. Anal.: ($C_{52}H_{64}F_6IrN_4P$) C, H, N: calcd. 57.71, 5.96, 5.18; found 57.87, 6.05, 5.16.

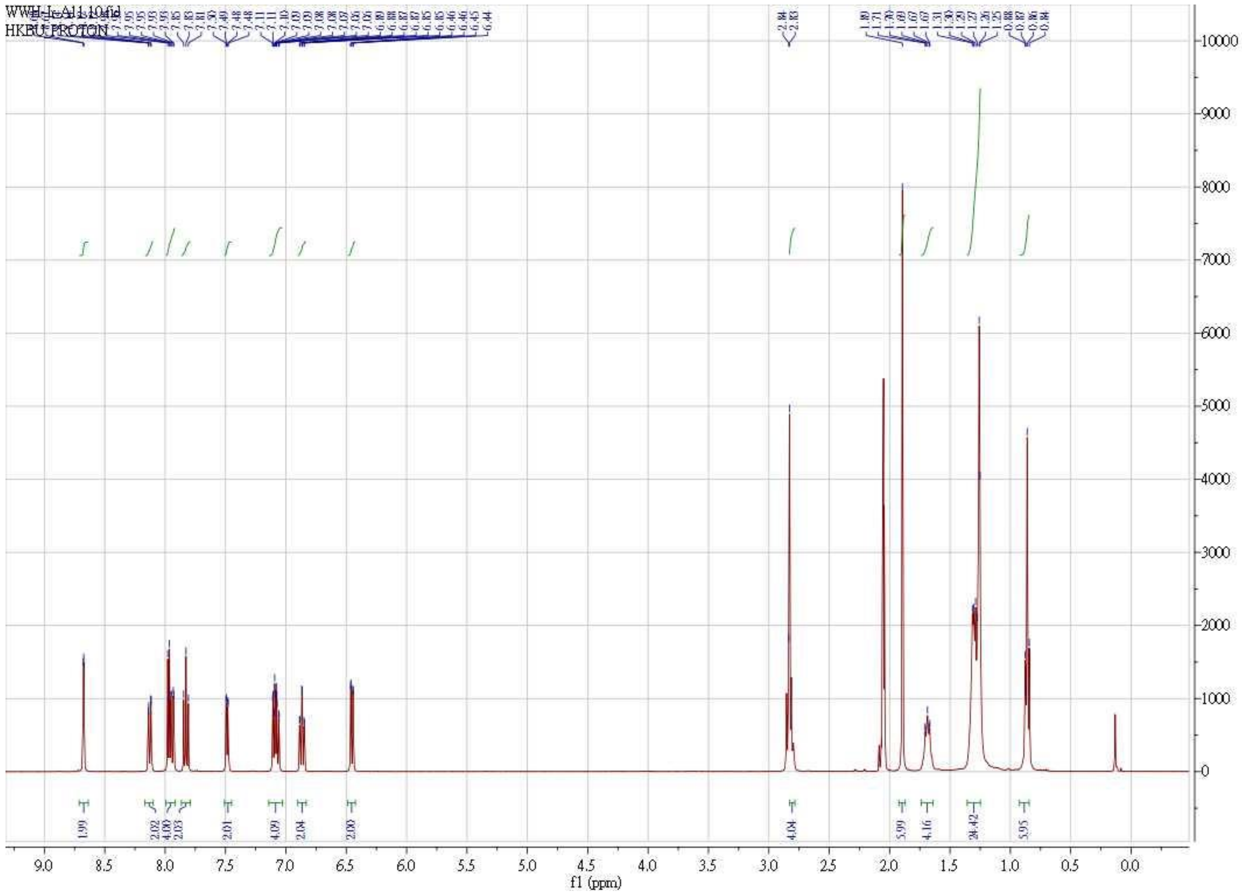

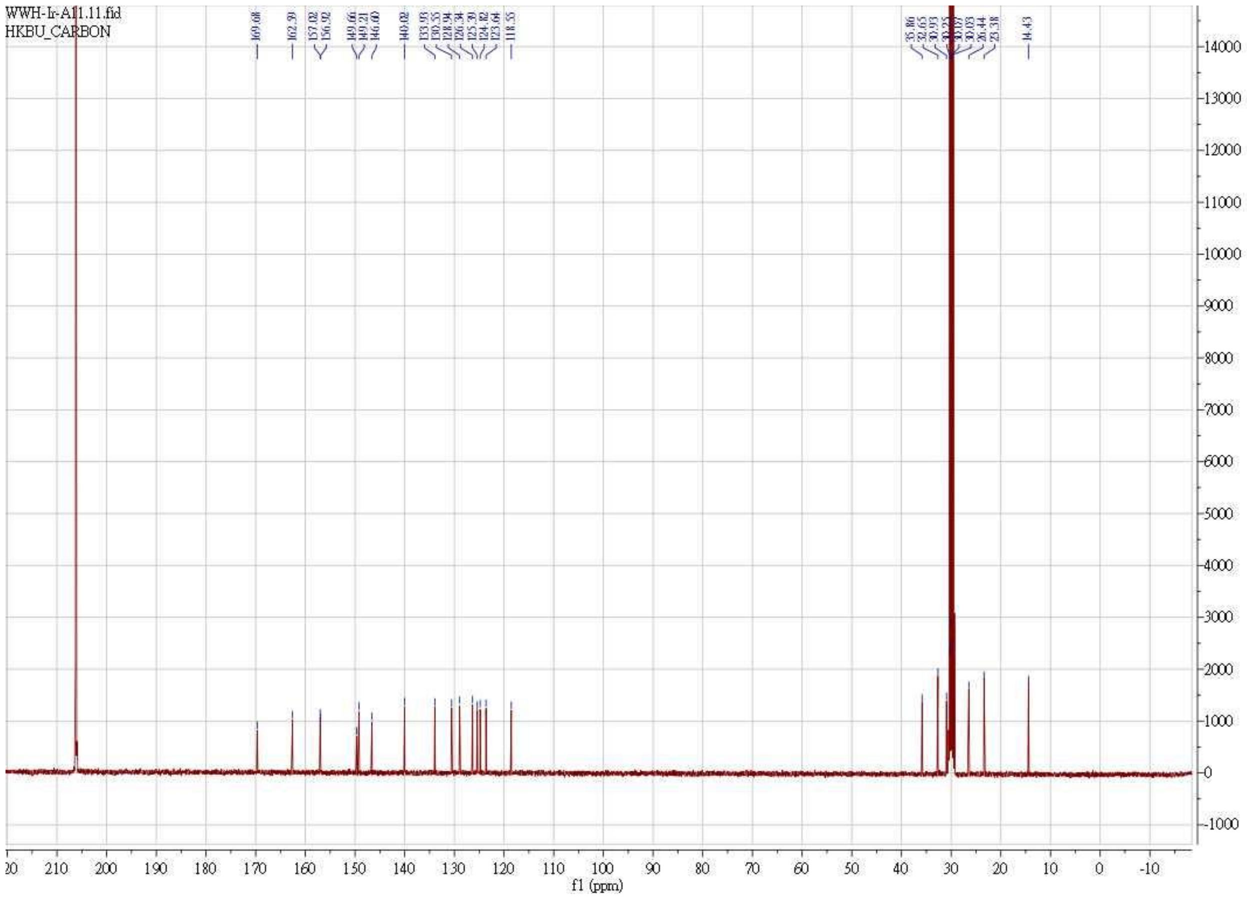
WWH-Ir-A11.11.fid
HKBU_CARBON
169.68
162.93
157.02
156.92
149.66
149.21
146.00
140.02
133.93
130.55
128.94
126.34
125.39
124.82
123.64
118.55
35.86
32.65
30.93
30.03
26.44
23.38
14.43
f1 (ppm)

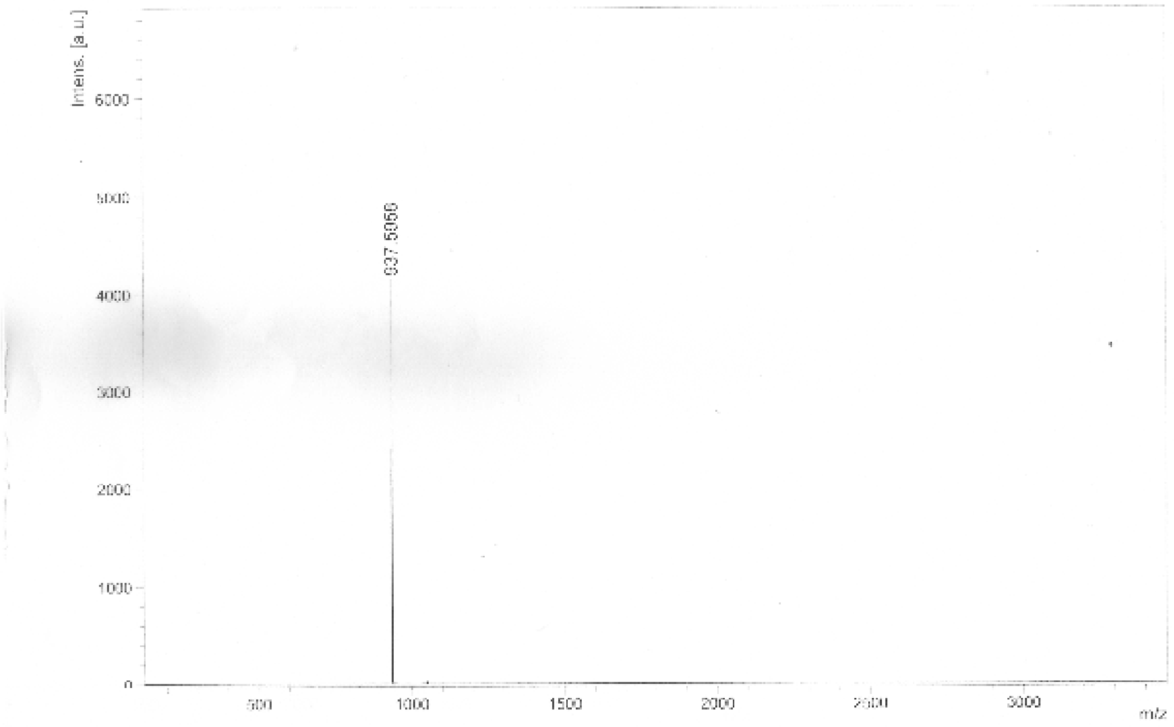
Intens. [a.u.]
937.5050
m/z

Complex **13**. Yield: 46%. ^{1}H NMR (400 MHz, DMSO-d_6) δ 9.63 (d, J = 8.2 Hz, 2H), 9.10 (d, J = 8.1 Hz, 2H), 8.41 – 8.31 (m, 4H), 8.29 (d, J = 8.4 Hz, 2H), 8.19 (dd, J = 8.3, 5.0 Hz, 2H), 7.95 – 7.91 (m, 4H), 7.71 (d, J = 5.9 Hz, 2H), 7.60 (d, J = 7.6 Hz, 2H), 7.55 (t, J = 8.1 Hz, 2H), 7.05 (t, J = 6.8 Hz, 2H), 6.96 (d, J = 8.3 Hz, 2H), 6.21 – 6.14 (m, 2H), 2.16 (s, 6H). ^{13}C NMR (101 MHz, DMSO) δ 166.93, 151.60, 150.06, 149.30, 148.23, 141.43, 140.87, 139.73, 138.76, 137.62, 135.01, 131.95, 131.32, 130.90, 129.98, 128.23, 128.11, 125.62, 125.16, 123.58, 123.34, 123.22, 119.72, 21.62. MALDI-TOF-HRMS: Calcd. for $C_{50}H_{34}IrN_6$ $[M–PF_6]^+$: 911.2474 Found: 911.2397. Anal.: ($C_{50}H_{34}IrN_6PF_6$ + 2.5H_2O) C, H, N: calcd. 54.54, 3.57, 7.63; found 54.35, 3.07, 7.35.

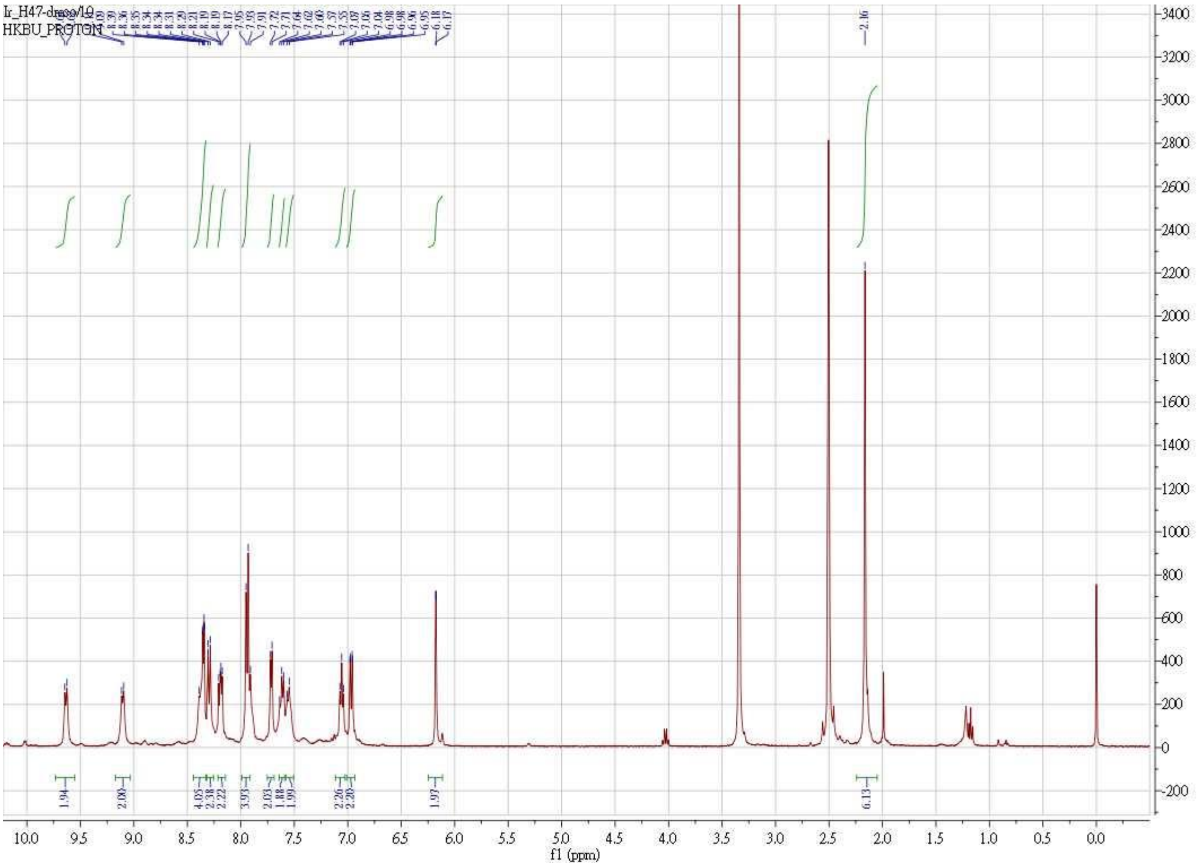

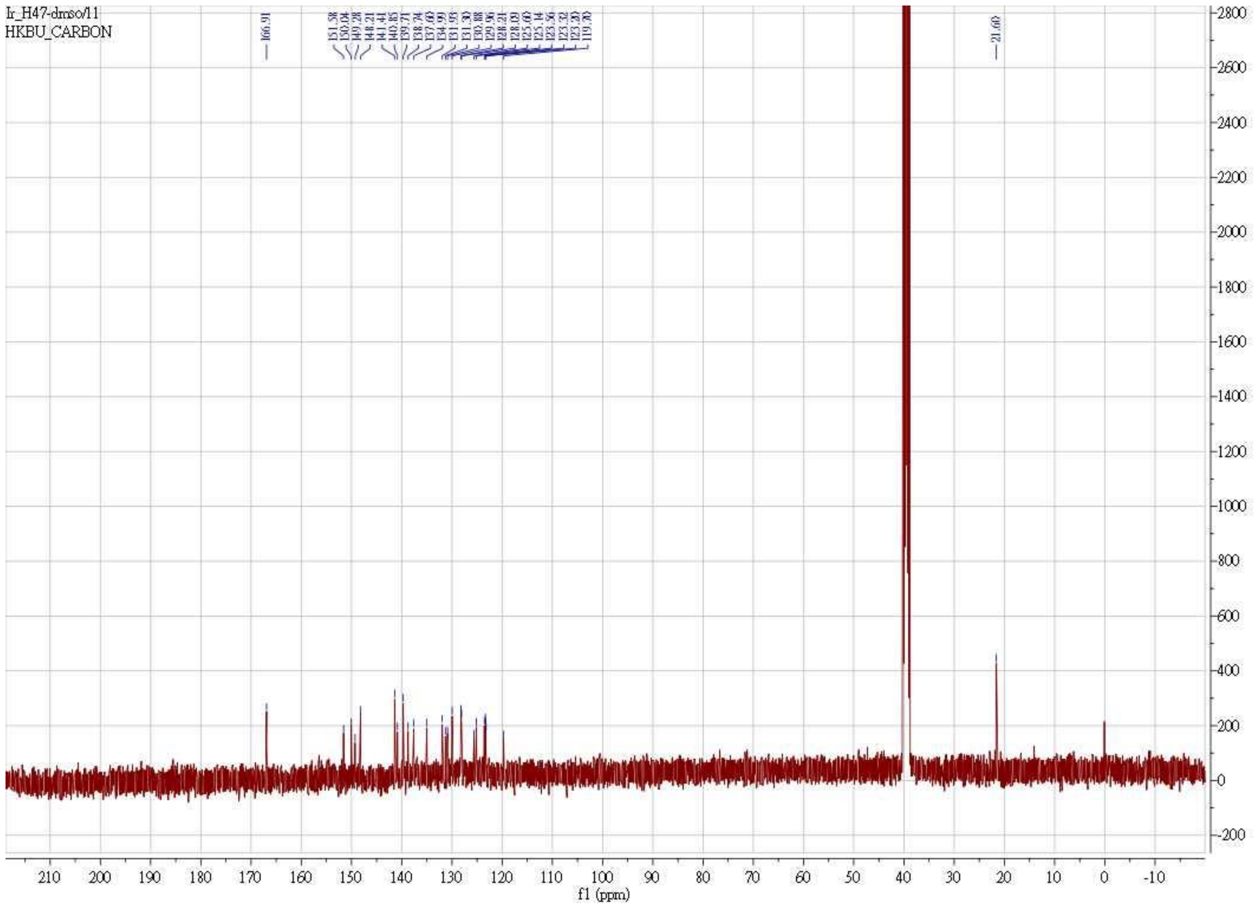
Ir_H47-dmso/11
HKBU_CARBON
166.91
151.58
150.04
149.28
148.21
141.41
140.85
139.71
138.74
137.60
134.99
131.93
131.30
130.88
129.96
128.21
128.09
125.60
125.14
123.56
123.32
123.20
119.70
21.60
f1 (ppm)

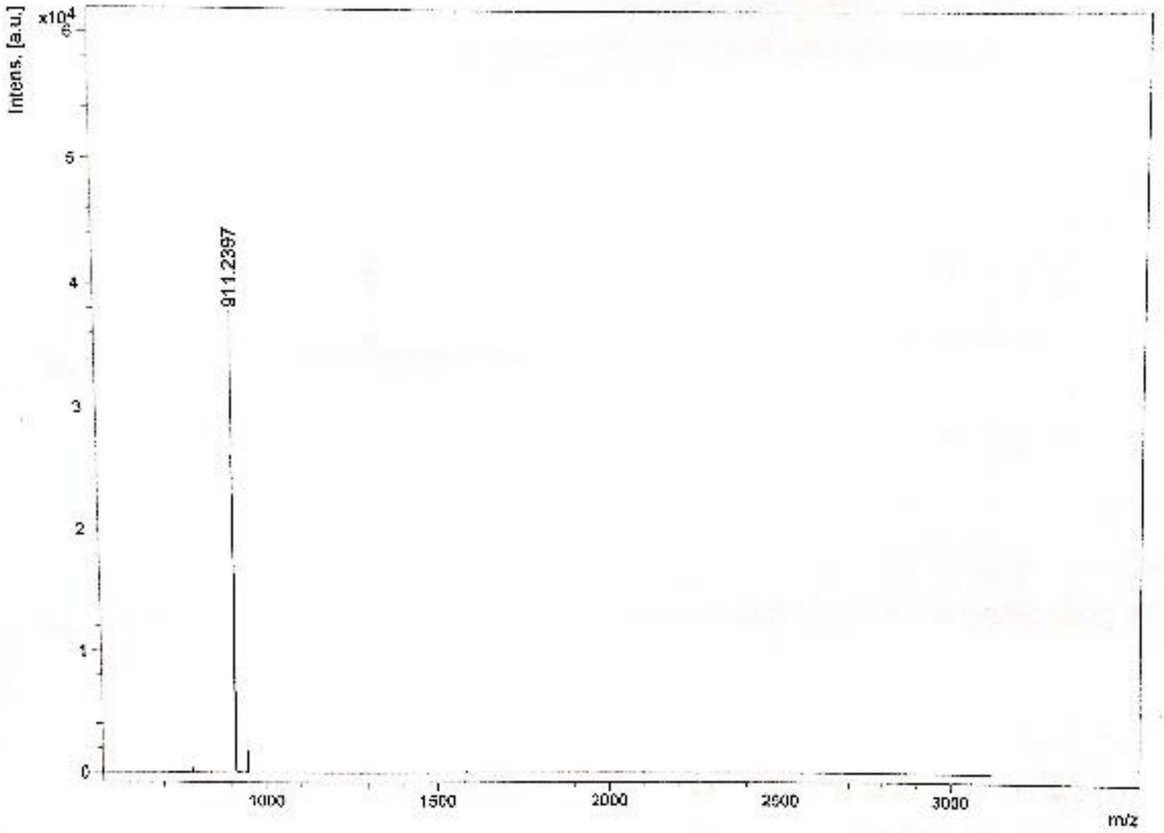
Intens. [a.u.]
x10^4
911.2397
m/z

Complex **14**. Yield: 61%. ^{1}H-NMR (400 MHz; DMSO-d_6): 8.60 (m, 3H), 8.29 (d, J = 4.0 Hz, 1H), 8.23 (d, J = 8.0 Hz, 3H), 8.11 (d, J = 8.0 Hz, 2H), 7.39 (t, J = 6.0 Hz, 2H), 7.23 (t, J = 8.0 Hz, 2H), 7.09 (t, J = 9.0 Hz, 2H), 7.03 (t, J = 6.0 Hz, 2H), 6.40 (t, J = 8.0 Hz, 2H), 5.96 (d, J = 12.0 Hz, 1H), 5.89 (d, J = 8.0 Hz, 1H), 5.81 (s, 1H). ^{13}C-NMR (400 MHz; DMSO-d_6): δ 181.7, 152.0, 149.6, 149.0, 147.6, 140.6, 137.2, 133.5, 133.2, 132.4, 131.6, 131.1, 128.7, 127.5, 126.4, 125.0, 124.3, 123.6, 123.2, 117.0, 58.2, 55.3. MALDI-TOF-HRMS: Calcd. for $C_{38}H_{22}Br_2IrN_4S_2$ $[M–PF_6]^+$: 950.9261 Found: 950.9788. Anal.: ($C_{38}H_{22}IrBr_2N_4S_2PF_6$) C, H, N: calcd. 41.65, 2.02, 5.11; found 41.08, 1.98, 5.31.

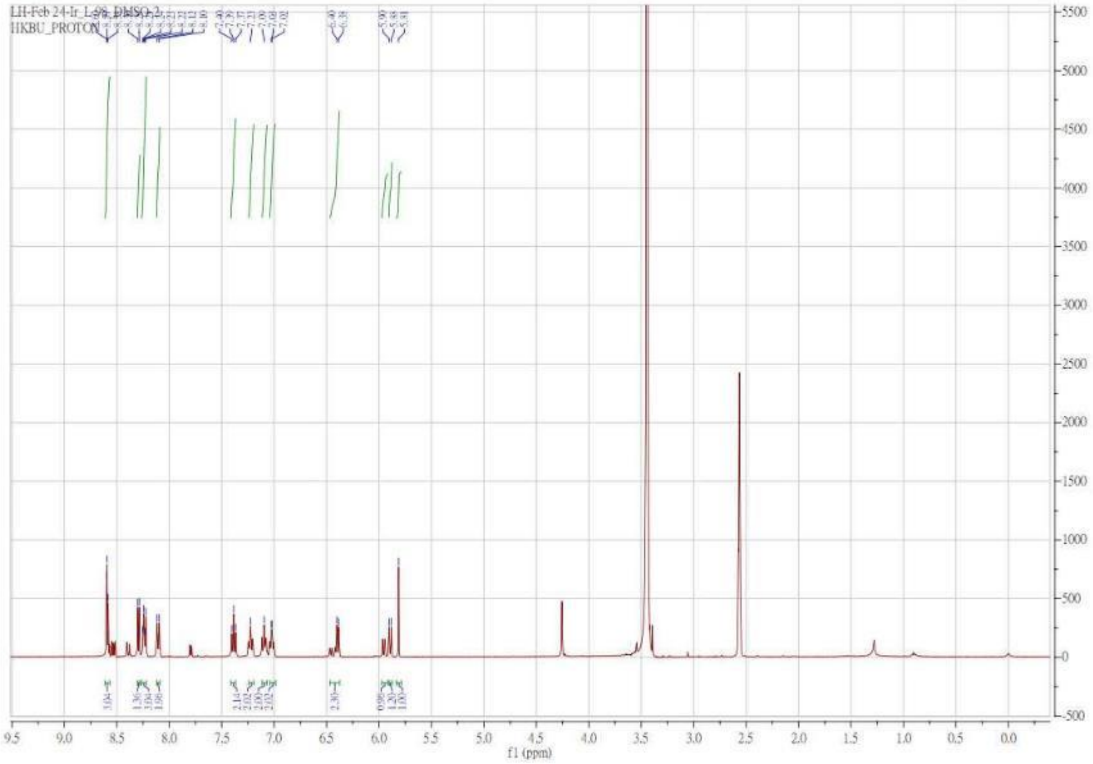

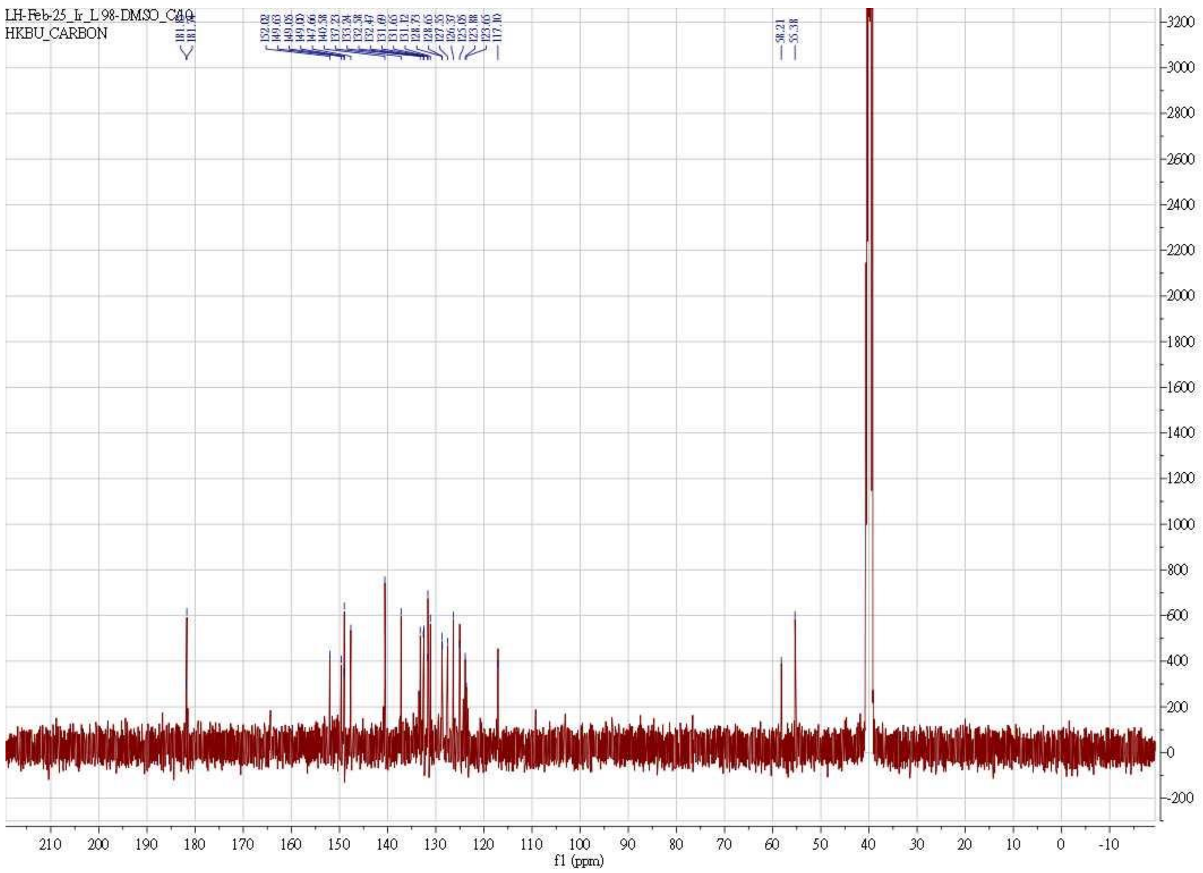
LH-Feb-25_Ir_L98-DMSO_C
HKBU_CARBON
f1 (ppm)

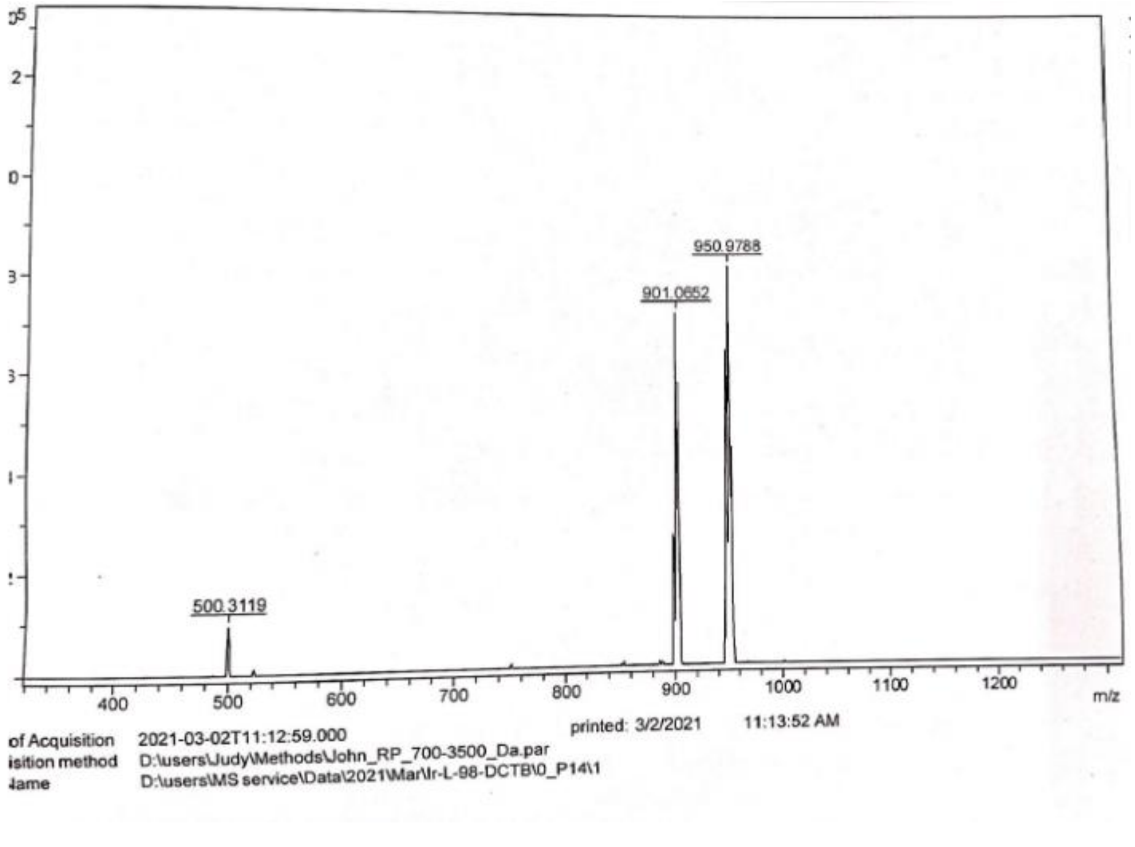
950.9788
901.0652
500.3119
m/z
printed: 3/2/2021 11:13:52 AM
of Acquisition 2021-03-02T11:12:59.000
isition method D:\users\Judy\Methods\John_RP_700-3500_Da.par
lame D:\users\MS service\Data\2021\Mar\Ir-L-98-DCTB\0_P14\1

Complex **1a**. Yield: 59%. ^{1}H NMR (400 MHz, Acetone-d_6) δ 8.42 (s, 2H), 8.22 (dt, J = 8.3, 1.2 Hz, 2H), 8.17 (d, J = 6.1 Hz, 2H), 7.94 – 7.86 (m, 4H), 7.75 (ddd, J = 5.8, 1.5, 0.8 Hz, 2H), 7.52 (d, J = 6.1 Hz, 2H), 7.08 – 6.96 (m, 4H), 6.93 (td, J = 7.4, 1.4 Hz, 2H), 6.45 (dd, J = 7.5, 1.2 Hz, 2H), 4.25 (s, 6H). ^{13}C NMR (101 MHz, Acetone-d$_6$) δ 167.94, 163.95, 152.39, 150.58, 149.18, 147.29, 144.38, 138.32, 131.88, 130.18, 124.81, 123.59, 123.29, 122.23, 121.06, 119.66, 106.87, 57.02, 29.56, 29.37, 29.23, 29.17, 29.04, 28.98, 28.79, 28.60, 28.40. HRMS $[C_{36}H_{28}IrN_4O_2]^+$ calculated: 741.1842, found: 741.1884. Anal. ($C_{36}H_{28}O_2IrN_4PF_6$) C, H, N: calculated: 48.81, 3.19, 6.32; found: 48.53, 3.20, 6.30.

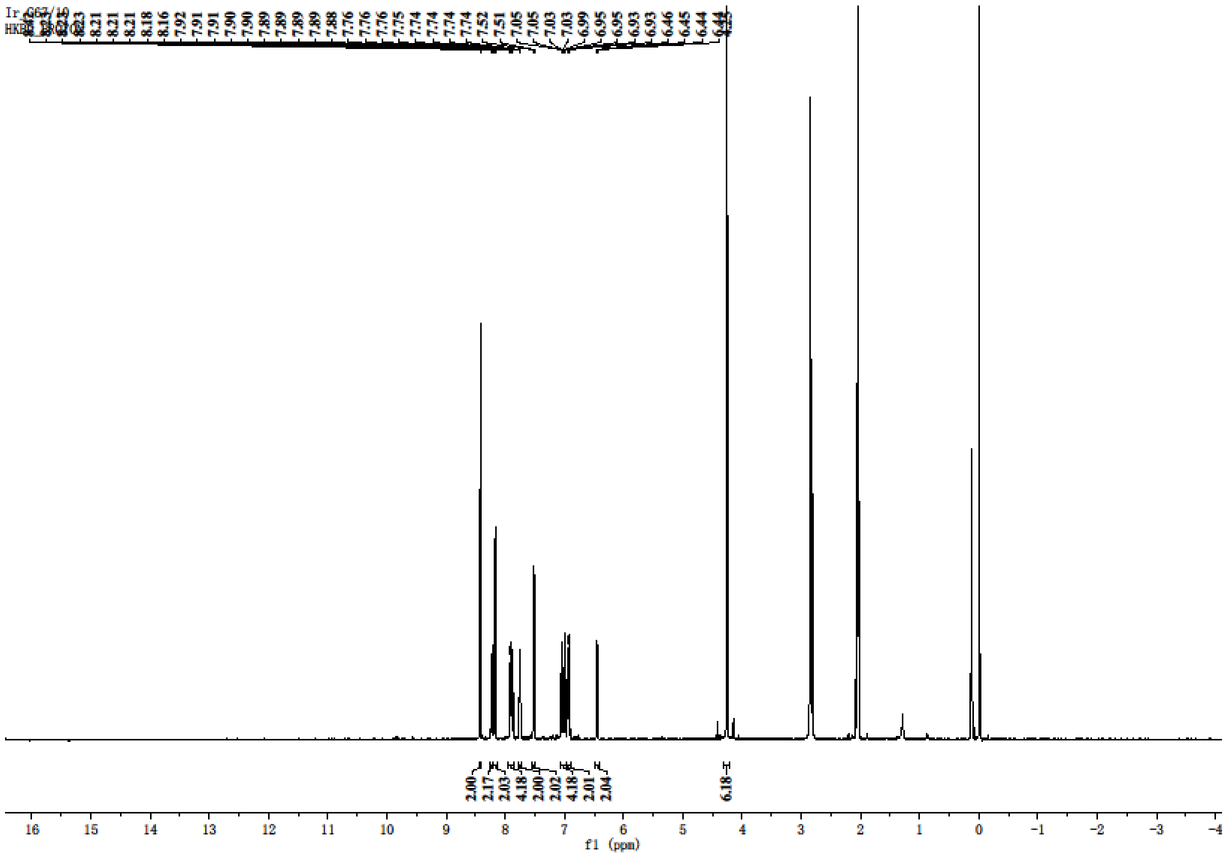

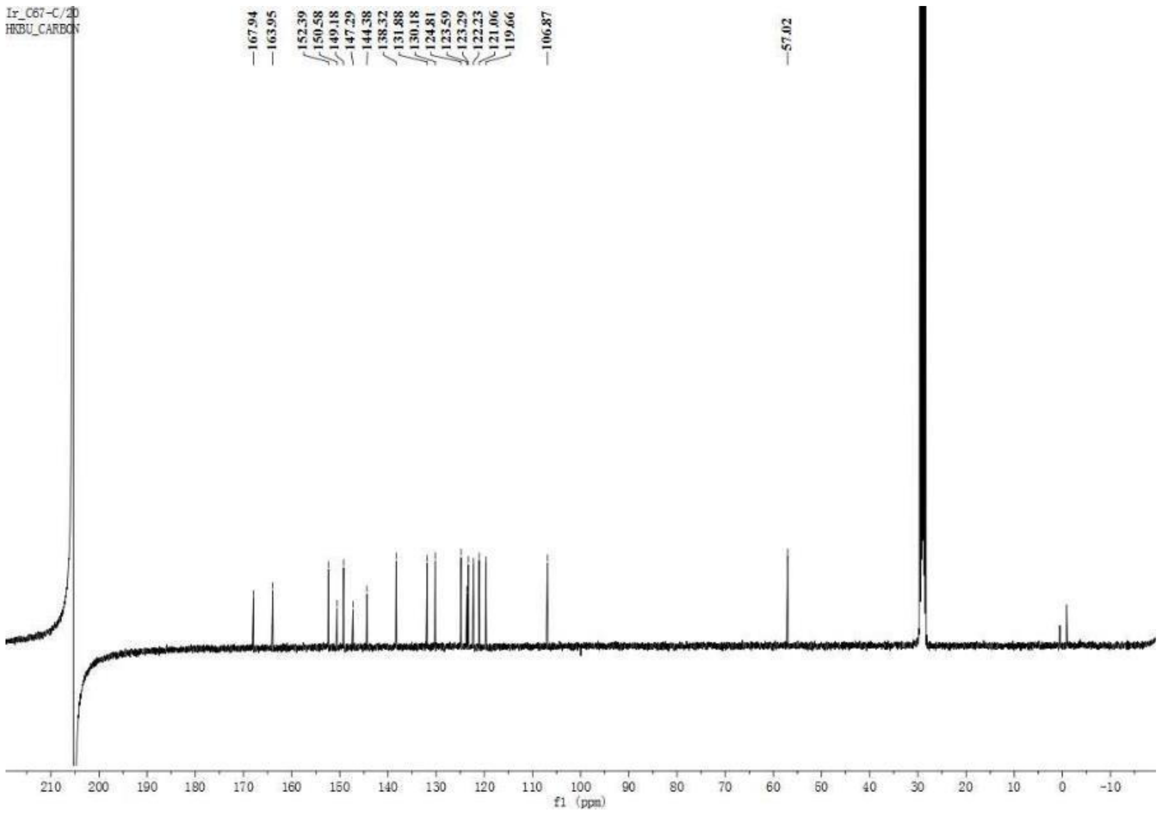
Ir_C67-C/20
HKBU_CARBON
167.94
163.95
152.39
150.58
149.18
147.29
144.38
138.32
131.88
130.18
124.81
123.59
123.29
122.23
121.06
119.66
106.87
57.02
f1 (ppm)

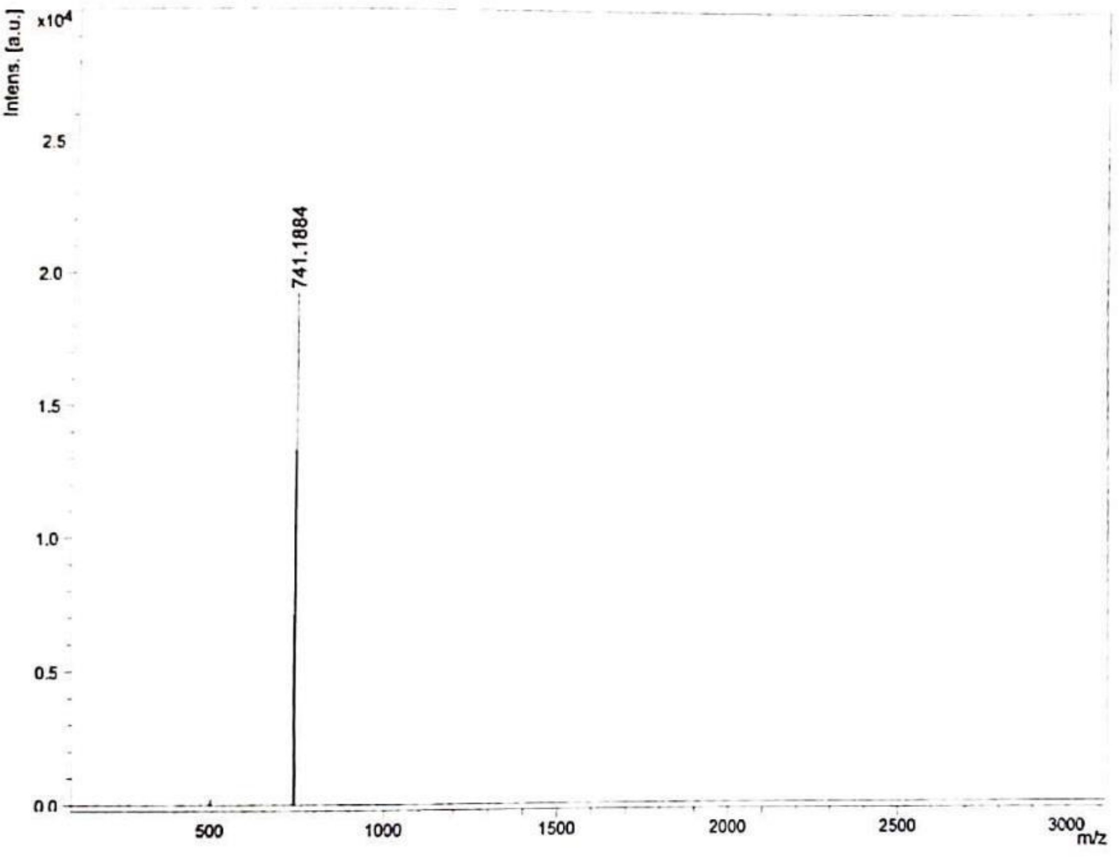
Intens. [a.u.]
x10^4
741.1884
m/z

Complex **1b**. Yield: 68%.^{1}H NMR (400 MHz, Acetone-d_6) δ 8.41 (s, 2H), 8.26 – 8.20 (m, 4H), 8.00 – 7.94 (m, 4H), 7.73 – 7.68 (m, 2H), 7.49 (d, J = 6.0 Hz, 2H), 7.12 (td, J = 7.5, 1.2 Hz, 2H), 7.08 – 6.98 (m, 4H), 6.45 (dt, J = 7.3, 1.1 Hz, 2H), 4.24 (s, 6H), 13C NMR (101 MHz, Acetone) δ 166.02, 164.92, 152.76, 150.15, 146.92, 145.07, 139.39, 133.74, 130.81, 125.48, 124.27, 124.13, 123.89, 121.56, 120.77, 120.76, 107.49, 57.81; MALDI-TOF-HRMS: Calcd. for $C_{36}H_{28}N_4Rh[M–PF_6]^+$: 651.1261 Found: 651.1235; Anal. ($C_{36}H_{28}N_4RhPF_6$) C, H, N: calcd 54.29, 3.54, 7.03; found 54.01, 3.73, 7.01.

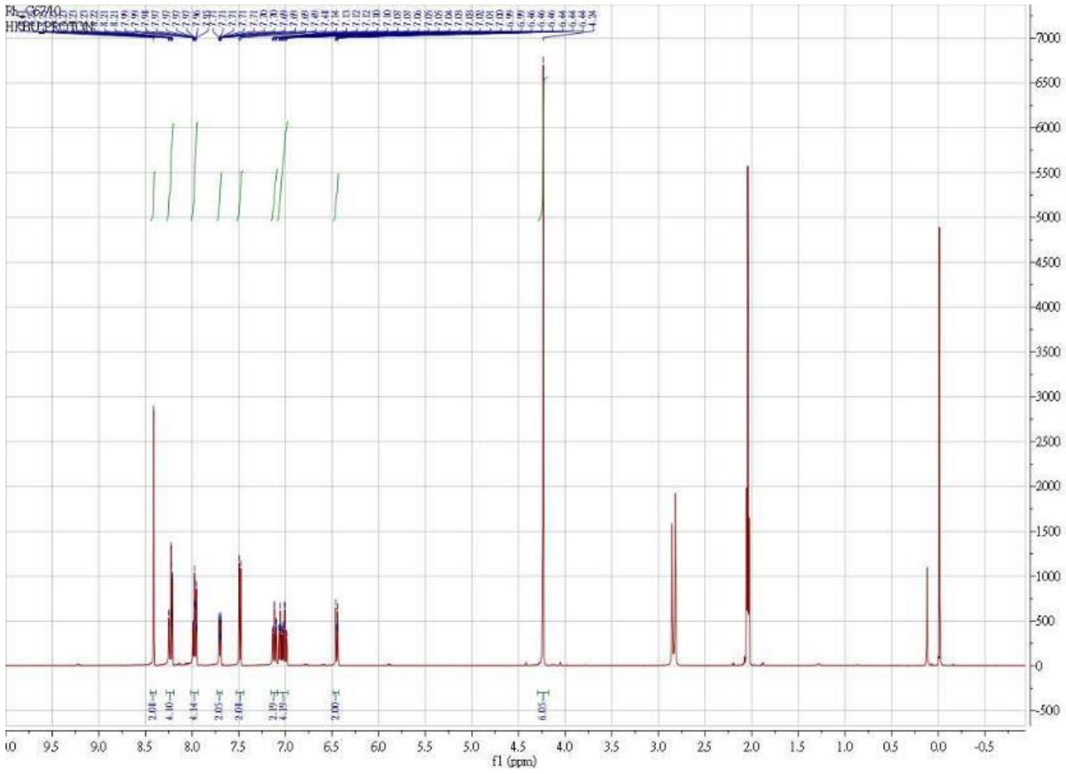

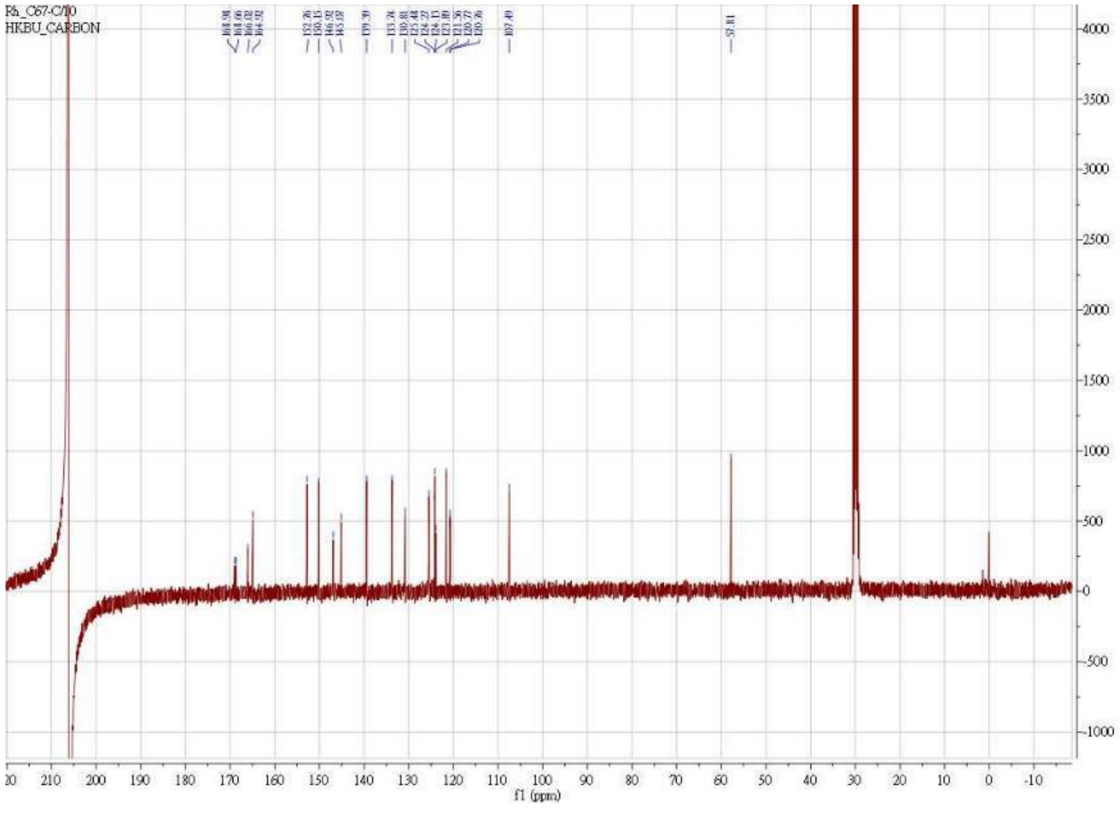
Rh_C67-C/10
HKBU_CARBON
f1 (ppm)

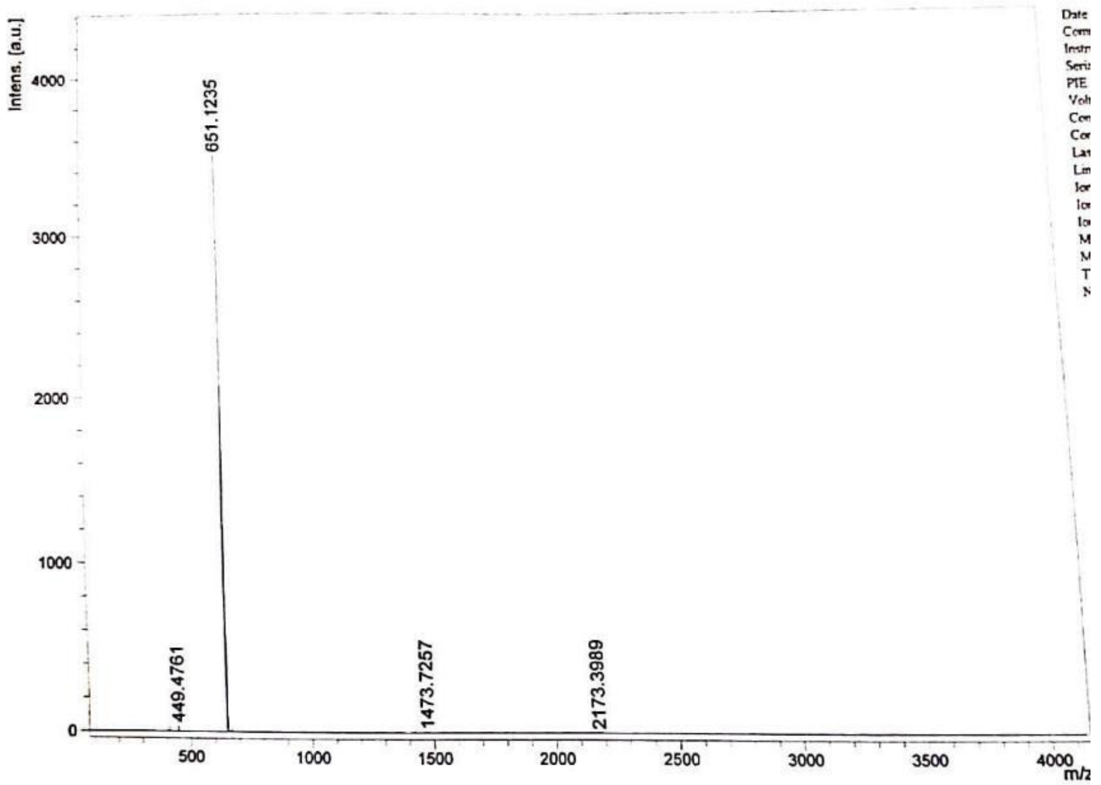
Intens. [a.u.]
651.1235
449.4761
1473.7257
2173.3989
m/z

Complex **1c**. Yield: 54%. ^{1}H NMR (400 MHz, Acetone-d_6) δ 8.43 (s, 2H), 8.32 – 8.22 (m, 4H), 7.97 – 7.87 (m, 4H), 7.76 (ddd, J = 5.8, 1.5, 0.8 Hz, 2H), 7.54 (d, J = 6.1 Hz, 2H), 7.27 (dd, J = 8.3, 2.0 Hz, 2H), 7.06 (ddd, J = 7.3, 5.8, 1.4 Hz, 2H), 6.50 (d, J = 2.0 Hz, 2H), 4.26 (s, 6H). ^{13}C NMR (100 MHz, Acetone-d_6) δ 166.87, 164.45, 152.93, 149.62, 147.40, 143.95, 139.15, 134.23, 126.88, 125.78, 125.00, 124.25, 123.95, 121.39, 120.52, 117.04, 107.24, 57.32. HRMS $[C_{36}H_{26}O_2IrN_4Br_2]^+$ calculated: 899.0021, found: 899.0053. Anal. ($C_{36}H_{26}Br_2IrN_4O_2PF_6$) C, H, N: calculated: 41.43, 2.51, 5.37; found: 41.28, 2.59, 5.41.

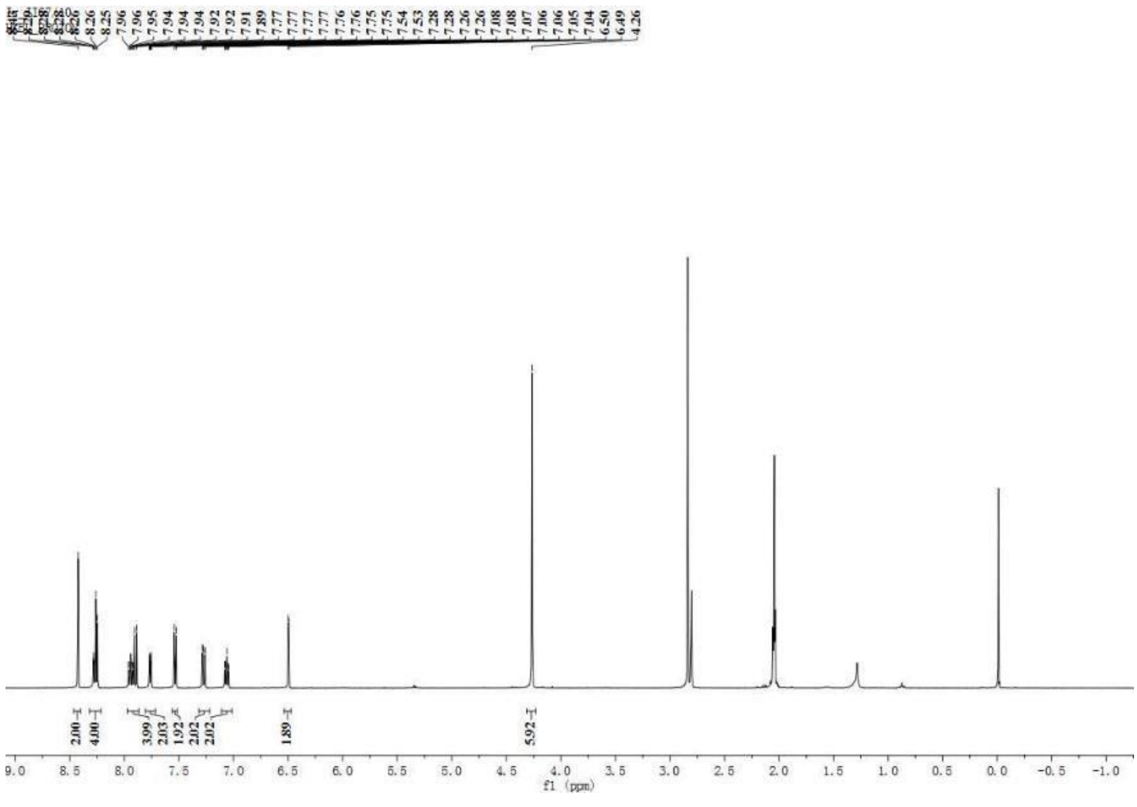

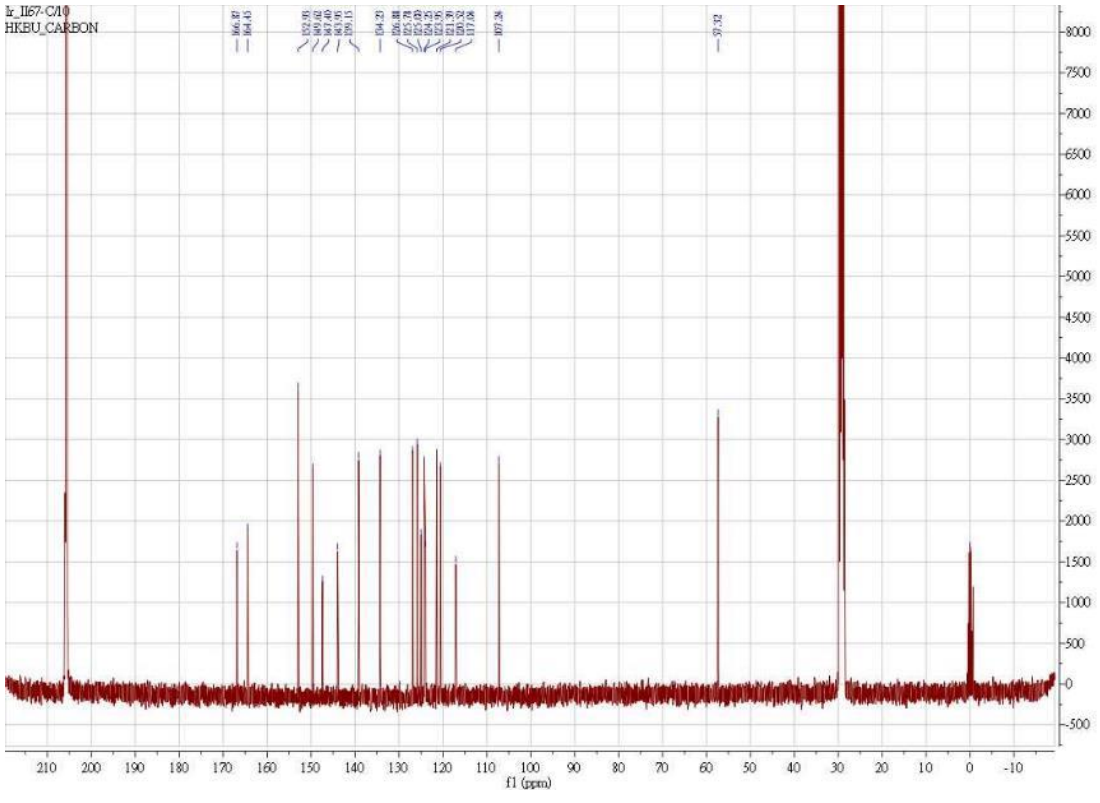
Ir_II67-C/10
HKBU_CARBON
166.87
164.45
152.93
149.62
147.40
143.95
139.15
134.23
126.88
125.78
125.03
124.25
123.95
121.39
120.32
117.08
107.28
57.32
f1 (ppm)

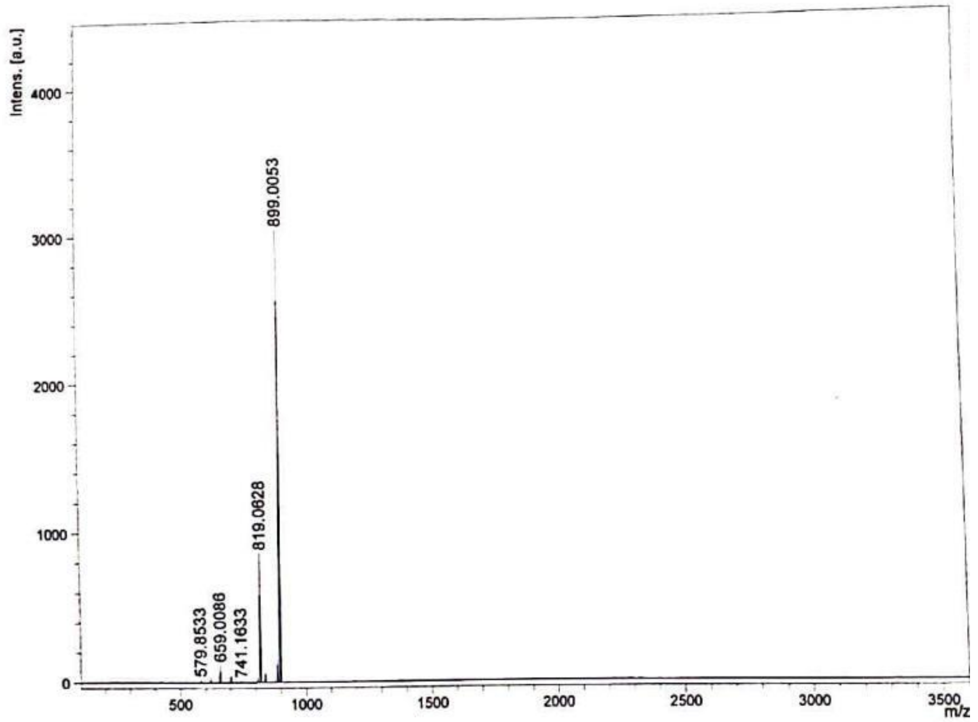
Intens. [a.u.]
899.0053
819.0828
579.8533
659.0086
741.1633
m/z

Complex **1d**. Yield: 58%. ^{1}H NMR (400 MHz, Acetone-d_6) δ 8.41 (s, 2H), 8.15 (dd, *J* = 8.1, 1.3 Hz, 2H), 8.10 (d, *J* = 6.0 Hz, 2H), 7.82 (dt, *J* = 7.7, 1.0 Hz, 2H), 7.68 (d, *J* = 5.6 Hz, 2H), 7.46 (d, *J* = 6.0 Hz, 2H), 7.14 (ddd, *J* = 8.2, 7.3, 1.4 Hz, 2H), 7.03 – 6.88 (m, 4H), 6.40 (dt, *J* = 7.5, 1.2 Hz, 2H), 4.24 (s, 6H), 2.87 (s, 6H). ^{13}C NMR (101 MHz, Acetone) δ 170.66, 170.34, 164.88, 163.89, 163.87, 152.41, 148.22, 146.89, 146.75, 143.03, 133.81, 133.80, 133.51, 129.73, 129.72, 129.30, 123.90, 123.67, 123.32, 121.54, 117.65, 107.39, 57.76, 23.26. HRMS $[C_{38}H_{32}O_2RhN_4]^+$ calculated: 679.1575, found: 679.1534. Anal. ($C_{38}H_{32}RhN_4O_2PF_6$+H_2O) C, H, N: calculated: 54.17, 4.07, 6.65; found: 54.20, 4.17, 6.65.

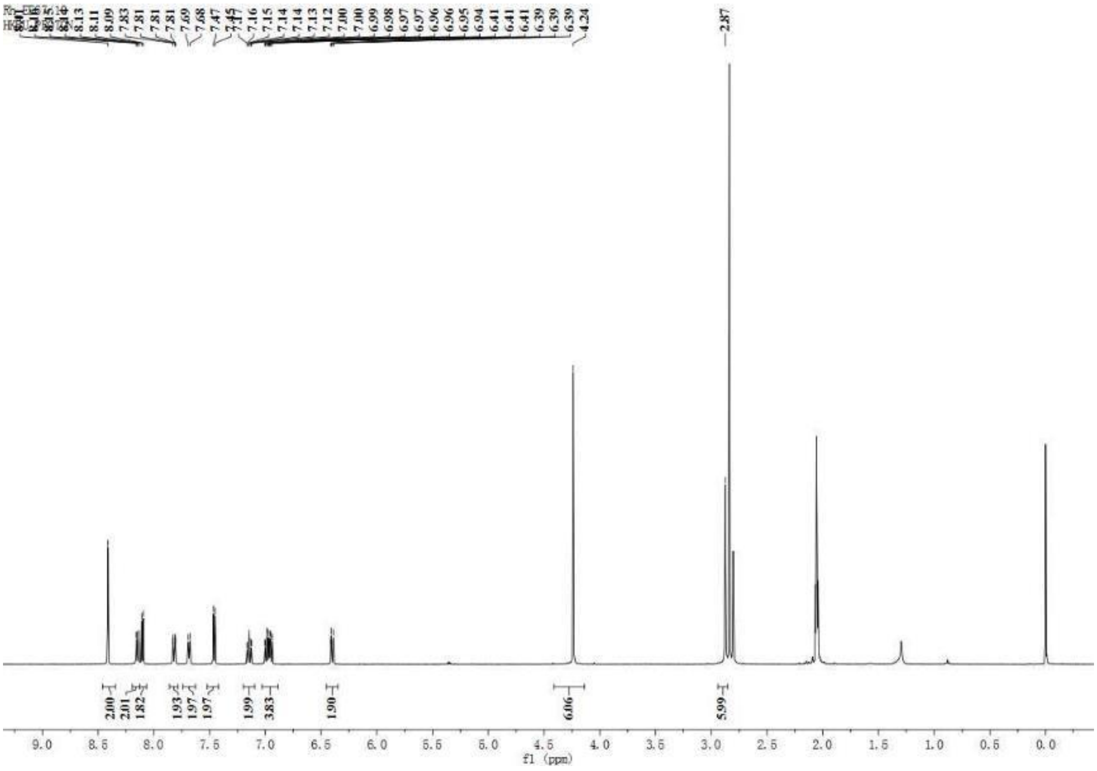

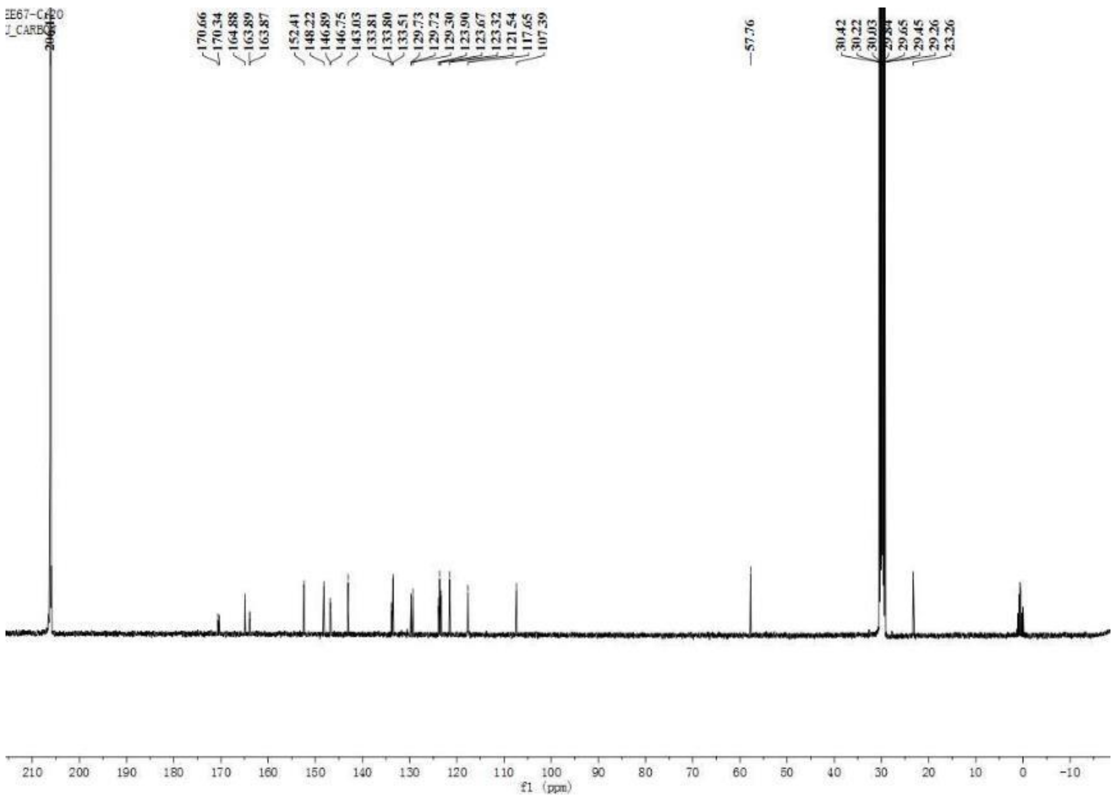
170.66
170.34
164.88
163.89
163.87
152.41
148.22
146.89
146.75
143.03
133.81
133.80
133.51
129.73
129.72
129.30
123.90
123.67
123.32
121.54
117.65
107.39
57.76
30.42
30.22
30.03
29.65
29.45
29.26
23.26
210 200 190 180 170 160 150 140 130 120 110 100 90 80 70 60 50 40 30 20 10 0 -10
f1 (ppm)

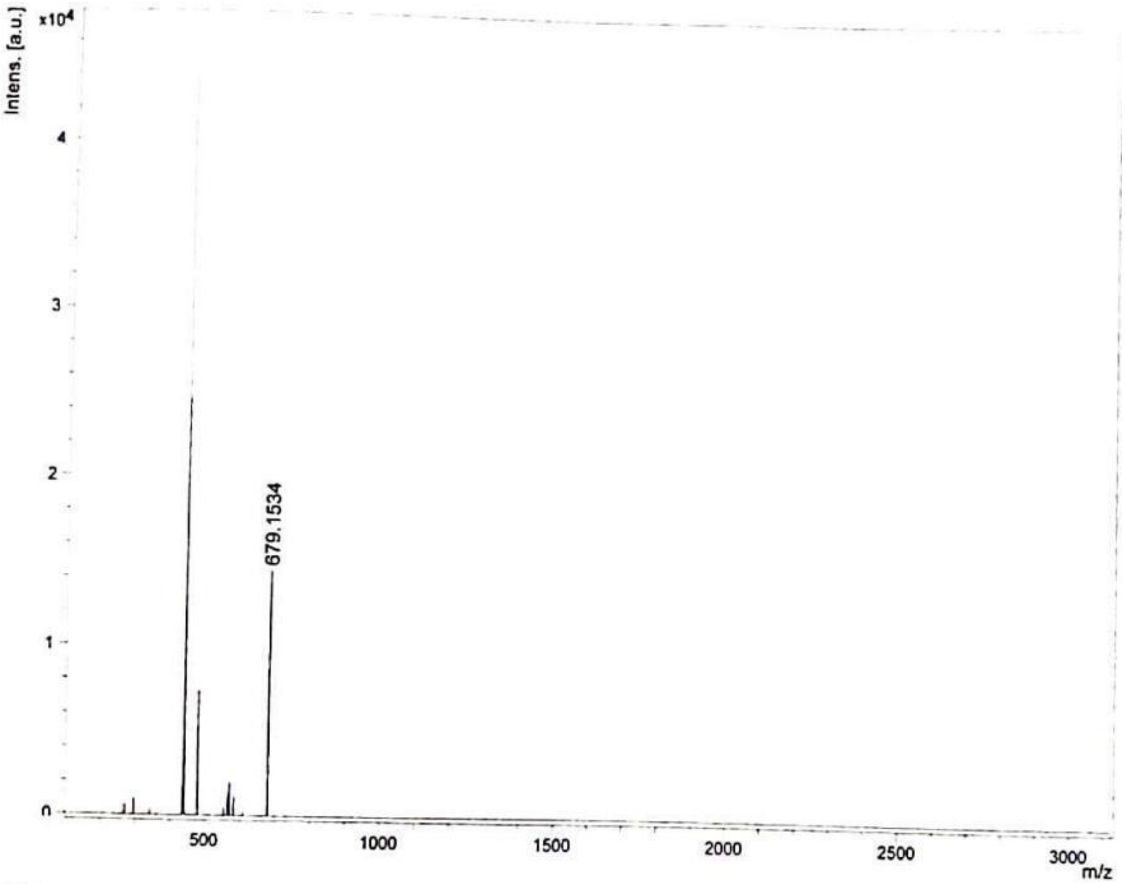
Intens. [a.u.]
x10^4
4
3
2
1
0
679.1534
500
1000
1500
2000
2500
3000
m/z

Complex **1e**. Yield: 63%. ^{1}H NMR (400 MHz, Acetone-d_6) δ 8.42 (s, 2H), 8.11 (dd, J = 8.2, 1.3 Hz, 2H), 8.06 (d, J = 6.1 Hz, 2H), 7.73 (d, J = 6.1 Hz, 4H), 7.49 (d, J = 6.1 Hz, 2H), 7.07 (ddd, J = 8.2, 7.2, 1.4 Hz, 2H), 6.93 – 6.84 (m, 4H), 6.42 (dd, J = 7.6, 1.4 Hz, 2H), 4.25 (s, 6H), 2.88 (s, 6H). ^{13}C NMR (100 MHz, Acetone-d_6) δ 170.66, 170.34, 164.88, 163.89, 163.87, 152.41, 148.22, 146.89, 146.75, 143.03, 133.81, 133.80, 133.51, 129.73, 129.72, 129.30, 123.90, 123.67, 123.32, 121.54, 117.65, 107.39, 57.76, 23.26. HRMS $[C_{38}H_{32}IrN_4O_2]^+$ calculated: 769.2155, found: 769.2194. Anal. ($C_{38}H_{32}O_2IrN_4PF_6$+$2H_2O$) C, H, N: calculated: 48.05, 3.82, 5.90; found: 47.96, 3.54, 6.08.

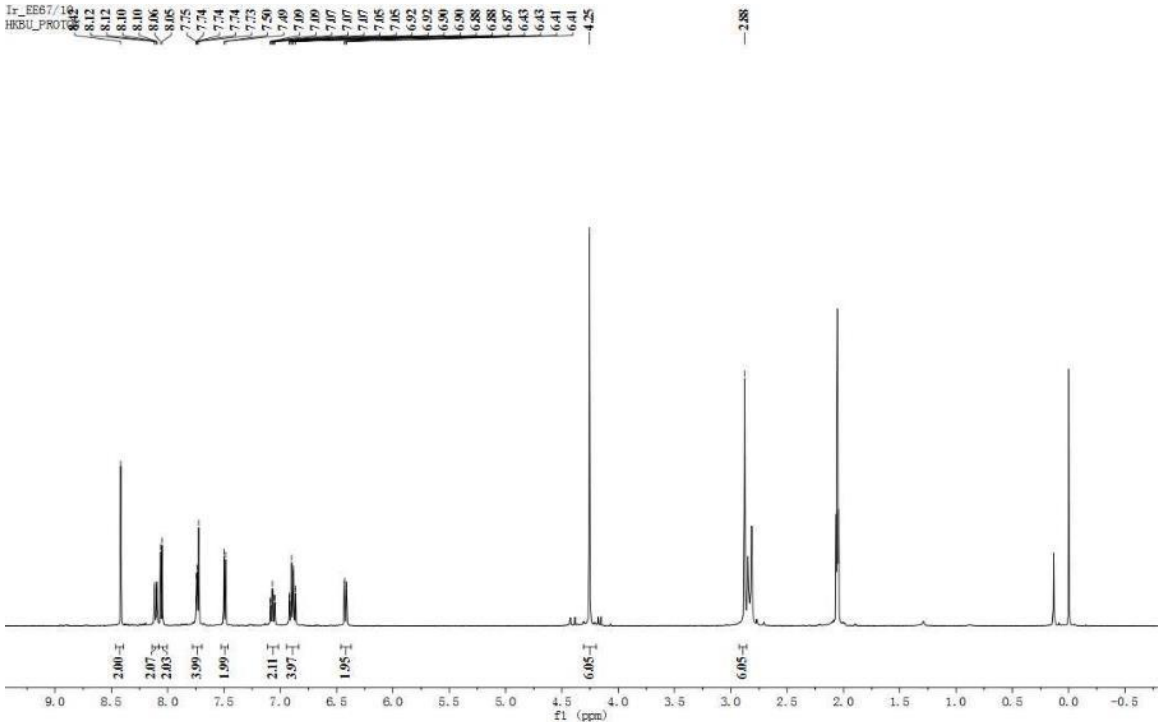

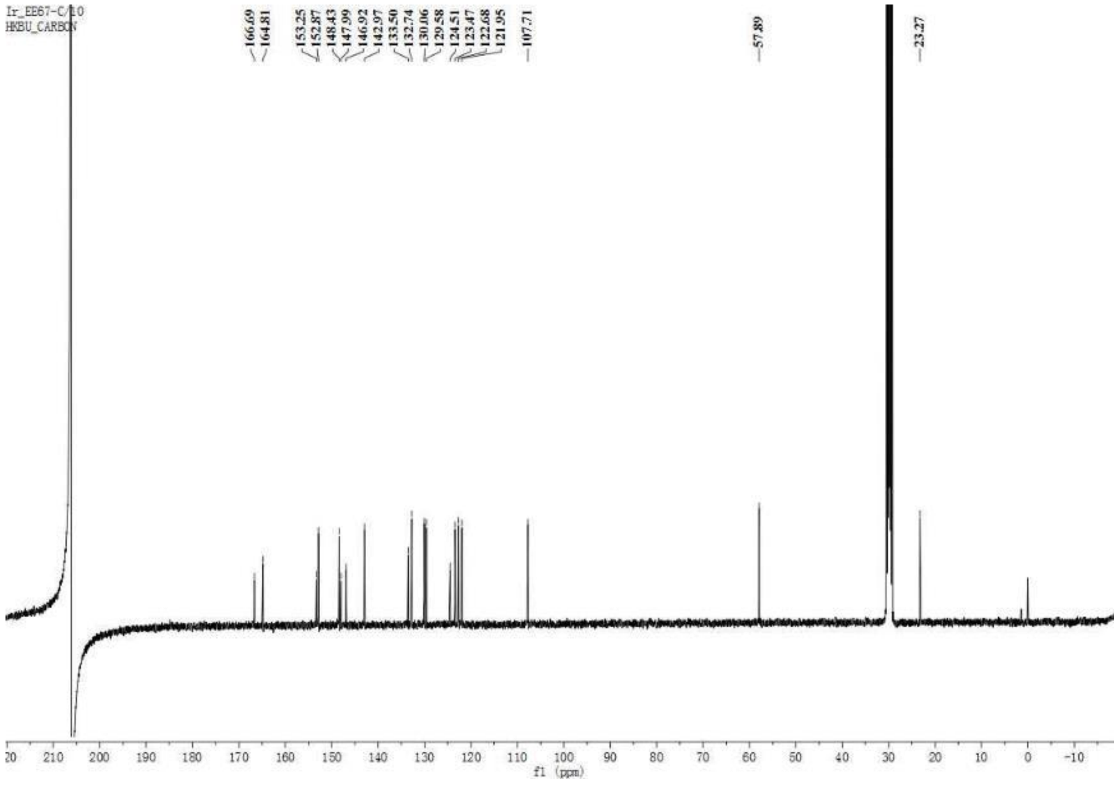
Ir_EE67-C/10
HKBU_CARBON
166.69
164.81
153.25
152.87
148.43
147.99
146.92
142.97
133.50
132.74
130.06
129.58
124.51
123.47
122.68
121.95
107.71
57.89
23.27
f1 (ppm)

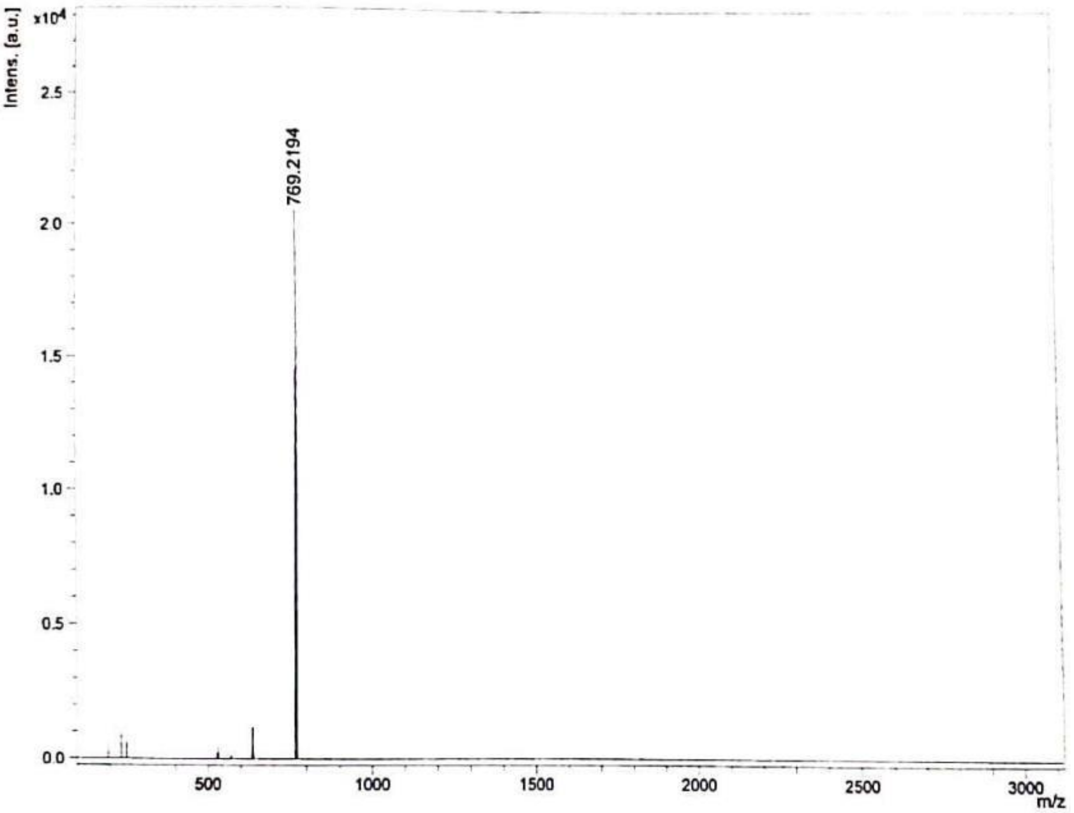
Intens. [a.u.]
x10⁴
769.2194
m/z
Acquisition method name D:\Methods\flexControlMethods\20140609_john\RP_PepMix\RP_PepMix 1-4-2014_from David_LOM.par
Sample name (file name prefix) Ir_EE67-DCTB\0_M19\1

Complex **1f**. Yield: 63% ^{1}H NMR (400 MHz, Acetone-d_6) δ 8.40 (s, 2H), 8.22 (dd, *J* = 6.0, 0.5 Hz, 2H), 8.16 (dt, *J* = 8.2, 1.1 Hz, 2H), 7.97 – 7.88 (m, 2H), 7.85 (d, *J* = 7.9 Hz, 2H), 7.65 (dt, *J* = 5.8, 0.8 Hz, 2H), 7.48 (d, *J* = 6.0 Hz, 2H), 6.99 (s, 2H), 6.94 (ddd, *J* = 7.9, 1.6, 0.8 Hz, 2H), 6.28 (dd, *J* = 1.8, 0.9 Hz, 2H), 4.23 (s, 6H), 2.10 (s, 6H). ^{13}C NMR (101 MHz, Acetone) δ 169.20, 168.88, 166.10, 166.08, 164.90, 152.74, 149.97, 146.92, 142.42, 140.63, 140.62, 139.21, 134.43, 125.33, 125.15, 123.86, 123.68, 121.54, 120.38, 120.36, 107.45, 57.78, 21.97. HRMS $[C_{38}H_{32}O_2RhN_4]^+$ calculated: 679.1575, found: 679.1541. Anal. ($C_{38}H_{32}RhN_4O_2PF_6$) C, H, N: calculated: 49.94, 3.53, 6.13; found: 49.47, 3.48, 6.07.

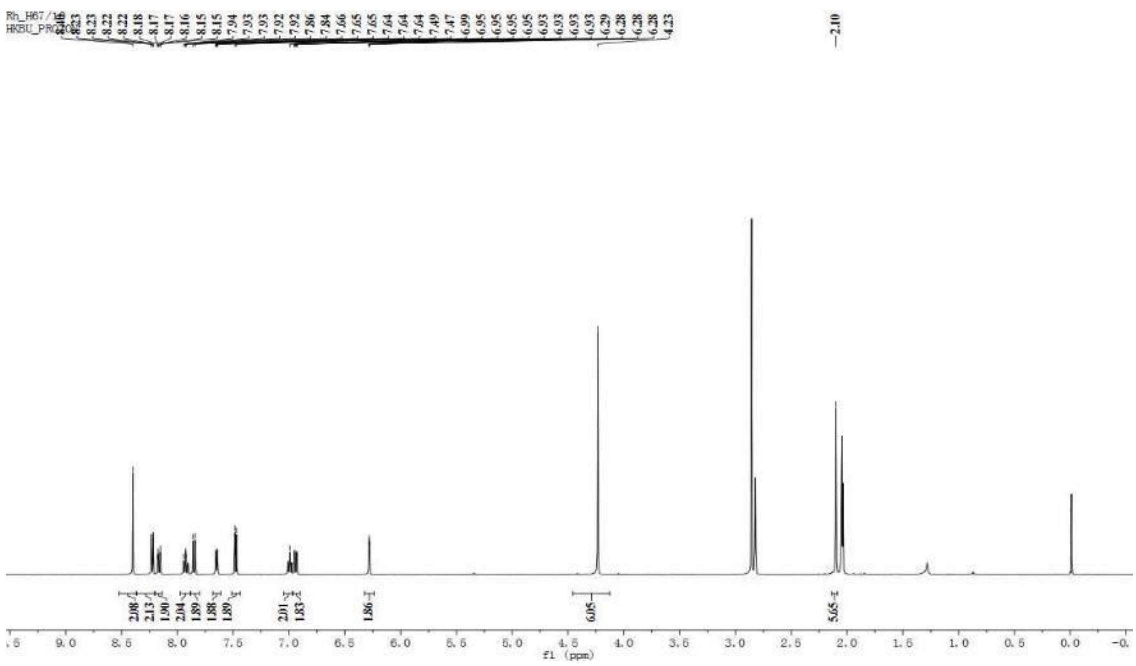

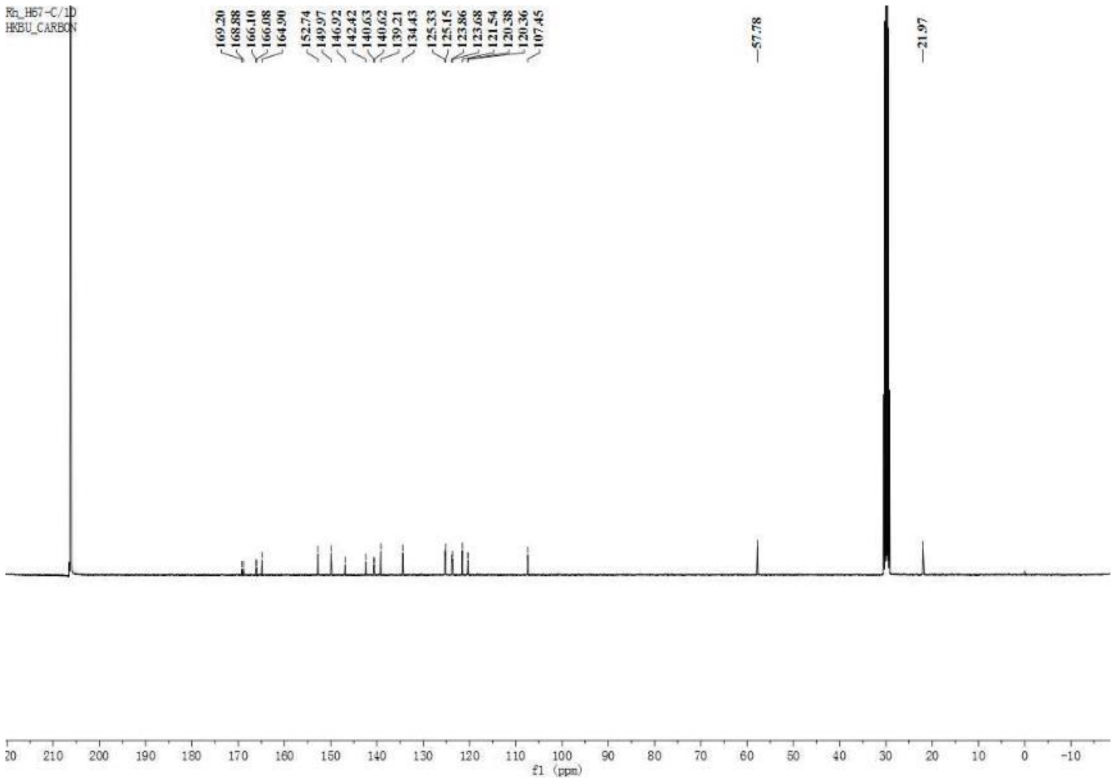
Rh_H67-C/1D
HKBU_CARBON
169.20
168.88
166.10
166.08
164.90
152.74
149.97
146.92
142.42
140.63
140.62
139.21
134.43
125.33
125.15
123.86
123.68
121.54
120.38
120.36
107.45
57.78
21.97
f1 (ppm)

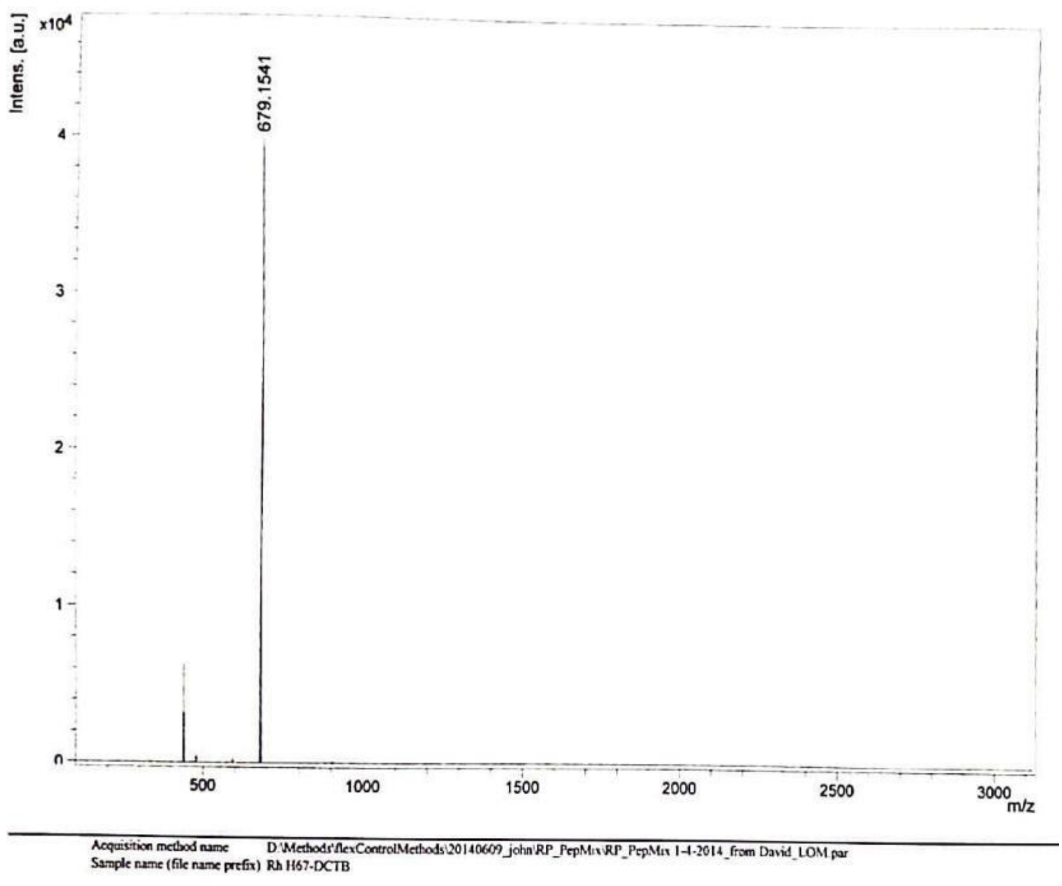
Intens. [a.u.]
x10^4
679.1541
m/z
Acquisition method name D:\Methods\flexControlMethods\20140609_john\RP_PepMix\RP_PepMix 1-4-2014_from David_LOM.par
Sample name (file name prefix) Rh H67-DCTB

Complex **1g**. Yield: 61%.^{1}H NMR (400 MHz, Acetone-*d*$_6$) δ 8.40 (s, 2H), 8.20 – 8.11 (m, 4H), 7.84 (ddd, *J* = 8.2, 7.4, 1.5 Hz, 2H), 7.79 (d, *J* = 8.0 Hz, 2H), 7.69 (ddd, *J* = 5.9, 1.6, 0.8 Hz, 2H), 7.50 (d, *J* = 6.1 Hz, 2H), 6.97 – 6.85 (m, 4H), 6.31 – 6.24 (m, 2H), 4.25 (s, 6H), 2.11 (s, 6H). ^{13}C NMR (100 MHz, Acetone-*d*$_6$) δ 168.86, 164.77, 153.22, 151.80, 149.85, 148.14, 142.63, 140.84, 139.00, 133.48, 125.62, 124.41, 124.11, 123.56, 121.90, 120.11, 107.71, 57.86, 21.83. HRMS [$C_{38}H_{32}IrN_4O_2$]$^+$ calculated: 769.2155, found: 769.2197. Anal. ($C_{38}H_{32}O_2IrN_4PF_6$) C, H, N: calculated: 49.94, 3.53, 6.13; found: 49.47, 3.48, 6.07.

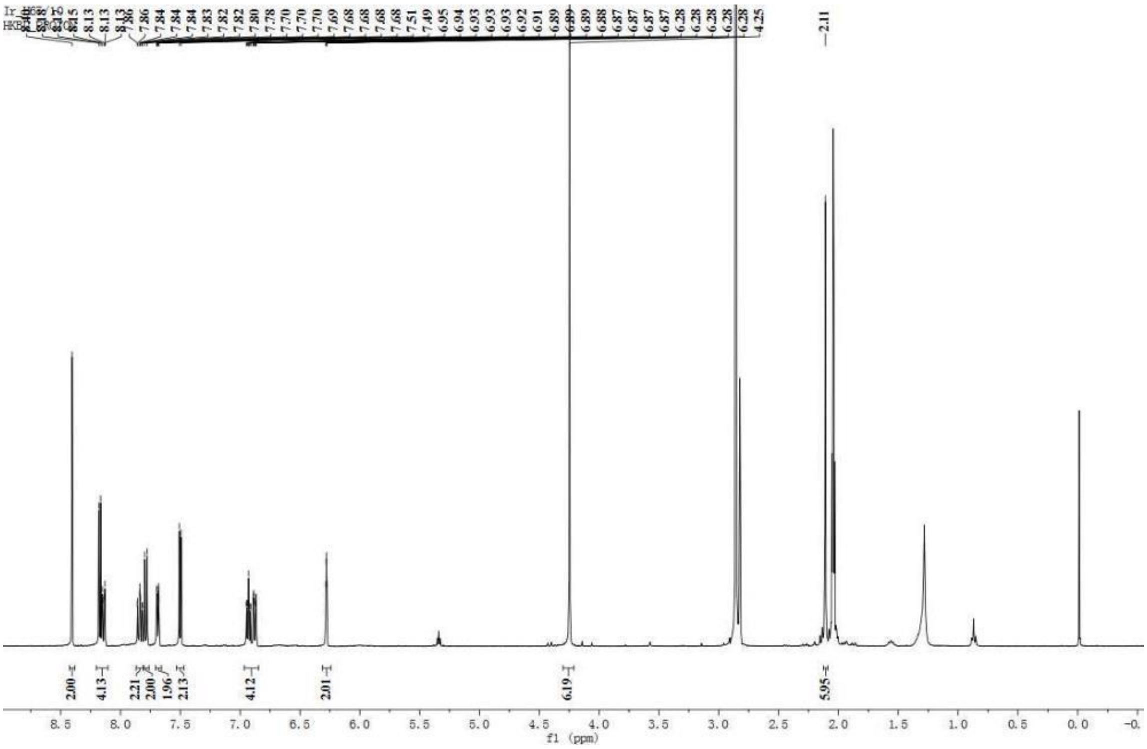

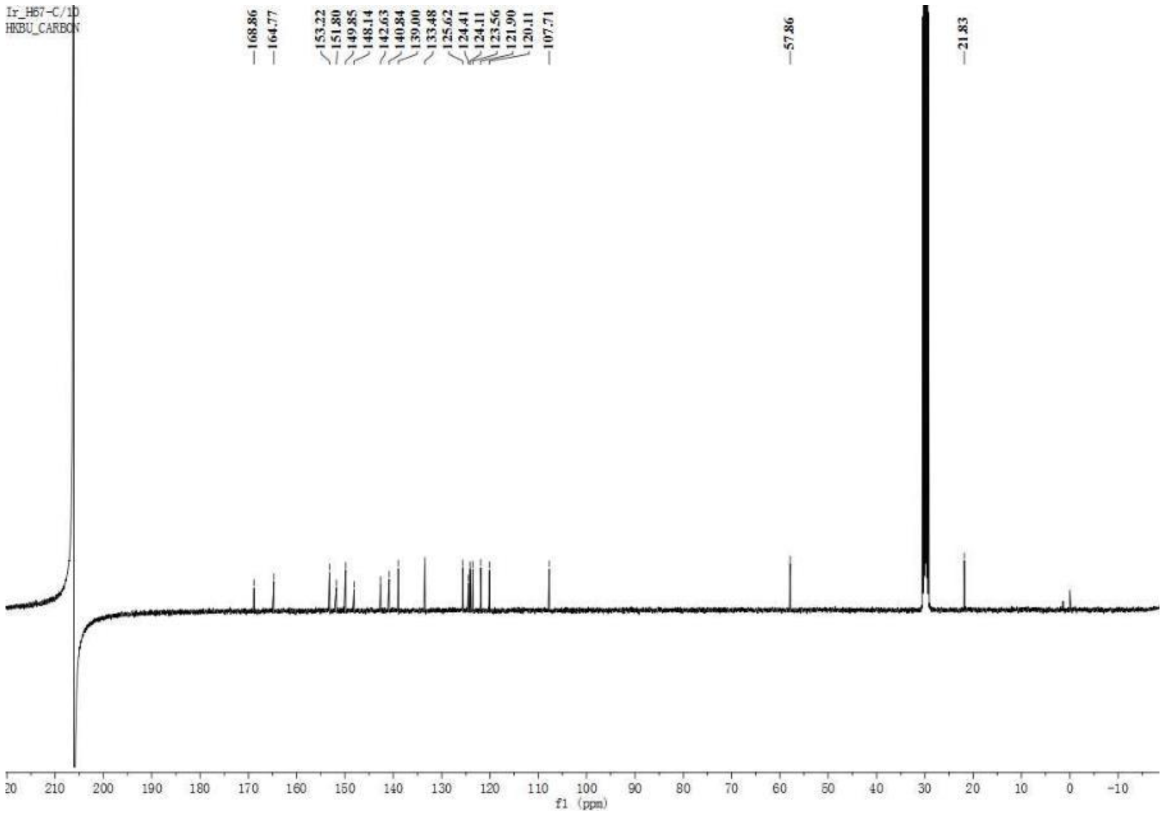
Ir_H67-C/10
HKBU_CARBON
168.86
164.77
153.22
151.80
149.85
148.14
142.63
140.84
139.00
133.48
125.62
124.41
124.11
123.56
121.90
120.11
107.71
57.86
21.83
f1 (ppm)

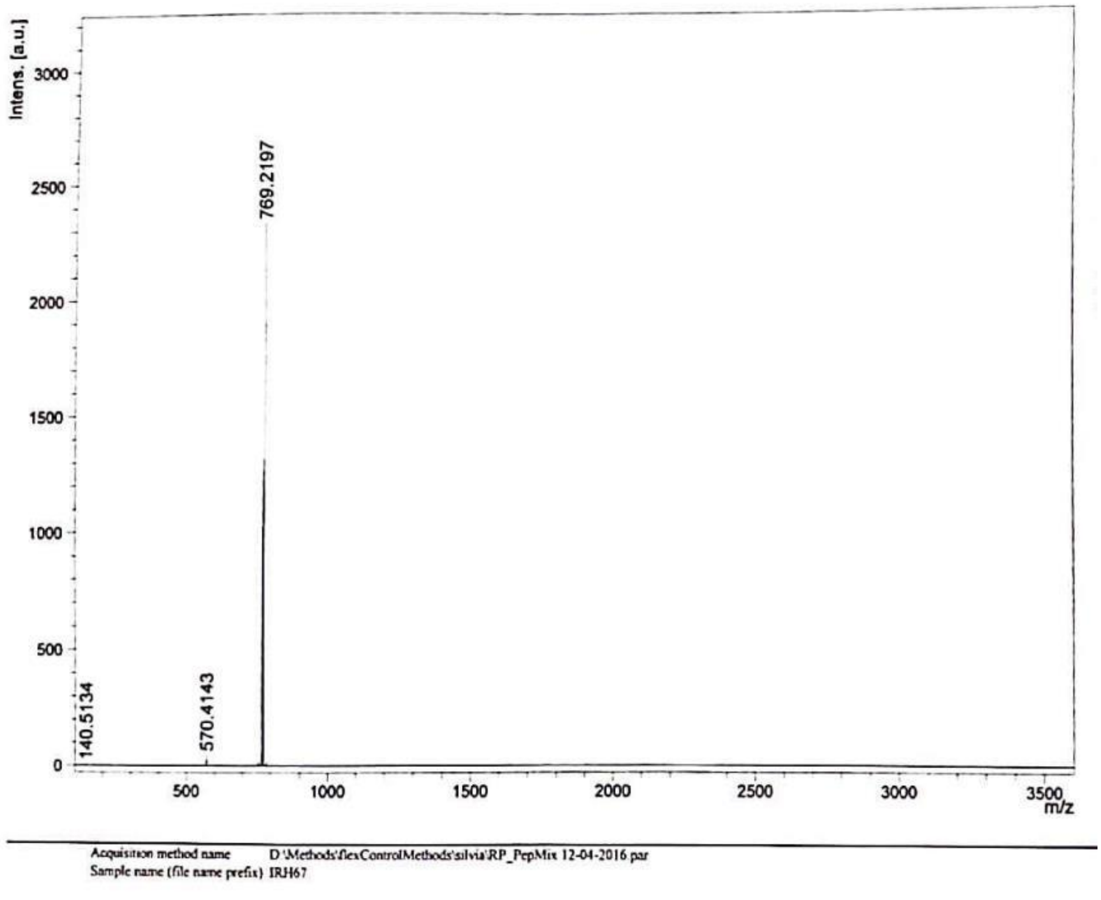
Intens. [a.u.]
769.2197
570.4143
140.5134
m/z
Acquisition method name D:\Methods\flexControlMethods\silvia\RP_PepMix 12-04-2016.par
Sample name (file name prefix) IRH67

Complex **1h**. Yield: 51% ^{1}H NMR (400 MHz, Acetone-d_6) δ 8.38 (d, J = 2.7 Hz, 2H), 8.29 (dt, J = 8.3, 1.2 Hz, 2H), 8.02 (td, J = 7.8, 1.5 Hz, 2H), 7.93 – 7.86 (m, 6H), 7.29 – 7.22 (m, 6H), 6.40 (d, J = 2.0 Hz, 2H), 4.08 (s, 6H). ^{13}C NMR (100 MHz, Acetone-d_6) δ 169.12, 167.51, 158.28, 153.96, 152.56, 150.20, 144.36, 139.93, 134.70, 127.62, 126.43, 125.72, 125.08, 121.30, 115.09, 112.54, 57.38. HRMS $[C_{34}H_{26}O_2IrN_4Br_2]^+$ calculated: 875.0031, found: 875.0041. Anal. ($C_{34}H_{26}Br_2IrN_4O_2PF_6$) C, H, N: calculated: 40.05, 2.57, 5.50; found: 40.88, 2.77, 5.59.

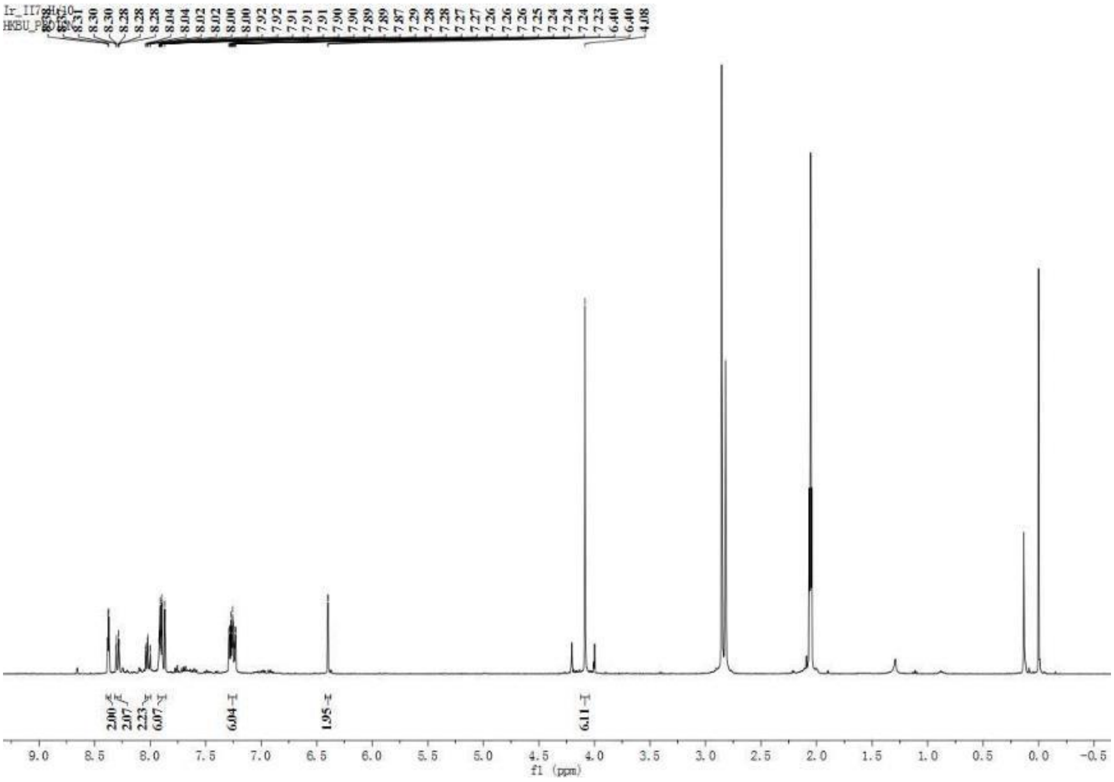

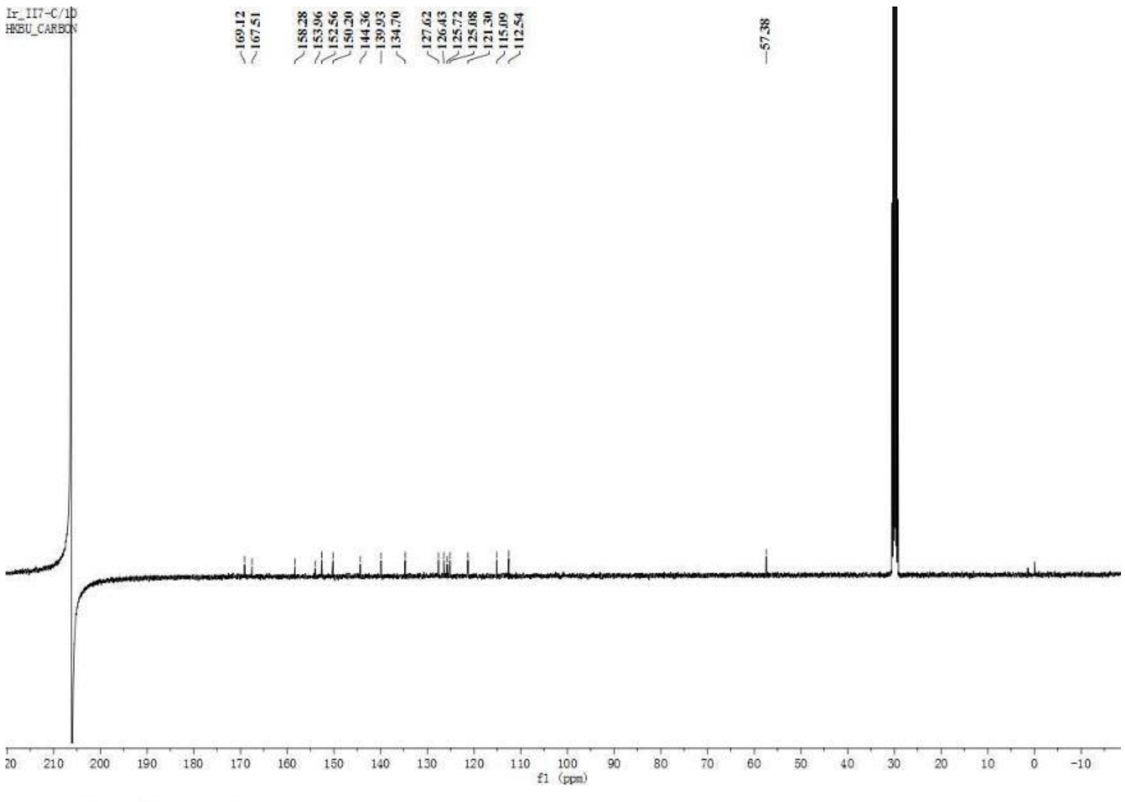
Ir_II7-C/10
HKBU_CARBON
169.12
167.51
158.28
153.96
152.56
150.20
144.36
139.93
134.70
127.62
126.43
125.72
125.08
121.30
115.09
112.54
57.38
f1 (ppm)

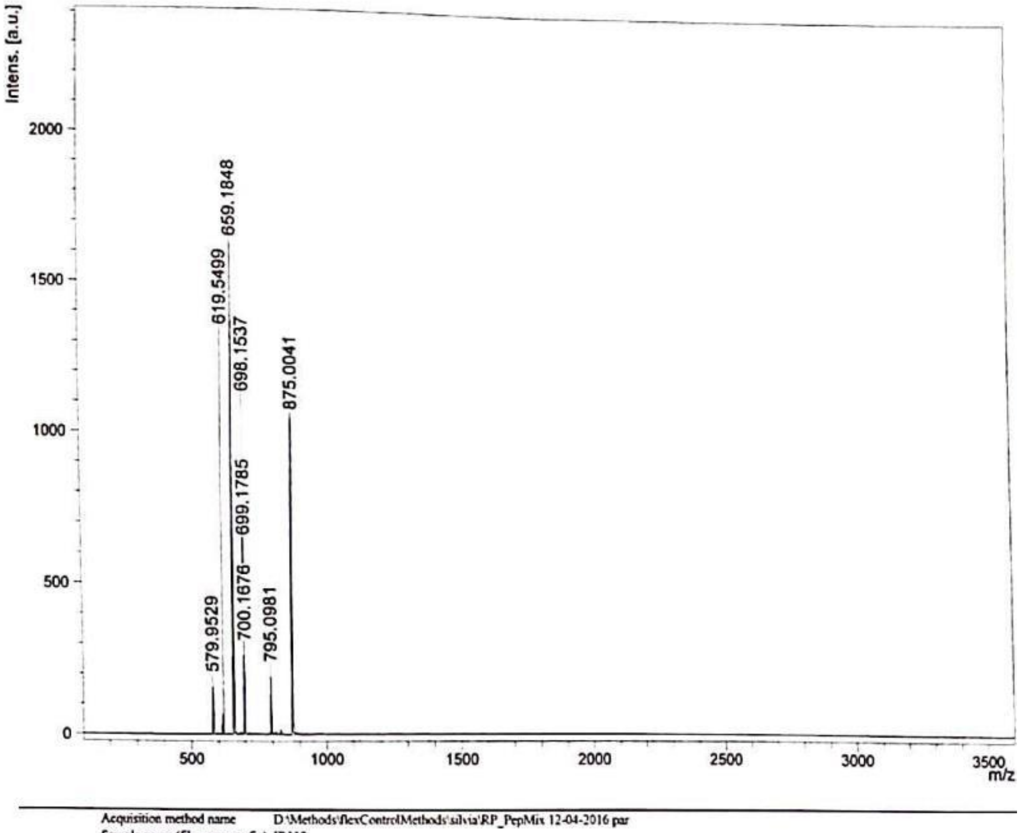
Intens. [a.u.]
579.9529
619.5499
659.1848
698.1537
699.1785
700.1676
795.0981
875.0041
m/z
Acquisition method name D:\Methods\flexControlMethods\silvia\RP_PepMix 12-04-2016 par
Sample name (file name prefix) IR117

Complex **1i**. Yield: 64%. ^{1}H NMR (400 MHz, Acetone-d_6) δ 8.95 (dd, J = 8.3, 1.5 Hz, 2H), 8.57 (ddd, J = 4.9, 1.5, 0.5 Hz, 2H), 8.41 (s, 2H), 8.32 (dt, J = 8.2, 1.1 Hz, 2H), 8.12 – 7.96 (m, 6H), 7.67 (ddt, J = 5.8, 1.6, 0.9 Hz, 2H), 7.37 (dd, J = 8.3, 2.0 Hz, 2H), 7.11 (ddd, J = 7.3, 5.7, 1.4 Hz, 2H), 6.52 (dd, J = 2.0, 1.1 Hz, 2H). ^{13}C NMR (100 MHz, Acetone-d_6) δ 169.07, 164.10, 151.19, 149.75, 145.58, 143.39, 139.43, 139.39, 135.02, 131.45, 128.41, 127.06, 127.01, 126.64, 124.66, 124.64, 124.44, 120.84, 116.99. HRMS $[C_{34}H_{22}RhN_4Br_2]^+$ calculated: 748.9246, found: 748.7485. Anal. ($C_{34}H_{22}Br_2RhN_4PF_6$) C, H, N: calculated: 45.67, 2.48, 6.27; found: 45.47, 2.45, 6.21.

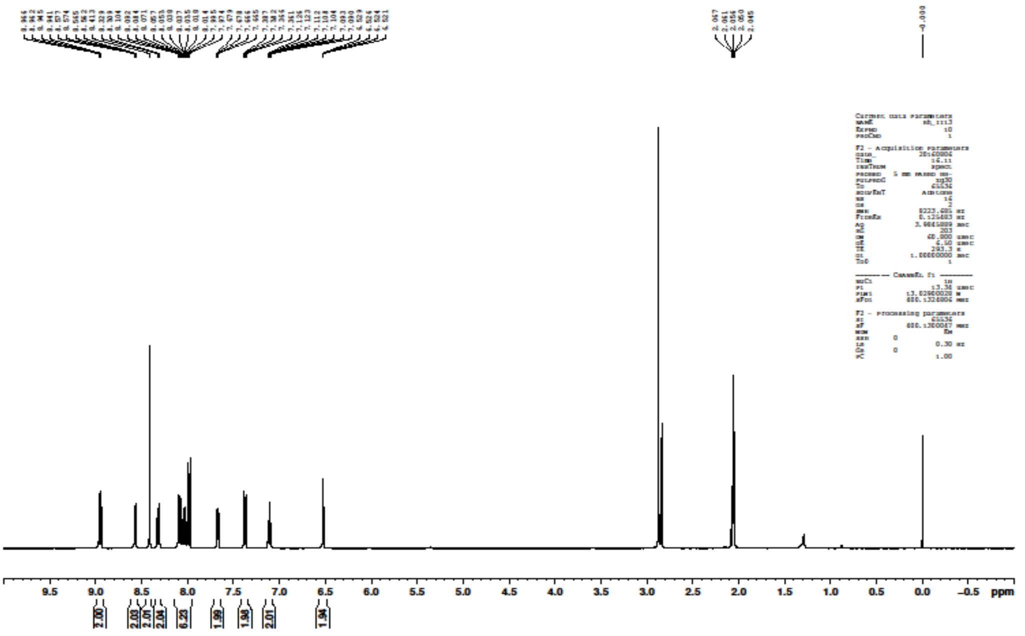

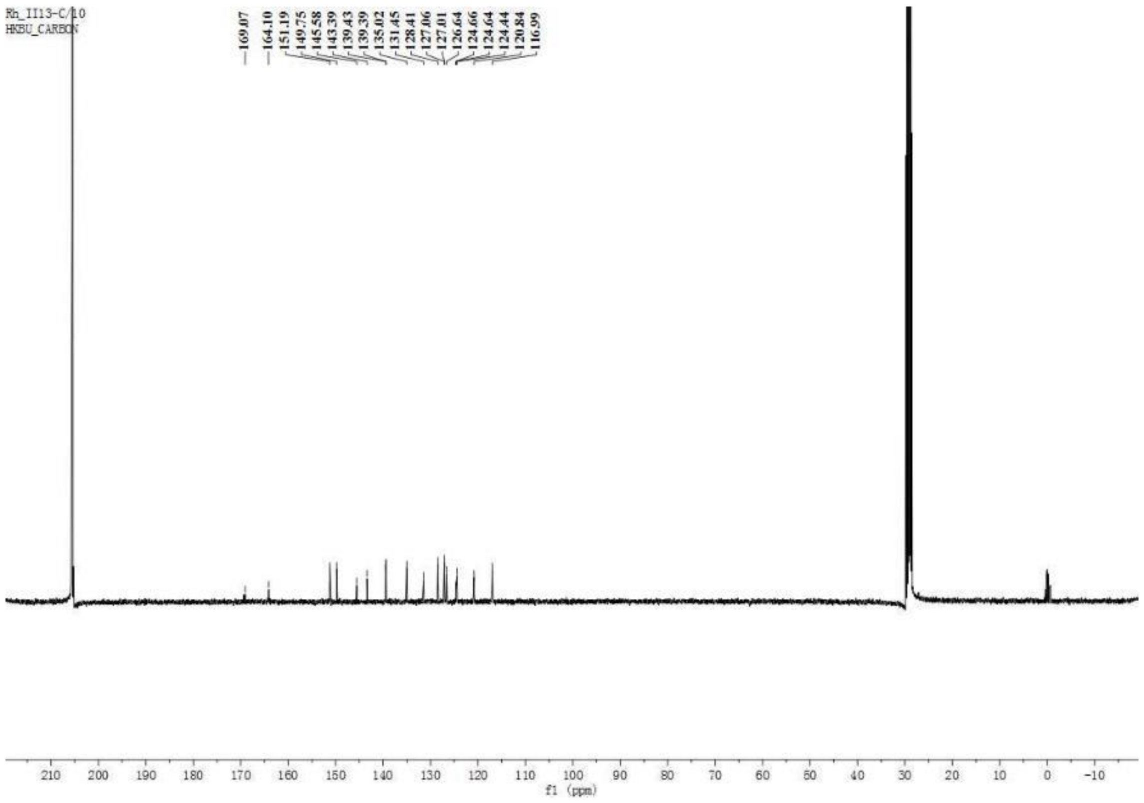
Rh_II13-C/10
HKBU_CARBON
169.07
164.10
151.19
149.75
145.58
143.39
139.43
139.39
135.02
131.45
128.41
127.06
127.01
126.64
124.66
124.64
124.44
120.84
116.99
210 200 190 180 170 160 150 140 130 120 110 100 90 80 70 60 50 40 30 20 10 0 -10
f1 (ppm)

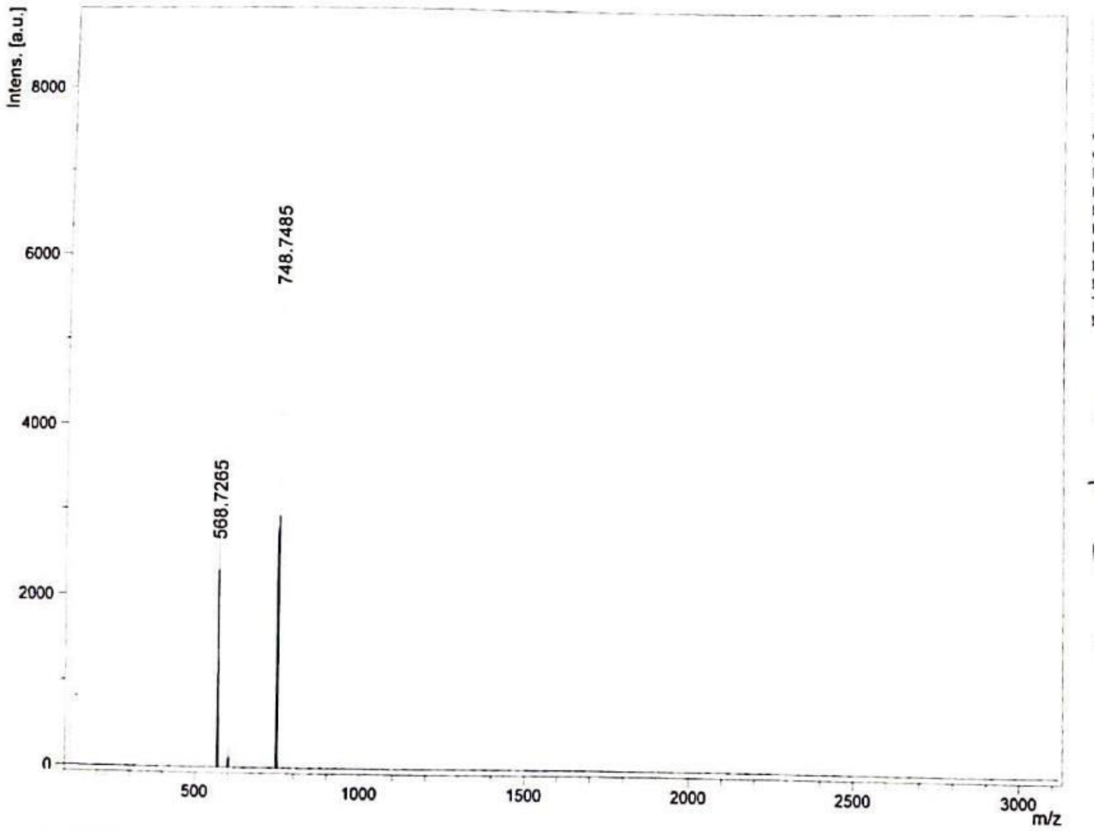
Intens. [a.u.]
8000
6000
4000
2000
0
748.7485
588.7265
500 1000 1500 2000 2500 3000
m/z
Acquisition method name D:\Methods\flexControlMethods\20140609_john\RP_PepMix\RP_PepMix 1-4-2014_from David_LOM par
Sample name (file name prefix) RH II 13-DCTB

Complex **1j**. Yield: 64%.^{1}H NMR (400 MHz, Acetone-d_6) δ 8.95 (dd, J = 8.3, 1.4 Hz, 2H), 8.53 (dd, J = 5.1, 1.4 Hz, 2H), 8.44 (s, 2H), 8.33 – 8.27 (m, 2H), 8.11 (dd, J = 8.3, 5.0 Hz, 2H), 8.00 – 7.90 (m, 4H), 7.70 (ddd, J = 5.8, 1.6, 0.8 Hz, 2H), 7.31 (dd, J = 8.3, 2.0 Hz, 2H), 7.05 (ddd, J = 7.4, 5.8, 1.4 Hz, 2H), 6.51 (d, J = 2.0 Hz, 2H). ^{13}C NMR (100 MHz, Acetone-d_6) δ 167.40, 152.86, 152.51, 150.54, 147.67, 144.52, 140.05, 140.03, 134.84, 132.72, 129.45, 128.10, 127.64, 126.78, 125.75, 125.06, 121.35. HRMS $[C_{34}H_{22}IrN_4Br_2]^+$ calculated: 839.9897, found: 839.9859. Anal. ($C_{34}H_{22}Br_2IrN_4PF_6$) C, H, N: calculated: 41.52, 2.25, 5.70; found: 41.72, 2.50, 5.66.

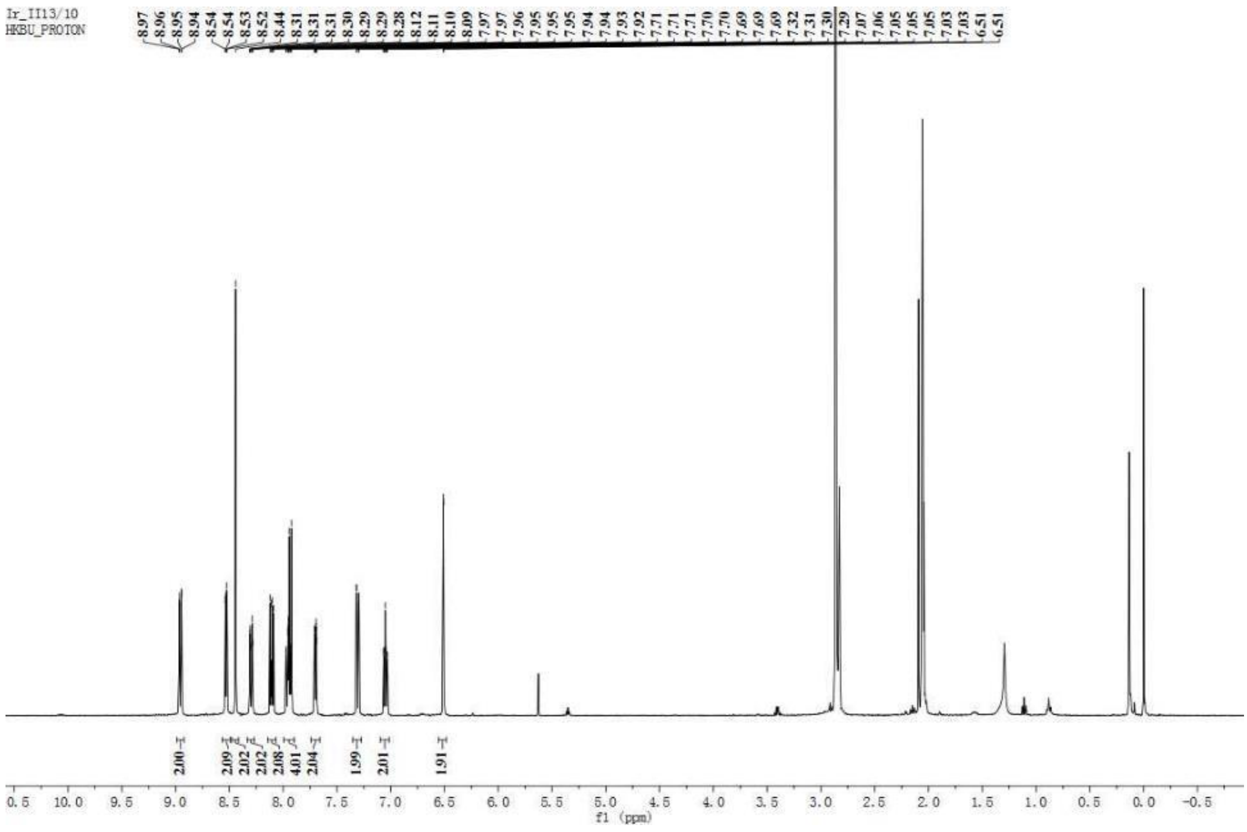

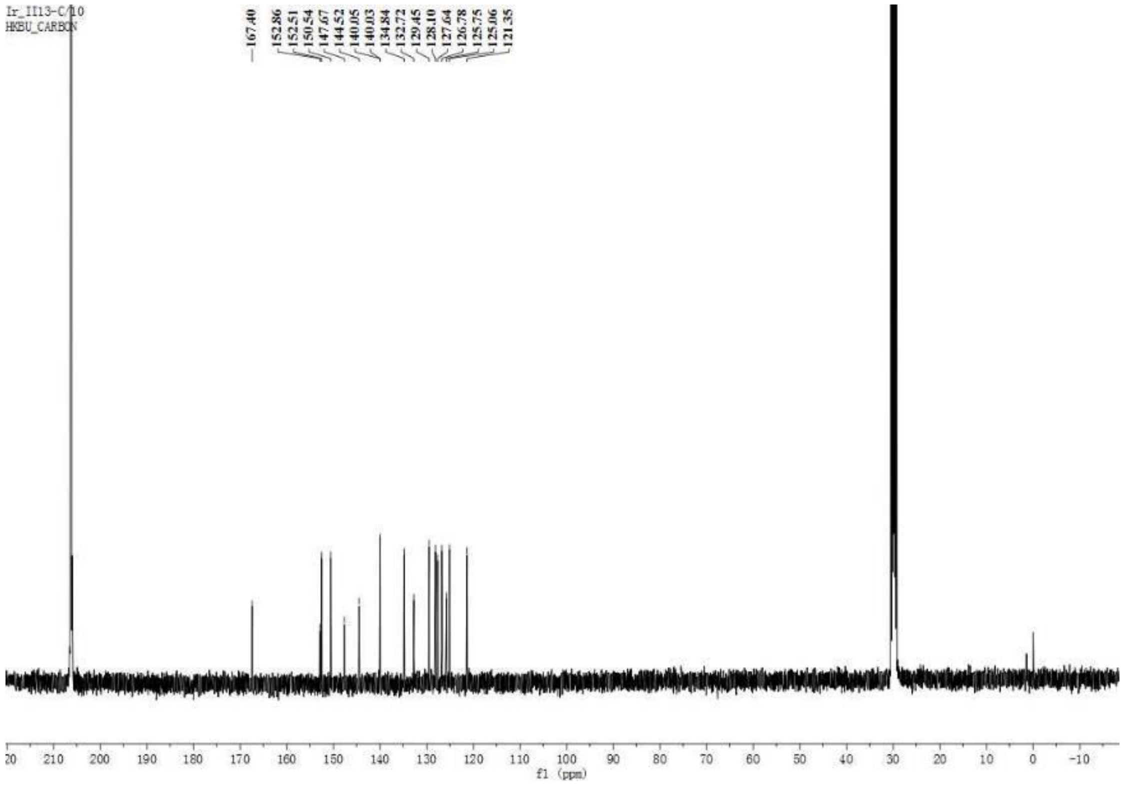

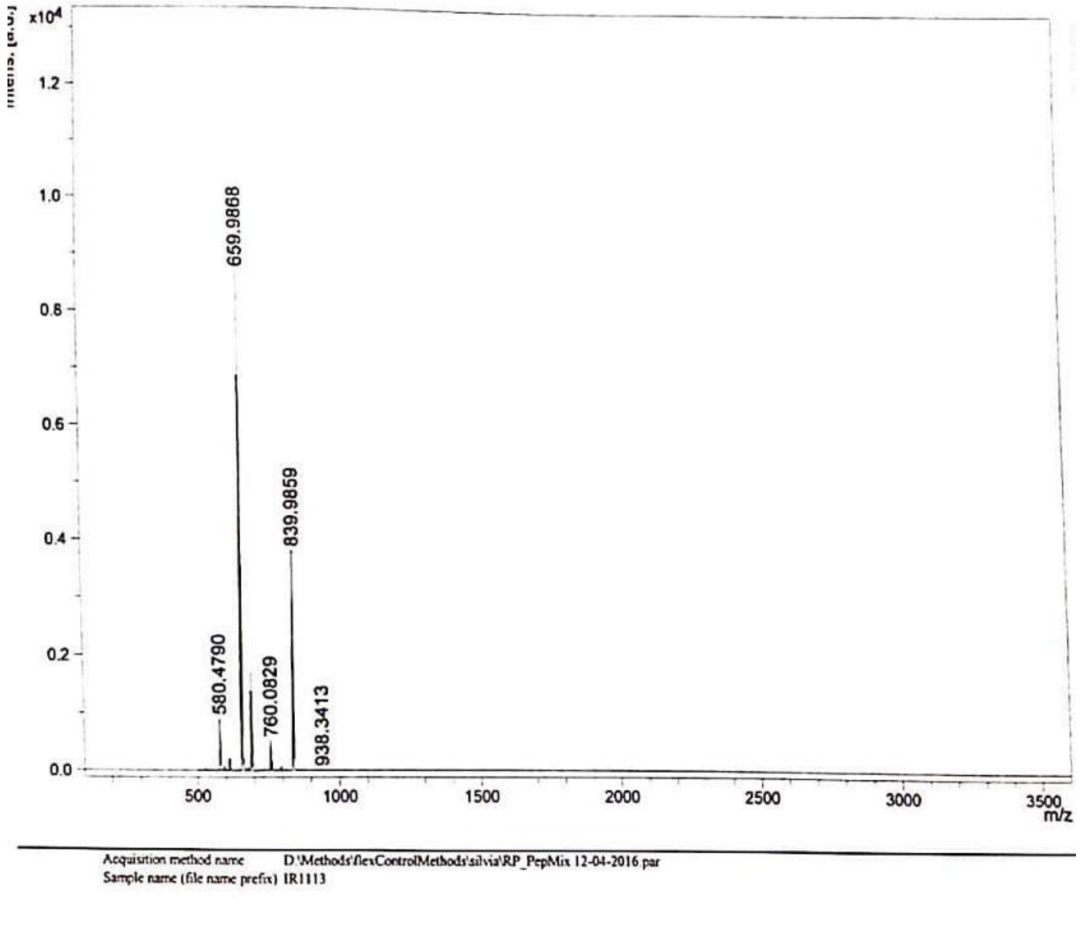

Acquisition method name D:\Methods\flexControlMethods\silvia\RP_PepMix 12-04-2016 par
Sample name (file name prefix) IR1113

Complex **1k**. Yield: 61%. ^{1}H NMR (400 MHz, Acetone-d_6) δ 8.67 (s, 2H), 8.45 (s, 4H), 8.31 (dt, J = 8.2, 1.0 Hz, 2H), 8.13 – 8.00 (m, 2H), 7.97 (d, J = 8.3 Hz, 2H), 7.78 (ddt, J = 5.8, 1.7, 0.9 Hz, 2H), 7.37 (dd, J = 8.3, 2.0 Hz, 2H), 7.09 (ddd, J = 7.3, 5.8, 1.4 Hz, 2H), 6.48 (dd, J = 2.0, 1.2 Hz, 2H). ^{13}C NMR (100 MHz, Acetone-d_6) δ 164.02, 151.68, 150.14, 146.17, 143.45, 139.60, 137.15, 135.05, 131.54, 131.37, 128.19, 127.27, 126.75, 124.55, 120.93, 116.99. HRMS $[C_{34}H_{20}RhN_4Br_4]^+$ calculated: 906.7436, found: 906.7450. Anal. ($C_{34}H_{20}Br_4RhN_4PF_6$) C, H, N: calculated: 38.82, 1.92, 5.33; found: 38.97, 2.05, 5.34.

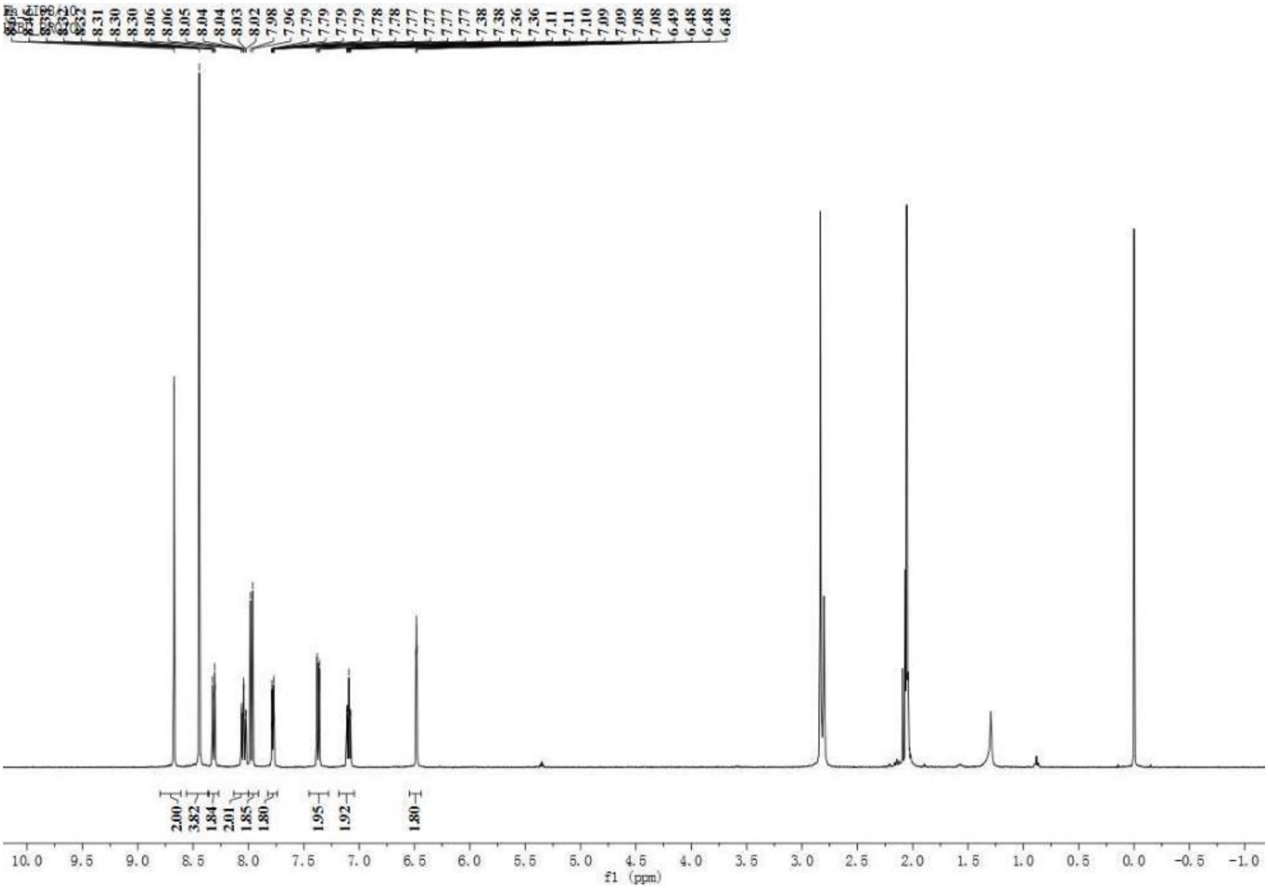

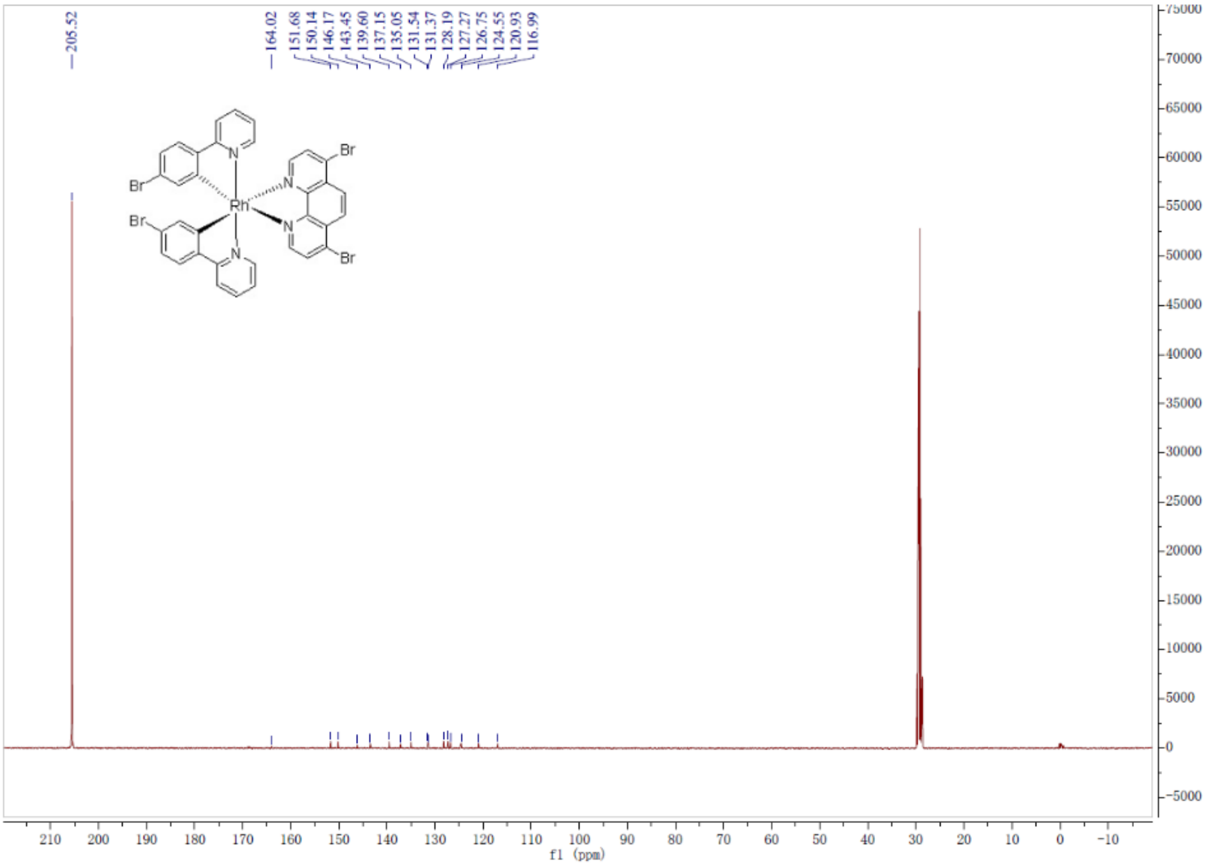
205.52
164.02
151.68
150.14
146.17
143.45
139.60
137.15
135.05
131.54
131.37
128.19
127.27
126.75
124.55
120.93
116.99
Br
Rh
f1 (ppm)

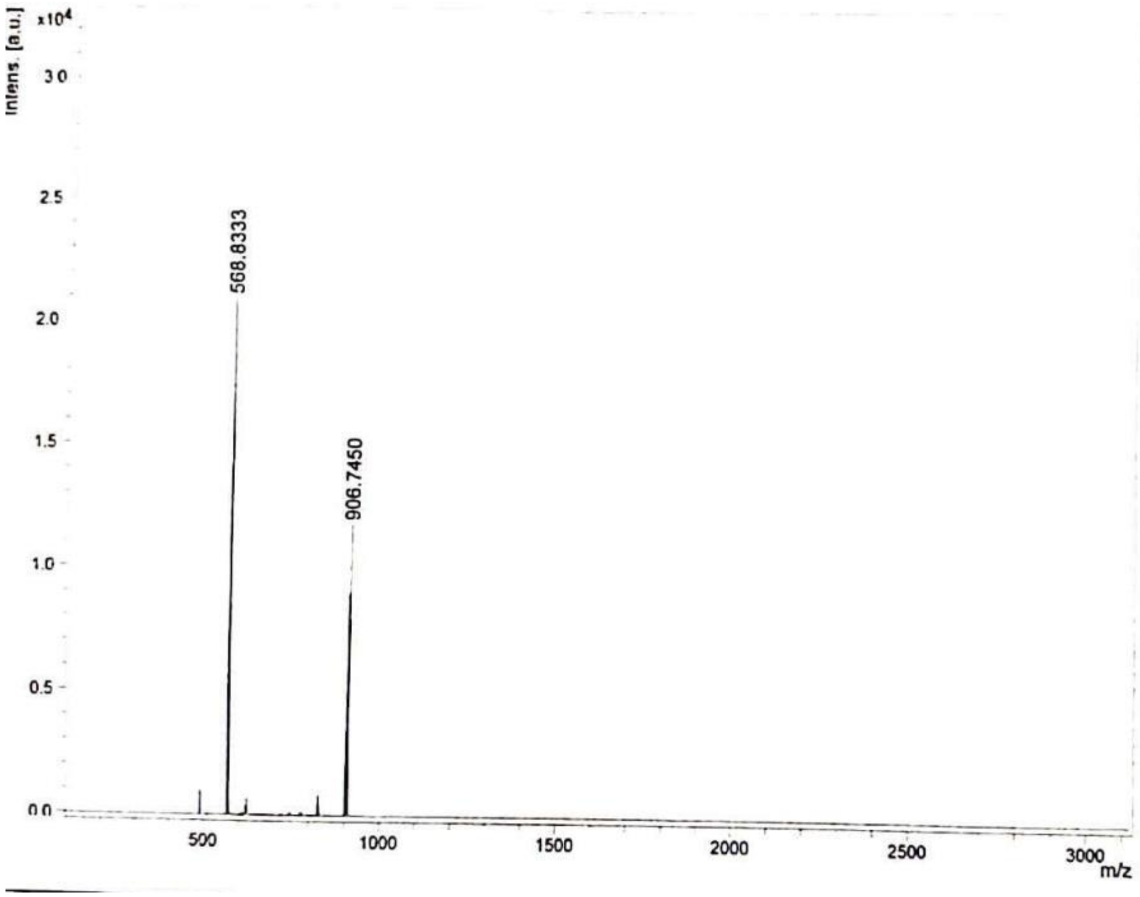
Intens. [a.u.]
x10^4
568.8333
906.7450
m/z

Complex **1l**. Yield: 66%. ^{1}H NMR (400 MHz, Acetone-d_6) δ 8.67 (s, 2H), 8.46 (d, J = 5.5 Hz, 2H), 8.41 (d, J = 5.6 Hz, 2H), 8.29 (dt, J = 8.3, 1.3 Hz, 2H), 7.99 – 7.89 (m, 4H), 7.81 (ddd, J = 5.8, 1.5, 0.7 Hz, 2H), 7.29 (dd, J = 8.4, 2.0 Hz, 2H), 7.03 (ddd, J = 7.3, 5.8, 1.4 Hz, 2H), 6.45 (d, J = 2.0 Hz, 2H). ^{13}C NMR (100 MHz, Acetone-d_6) δ 167.21, 152.95, 152.25, 150.89, 148.32, 144.46, 140.15, 137.52, 134.76, 132.72, 132.59, 129.18, 127.68, 126.95, 125.76, 125.10, 121.40, 73.14, 71.42. HRMS $[C_{34}H_{20}IrN_4Br_4]^+$ calculated: 996.8010, found: 996.7966. Anal. ($C_{34}H_{20}Br_4IrN_4PF_6$) C, H, N: calculated: 35.78, 1.77, 4.91; found: 35.94, 3.48, 6.07.

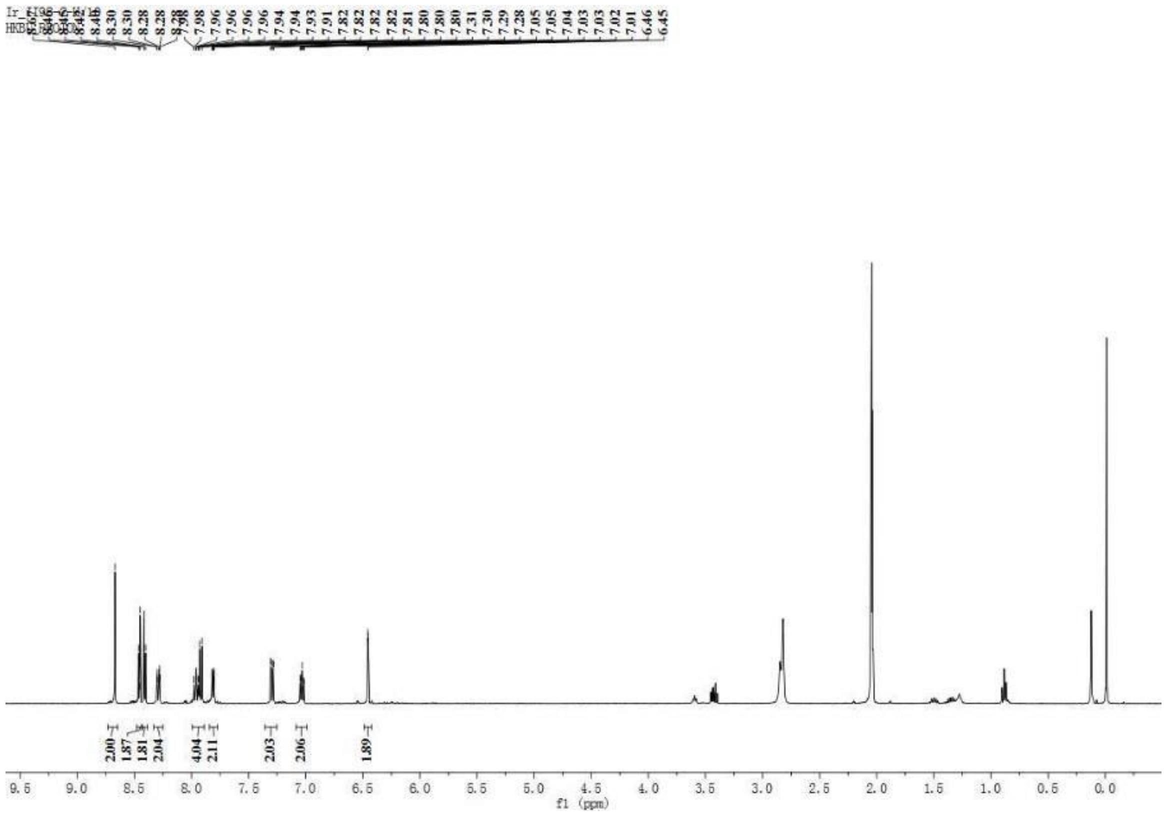

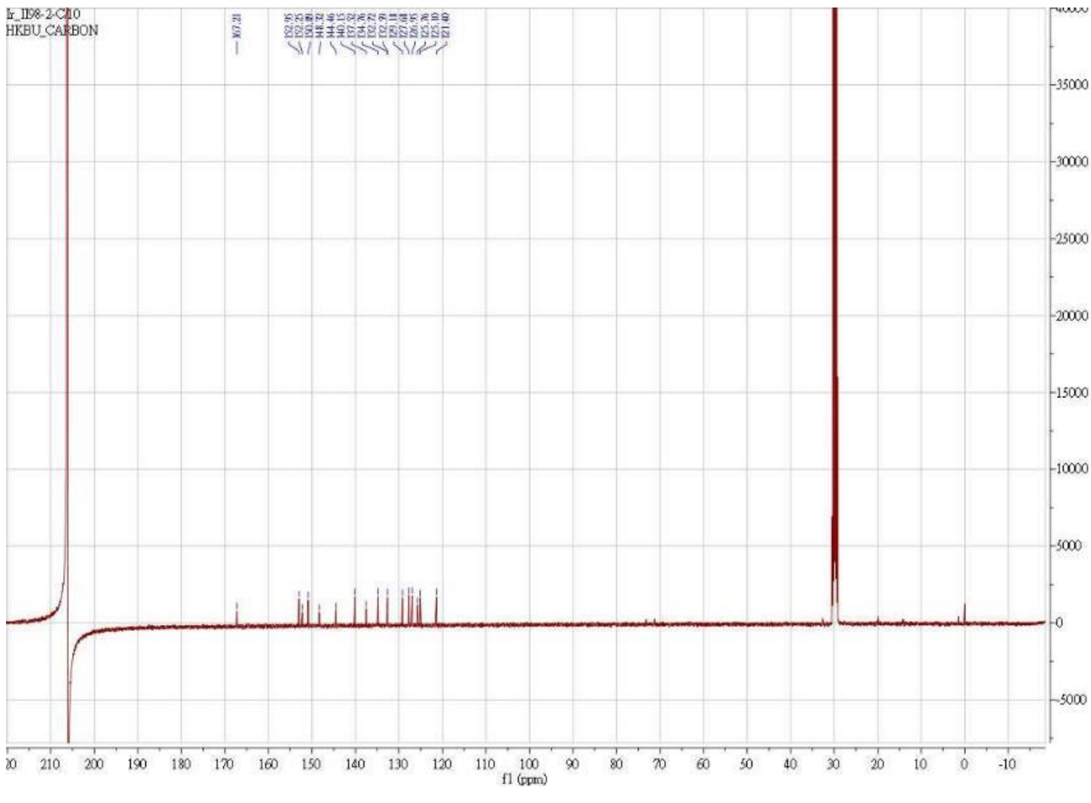

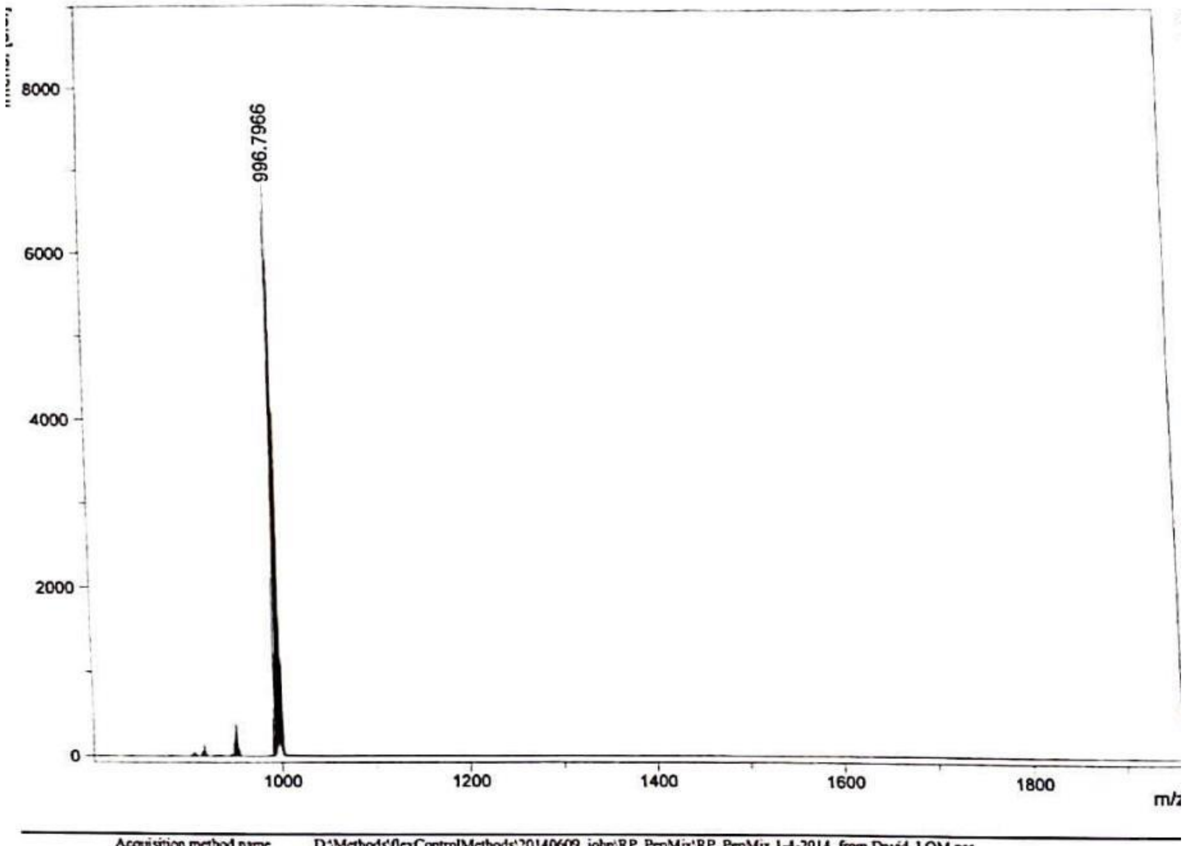

Acquisition method name D:\Methods\flexControlMethods\20140609_john\RP_PepMix\RP_PepMix 1-4-2014_from David_LOM.par
Sample name (file name prefix) Ir-II-98-DCTB\0_O17\1

Complex **1m**. Yield 60%. ^{1}H NMR (400 MHz, Acetone-d_6) δ 8.64 (dd, J = 5.2, 0.6 Hz, 2H), 8.38 – 8.31 (m, 2H), 8.28 (s, 2H), 8.11 – 8.02 (m, 4H), 8.00 (d, J = 8.4 Hz, 2H), 7.84 – 7.76 (m, 2H), 7.66 (p, J = 2.8 Hz, 10H), 7.38 (dd, J = 8.3, 2.0 Hz, 2H), 7.14 (s, 2H), 6.54 (dd, J = 2.0, 1.1 Hz, 2H). ^{13}C NMR (100 MHz, Acetone-d_6) δ 170.00, 169.68, 164.22, 163.23, 163.21, 151.75, 147.56, 146.24, 146.09, 142.37, 133.16, 133.14, 132.86, 129.07, 129.06, 128.64, 123.24, 123.02, 122.67, 120.89, 116.99, 106.73, 57.11, 22.60. HRMS $[C_{46}H_{30}RhN_4Br_2]^+$ calculated: 900.9872, found: 900.9836. Anal. ($C_{46}H_{30}Br_2IrN_4PF_6$) C, H, N: calculated: 48.65, 2.66, 4.93; found: 48.60, 2.58, 4.83.

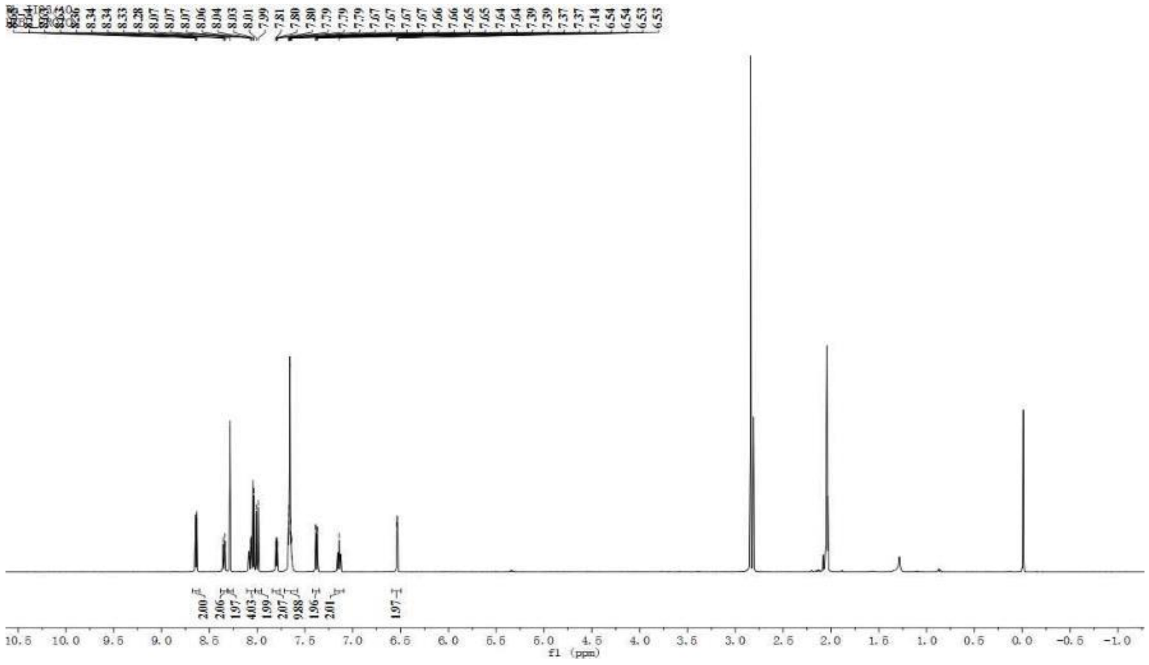

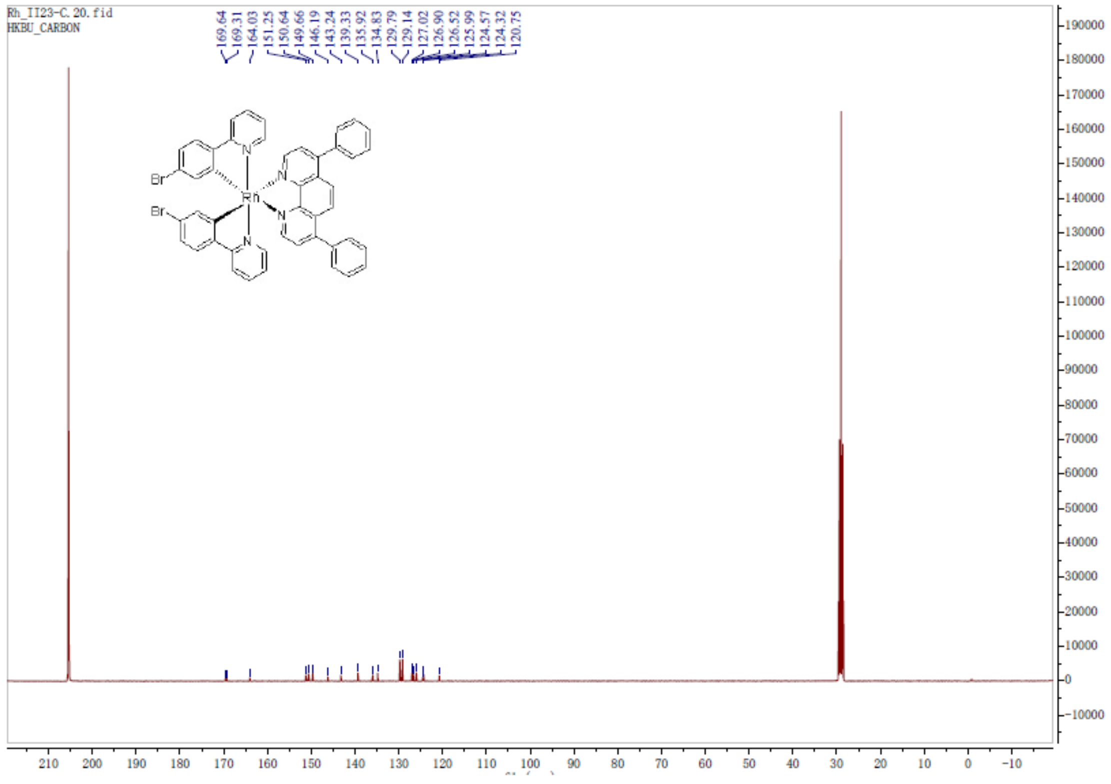
Rh_II23-C.20.fid
HKBU_CARBON
169.64
169.31
164.03
151.25
150.64
149.66
146.19
143.24
139.33
135.92
134.83
129.79
129.14
127.02
126.90
126.52
125.99
124.57
124.32
120.75
Br
Br
Rh
N

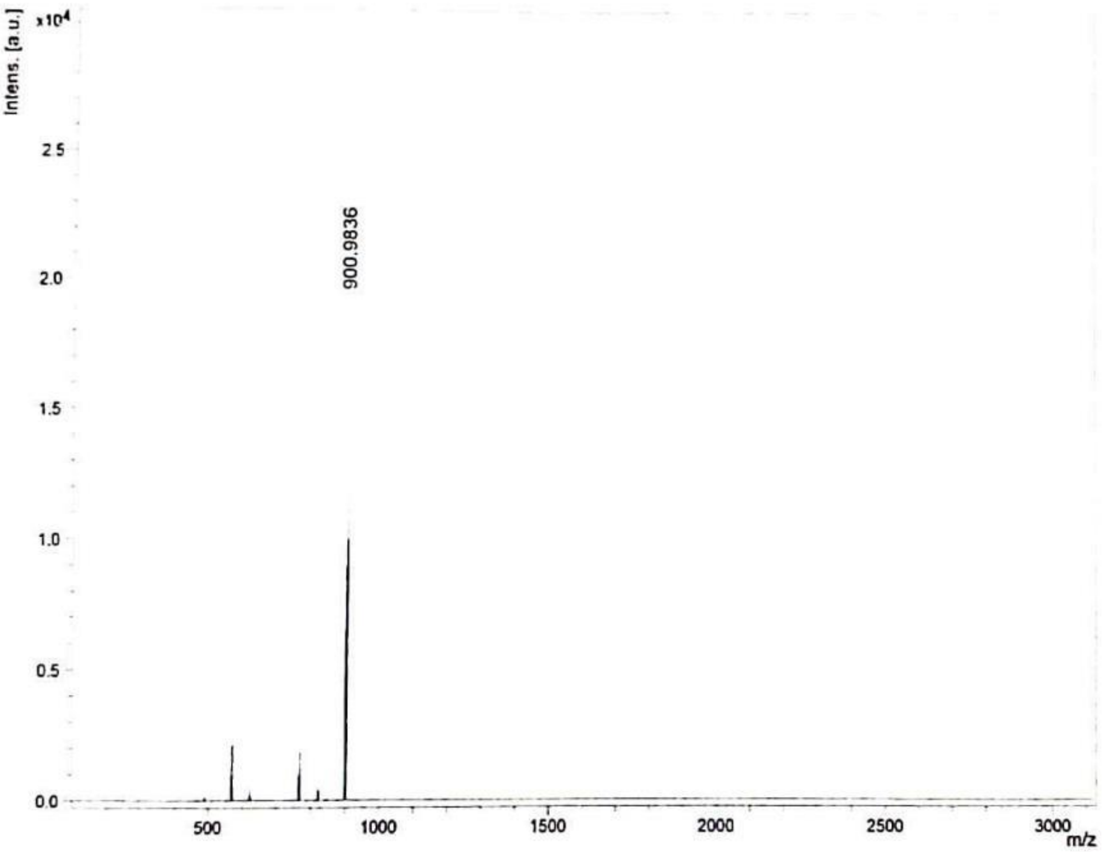
Intens. [a.u.]
x10^4
900.9836
m/z

Complex **1n.** Yield: 60%. ^{1}H NMR (400 MHz, Acetone-d_6) δ 8.61 (d, J = 5.3 Hz, 2H), 8.35 (dd, J = 8.2, 1.2 Hz, 2H), 8.31 (s, 2H), 8.07 (d, J = 5.3 Hz, 2H), 8.03 – 7.94 (m, 4H), 7.85 (dt, J = 5.7, 1.2 Hz, 2H), 7.73 – 7.62 (m, 10H), 7.32 (dd, J = 8.4, 2.0 Hz, 2H), 7.11 (ddd, J = 7.3, 5.8, 1.4 Hz, 2H), 6.53 (d, J = 2.0 Hz, 2H). ^{13}C NMR (101 MHz, Acetone) δ 167.43, 153.40, 152.10, 151.96, 150.61, 148.35, 144.50, 140.06, 136.60, 134.75, 130.74, 130.68, 130.45, 130.01, 128.25, 127.70, 127.21, 126.73, 125.78, 125.13, 121.43. HRMS $[C_{46}H_{30}IrN_4Br_2]^+$ calculated: 991.0446, found: 991.0440. Anal. ($C_{46}H_{30}Br_2IrN_4PF_6$) C, H, N: calculated: 52.80, 2.89, 5.35; found: 52.90, 2.95, 5.38.

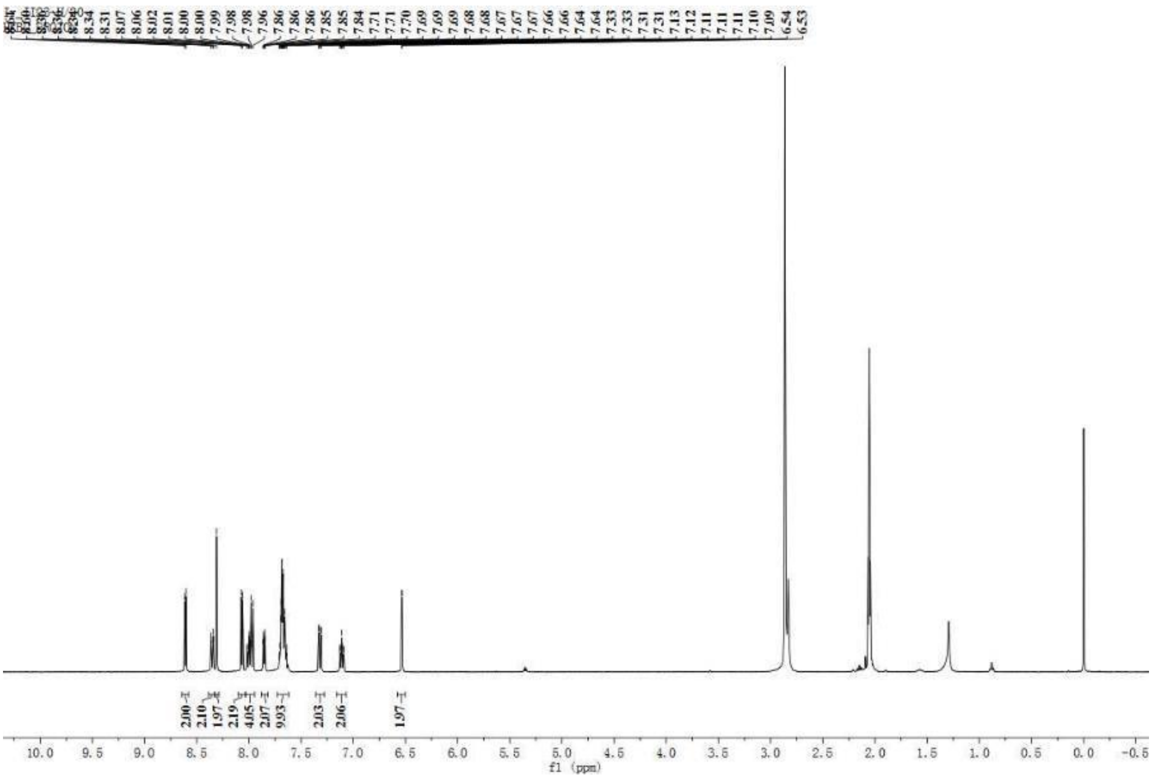

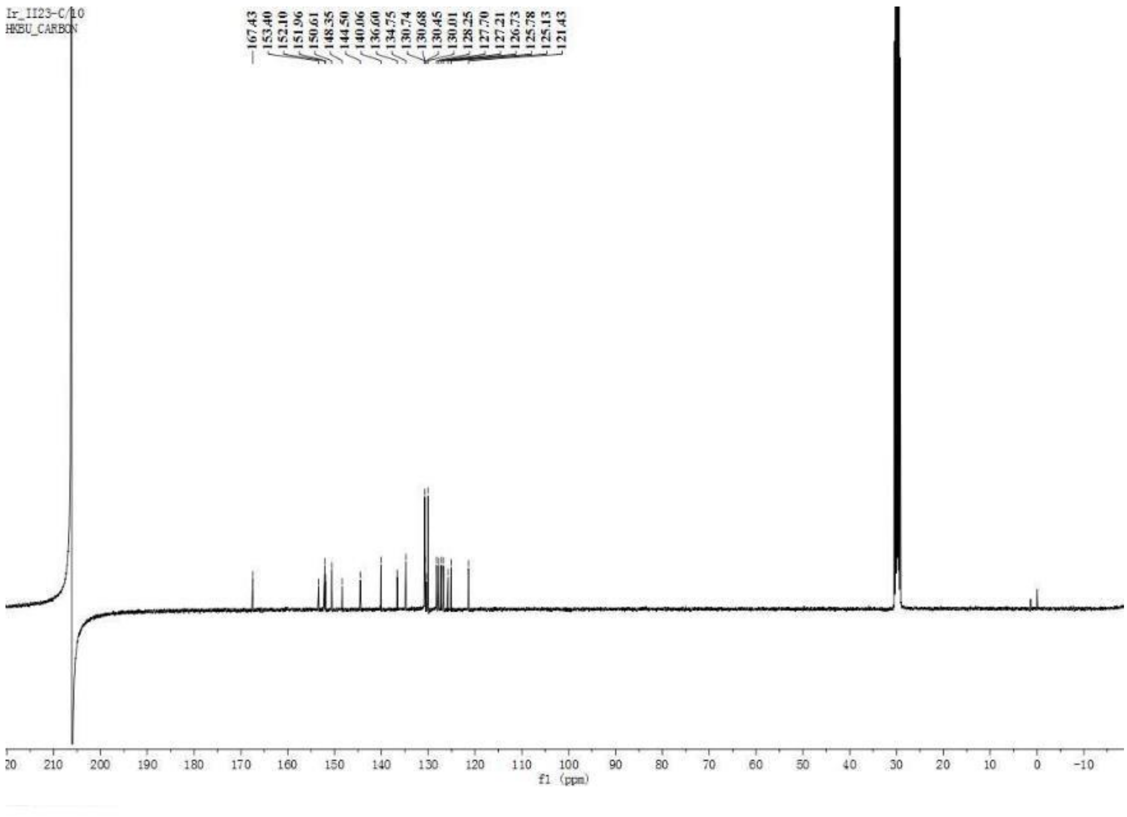
Ir_II23-C/10
HKBU_CARBON
167.43
153.40
152.10
151.96
150.61
148.35
144.50
140.06
136.60
134.75
130.74
130.68
130.45
130.01
128.25
127.70
127.21
126.73
125.78
125.13
121.43
f1 (ppm)

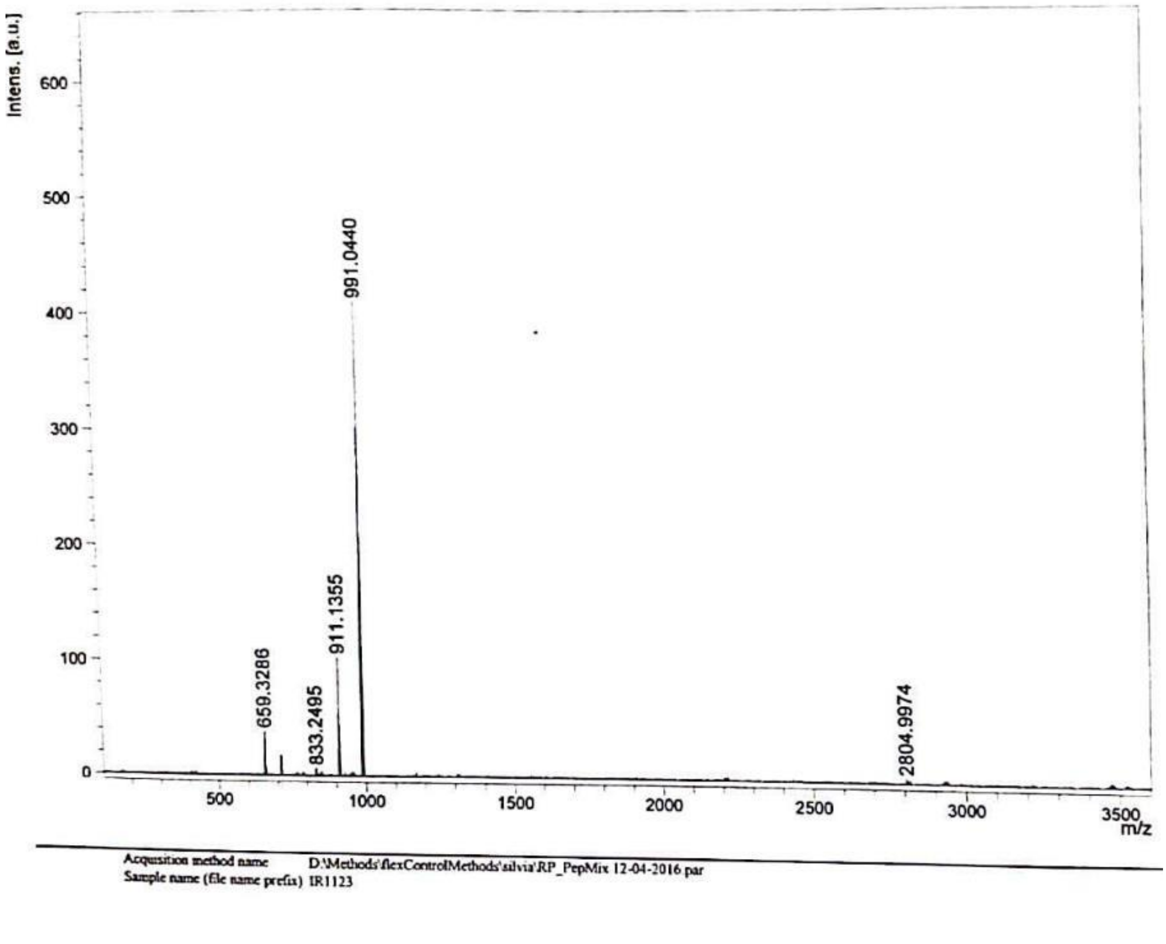
Intens. [a.u.]
991.0440
911.1355
659.3286
833.2495
2804.9974
m/z
Acquisition method name D:\Methods\flexControlMethods\silvia\RP_PepMix 12-04-2016.par
Sample name (file name prefix) IR1123

References

1. Buckley, D. L. *et al.* Targeting the von Hippel–Lindau E3 ubiquitin ligase using small molecules to disrupt the VHL/HIF-1α interaction. *Journal of the American Chemical Society* **134**, 4465-4468 (2012).
2. Jain, A. *et al.* Single-molecule pull-down for studying protein interactions. *Nature Protocols* **7**, 445 (2012).
3. Louche, A., Salcedo, S. P., Bigot, S. Protein–Protein Interactions: Pull-Down Assays. In: *Bacterial Protein Secretion Systems: Methods and Protocols* (ed^(eds Journet L, Cascales E). Springer New York (2017).
4. Malhotra, K., Alder, N. N. Reconstitution of Mitochondrial Membrane Proteins into Nanodiscs by Cell-Free Expression. *Methods in molecular biology (Clifton, NJ)* **1567**, 155-178 (2017).
5. Fareh, M. *et al.* Single-molecule pull-down for investigating protein–nucleic acid interactions. *Methods* **105**, 99-108 (2016).
6. Galdeano, C. *et al.* Structure-guided design and optimization of small molecules targeting the protein–protein interaction between the von Hippel–Lindau (VHL) E3 ubiquitin ligase and the hypoxia inducible factor (HIF) alpha subunit with in vitro nanomolar affinities. *Journal of medicinal chemistry* **57**, 8657-8663 (2014).
7. Yang, C. *et al.* Discovery of a VHL and HIF1α interaction inhibitor with in vivo angiogenic activity via structure-based virtual screening. *Chemical Communications* **52**, 12837-12840 (2016).
8. Van Molle, I. *et al.* Dissecting fragment-based lead discovery at the von Hippel-Lindau protein: hypoxia inducible factor 1α protein-protein interface. *Chemistry & biology* **19**, 1300-1312 (2012).
9. Liu, J. *et al.* Parkin targets HIF-1α for ubiquitination and degradation to inhibit breast tumor progression. *Nature communications* **8**, 1823 (2017).
10. Thoma, C. R. *et al.* VHL loss causes spindle misorientation and chromosome instability. *Nature cell biology* **11**, 994 (2009).
11. Botusan, I. R. *et al.* Stabilization of HIF-1 alpha is critical to improve wound healing in diabetic mice. *Proceedings Of the National Academy Of Sciences Of the United States Of America* **105**, 19426-19431 (2008).
